# Adherence to smartphone-delivered antimicrobial prescribing guidelines in hospitals in Lao PDR: A stepped-wedge cluster randomized controlled trial

**DOI:** 10.1371/journal.pmed.1005205

**Published:** 2026-08-27

**Authors:** Vilada Chansamouth, Danoy Chommanam, Sue J. Lee, Susath Vongphachanh, Manivanh Vongsouvath, Sommay Keomany, Viladeth Paphasiri, Chanthala Phamisith, Siho Sengsavang, Phouvieng Douangdala, Khamsay Detleuxay, Sommana Rattana, Bandith Soumphonphakdy, Valy Keoluangkhot, Mavuto Mukaka, Nicholas P. J. Day, H. Rogier van Doorn, Paul Turner, Mayfong Mayxay, Paul N. Newton, Elizabeth A. Ashley

**Affiliations:** 1 Lao-Oxford-Mahosot Hospital-Wellcome Trust Research Unit (LOMWRU), Mahosot Hospital, Vientiane, Lao PDR; 2 Microbiology Laboratory, Mahosot Hospital, Vientiane, Lao PDR; 3 Centre for Tropical Medicine & Global Health, Nuffield Department of Medicine, University of Oxford, Oxford, United Kingdom; 4 Mahidol Oxford Tropical Medicine Research Unit (MORU), Faculty of Tropical Medicine, Mahidol University, Bangkok, Thailand; 5 Department of Infectious Diseases, Alfred Health and School of Translational Medicine, Monash University, Melbourne, Australia; 6 Mahosot Hospital, Vientiane, Lao PDR; 7 Salavan Provincial Hospital, Salavan, Salavan Province, Lao PDR; 8 Xiengkhuang Provincial Hospital, Phonsavan, Xiengkhuang Province, Lao PDR; 9 Savannakhet Provincial Hospital, Kayson Phomvihane, Savannakhet Province, Lao PDR; 10 Vientiane Provincial Hospital, Viengkham, Vientiane Province, Lao PDR; 11 Luang Namtha Provincial Hospital, Luang Namtha, Luang Namtha Province, Lao PDR; 12 Department of Healthcare and Rehabilitation, Ministry of Health, Vientiane, Lao PDR; 13 Children’s Hospital, Vientiane, Lao PDR; 14 Oxford University Clinical Research Unit, Hanoi, Viet Nam; 15 Cambodia Oxford Medical Research Unit, Angkor Hospital for Children, Siem Reap, Cambodia; 16 Institute of Research and Education Development (IRED), University of Health Sciences, Ministry of Health, Vientiane, Lao PDR; 17 Saw Swee Hock School of Public Health, National University of Singapore (NUS), Singapore, Singapore; 18 Faculty of Infectious and Tropical Diseases, London School of Hygiene and Tropical Medicine, London, United Kingdom; Queen’s University, CANADA

## Abstract

**Background:**

Prescribing guidelines are a cornerstone of antimicrobial stewardship programs. Smartphone applications can increase access in low-and middle-income countries (LMICs). Following the release of the Lao national antimicrobial prescribing guidelines in 2021, this trial compared adherence to these guidelines delivered by a smartphone application versus the paper-based version among hospitalized patients and outpatients in six hospitals in Laos.

**Methods and findings:**

An open cohort stepped-wedge cluster randomized controlled 3-step trial was conducted from 2021–2022. The intervention was a smartphone application-based prescribing guideline and antimicrobial stewardship (AMS) training. The reference was a paper-based version of the same guidelines. Adherence was defined as prescriptions using the correct antimicrobial(s) and correct dose based on the provided prescribing guidelines. The primary outcome was assessed via repeated point prevalence surveys (PPS) at ~4-month intervals. Data were analyzed using a mixed-effects regression model, accounting for time and clustering. Among 3,561 hospitalized patient prescriptions, overall guideline adherence was 17.0% (95% confidence interval or CI [15.0,19]) in the reference group, and 25.6% (95% CI [23.1,28.3]) in the intervention group. After accounting for time and cluster effects and adjusting for confounders (sequence of intervention, age, gender, prescribing indication, and hospital department), adherence did not differ by guideline delivery mode (adjusted odds ratio (aOR)=1.26 (95% CI [0.8,1.9]; *p* = 0.276). Similar findings were observed in outpatient settings (aOR = 0.91 (95% CI [0.7,1.1]; *p* = 0.406)). Outpatient adherence varied by time of intervention introduction, with the first group of hospitals exposed to the intervention showing higher odds of adherence (32.1% [302/940]; 95% CI [29.1,35.2]) compared with the last group (19.1% [241/1,261]; 95% CI [16.9,21.4]) with aOR = 1.95 (95% CI [1.4,2.7]; *p* < 0.001). Prescribing in this study was assessed using patients’ medical records; as prescriptions could not be reliably linked to individual prescribers, changes in individual prescribing behavior could not be evaluated.

**Conclusion:**

This study demonstrated that smartphone technology can deliver antimicrobial prescribing guidelines in an LMIC like Laos. However, the trial did not demonstrate the pre-specified improvement in guidelines adherence with the smartphone app and AMS training compared with paper-based guidelines. While treatment guidelines underpin antimicrobial stewardship efforts, adherence depends on multiple factors beyond guideline availability. The trial was registered at clinicaltrials.gov (NCT04914793).

## Author summary

**Why was this study done?:**

- Inappropriate use of antimicrobials is a key driver of antimicrobial resistance (AMR). Providing clear, accessible guidelines is an essential step toward improving prescribing practices.
- Some evidence suggests that delivering guidelines via simple technologies, such as smartphone applications, can support better antimicrobial use and hospital stewardship. However, most reports come from high-income country settings.

**What did the researchers do and find?:**

- We conducted a stepped-wedge cluster randomized controlled trial in six hospitals across Laos to compare adherence to newly introduced antimicrobial prescribing guidelines delivered via a smartphone application, with training on antimicrobial use principles and app use, versus a paper-based version, with training on the use. Regular point prevalence surveys were conducted to monitor antimicrobial prescribing.
- A total of 3,561 inpatient and 3,905 outpatient antimicrobial prescriptions were assessed between 2021 and 2022. Guideline adherence remained low and did not differ between hospitals using the paper-based guidelines and those using the app-based version alongside training on antimicrobial use principles. Low guideline adherence was partly explained by the persistently high proportion of inappropriate antimicrobial use for surgical and obstetric prophylaxis.

**What do these findings mean?:**

- This trial offers a comprehensive evaluation from the development of prescribing guidelines to their implementation and the assessment of their impact on adherence. The study provides important data on antimicrobial prescribing practice and guideline adherence in hospitals in Laos, offering a foundation for future interventions and stewardship programs.
- Although the trial findings did not demonstrate a marked improvement in adherence following the intervention, they showed that a simple technology-based approach is feasible to implement to deliver prescribing guidelines in low- and middle-income countries (LMICs) like Laos.
- The results highlight that antimicrobial prescribing guideline availability alone does not guarantee adherence, suggesting that a multifaceted approach is needed to effectively support the use of prescribing guidelines.
- An important limitation of this study is that prescribing was assessed using patients’ medical records, and prescriptions could not be reliably linked to individual prescribers. Therefore, changes in prescribing behavior at the individual prescriber level after the guideline implementation could not be evaluated.

## Abstract 3

In this stepped-wedge cluster randomized trial, Vilada Chansamouth and colleagues evaluate whether a smartphone application-based antimicrobial prescribing intervention can improve adherence to national prescribing guidelines compared with paper-based guidelines across six hospitals in Laos.

## Introduction

Antimicrobial resistance (AMR) is a major global health threat, estimated to be associated with 4.71 million deaths in 2021 [1]. Inappropriate use of antibiotics is one of the main drivers of AMR, contributing to the emergence and spread of resistant bacteria [2]. To address this, local evidence-based guidelines are essential tools for optimizing health delivery and patient care in countries globally. In 2023, the Tracking AMR Country Self-Assessment Survey (TrACSS), an initiative of the Quadripartite Collaboration for One Health, revealed that 113/177 (64%) surveyed countries had established national guidelines for appropriate use of antimicrobials [3]. In high-income countries (HICs), institutional prescribing guidelines are common, in contrast to low- and middle-income countries (LMICs) which often refer to national guidelines. In many LMICs using local evidence-based data to develop guidelines is less common and guidelines are not widely available [4].

Mobile health or mHealth is used increasingly across various healthcare domains, such as diagnostics and clinical decision support, delivery of medical education, chronic disease management, or health tracking [5]. While these applications offer great benefits, most require a paid subscription for full access, which can be a barrier to use in many LMICs. A systematic review of 41 studies to understand how physicians used smartphones or tablets in their clinical settings from 2012 to 2021 showed that 80% of participants (36–3,306 participants/study) used their smartphones during their clinical practice and that use has been increasing [6]. The most common use of smartphone devices was for searching for drug information, for example: dosages, preparation, and indications for use. Accessing medical calculators emerged as another common use. Smartphone applications (app) have gained attention across various medical fields due to their convenience, ability to support clinical decision-making, improve patient records and optimize workflow [6–8]. However, most reports come from HICs. Recent studies have highlighted the potential of smartphone apps to improve adherence to clinical guidelines across various healthcare settings [9–17].

Laos is an LMIC in Southeast Asia with an estimated population of 7.66 million in 2023. In 2022, the life expectancy was 68 years, and in 2021, there were only 0.3 physicians per 1,000 people [18]. The country lacks a national antimicrobial stewardship program. A four-year hospital antimicrobial use surveillance (2017–2020) revealed that 71% of hospitalized patients received at least one antimicrobial [19]. The national antimicrobial prescribing guideline for Laos was developed between 2019 and 2020, in both paper-based and smartphone application-based formats. Dissemination of these guidelines began in 2021. This trial compared the proportion of antimicrobial prescriptions adhering to the guidelines delivered via smartphone app versus the paper-based version among inpatient and outpatient settings in six hospitals in Laos.

## Methods

### Study design and participants

The LAMPA (Lao AntiMicrobial Prescribing Application) trial was a three-step open cohort stepped-wedge cluster randomized controlled trial (SW-CRT) conducted across one central and five provincial hospitals in Laos (S1 Fig and S1 Table). The hospitals were selected based on their geography (northern, central, and southern Laos), and previous participation in point prevalence surveys (PPS) of hospital antimicrobial use [19]. The SW-CRT design, where an intervention is introduced sequentially at different sites, was chosen as the most suitable for the context, as it required fewer sites while allowing for similar observational periods during both control and intervention phases. Given that the interventions were delivered by a single team traveling to each cluster in sequence, logistical constraints such as limited personnel, time, and travel requirements, also influenced the implementation schedule [20]; therefore, a staged implementation approach was more practical than a parallel trial.

Outcome data were collected using PPS. PPSs are widely used to monitor antimicrobial use and prescribing practices and provide a standardized and feasible method for antimicrobial surveillance and stewardship activities, particularly in LMICs, where electronic prescribing records are not routinely available, and continuous data collection is otherwise costly and time-consuming [21].

The trial was approved by the University of Health Sciences Ethics Committee, Lao PDR (Reference: 105/REC, 27 October 2020) and the Oxford Tropical Research Ethics Committee, University of Oxford (OxTREC Ref: 541-20, 16 July 2020). This trial was registered at clinicaltrials.gov (NCT04914793), and the study proposal is provided in S1 Text. The trial was registered a few months after commencement due to an initial misinterpretation of registration requirements. As this was a cluster randomized controlled trial without individual participant randomization and evaluated the impact of antimicrobial prescribing guidelines rather than direct health outcomes, registration was not initially undertaken. Following further consultation, a conservative approach was adopted and the trial was registered. The study involved collecting anonymized data from patients’ medical records, with no direct contact with patients or prescribers. Interaction with prescribers occurred only when clarification was needed due to unclear handwriting. As no identifiable information was collected and patient involvement was indirect, written informed consent was not required.

### Randomization and masking

The six hospitals were pre-defined into three groups of two based on geographical location and convenience for conducting the surveys. The first hospital group included two hospitals in the north of Laos (Luang Namtha (LNT) and Xiengkhuang (XK)), the second group included hospitals in the center (Mahosot (MS) which is located in Vientiane Capital and Vientiane Province (VTP)). The third group included hospitals in the south (Savannakhet (SVK) and Salavan (SV). Simple random sampling (lottery method) was used to determine the sequence in which the three pre-defined groups would receive the intervention (i.e., first, second, and third rollout steps). Randomization was applied only to the sequence of implementation across groups, rather than the actual calendar start dates. The allocation of the implementation sequence was carried out by an individual not involved in study implementation. Neither hospitals nor investigators were masked to the introduction of the intervention.

### Intervention

The intervention included a smartphone application version of the newly developed local antimicrobial prescribing guidelines, accompanied by a training session in their use and good prescribing practices. The training content was the same as that provided in the antimicrobial stewardship section of the guidelines. This set of activities is collectively referred to as “App-G” or the app group for simplicity. The reference (control) was the paper-based version of the same prescribing guidelines with training in their use only, and is referred to as “PP-G” or the paper-based group.

The guidelines were in Lao language, separated into pediatric and adult editions. There were 90 infectious diseases topics for treatment of infections and 12 topics addressing surgical and medical prophylaxis in the adult guidelines. The pediatric guideline included 66 infectious diseases topics for treatment of infections and 13 topics for surgical and medical prophylaxis. Both pediatric and adult guidelines included an antibiotic stewardship section. The guidelines were endorsed by the Lao Ministry of Health. The MicroGuide platform (since rebranded as Eolas Medical app [22]) was used to deliver the app-based guidelines. Every 4 months, after a hospital group was randomly assigned to the intervention phase, the app was installed on prescribers’ personal smartphones by the research team. On the same day, a training session on good prescribing practices, based on the guideline content, was provided to all prescribers at participating hospitals.

Although the hospitals were geographically distant, travel between hospitals and communications between prescribers in Laos is common. To minimize contamination, at the start of the intervention phase, participants were requested to avoid sharing use of the app to anybody outside their hospitals while they were still under evaluation. The research team installed the apps using a code, thereby restricting access to the app to prescribers inside the study sites. The system, security and app maintenance were controlled by the MicroGuide team and the research team of the project.

### Outcomes

The primary outcome was the proportion of antimicrobial prescriptions adherent with prescribing guidelines delivered by smartphone app with a training in guidelines use, accompanied by “good antimicrobial prescribing practices” training versus paper-based prescribing guidelines with a training in guidelines use only, with all hospitals having access to both intervention and reference by the end of the trial. Secondary outcomes included: 1) the proportion of hospitalized patients receiving antimicrobial prescriptions; 2) total antimicrobial defined daily doses (DDDs); 3) the proportion of intravenous antimicrobial prescriptions; 4) prescribers’ AMS knowledge; and 5) prescriber satisfaction with the guidelines, each assessed before and after App-G implementation. Outcomes four and five will be reported separately.

Full adherence with the guidelines was defined as the prescriptions with correct antimicrobial agent(s) and correct dose. For elective surgical prophylaxis antimicrobial duration was incorporated in the definition since it should only be administered pre-operatively or intra-operatively (using the correct antimicrobial agent(s), correct dose and correct duration based on the provided antimicrobial prescribing guidelines). Nonadherence to the guidelines was defined as any deviation from the guidelines, including use of an incorrect antimicrobial agent(s), or use of the correct agent(s) with an incorrect dose, and/or incorrect duration for elective surgical prophylaxis. The outcomes of hospitalized patients were measured using PPS on hospital antimicrobial use. Hospital records of all hospitalized patients of all wards at 8:00 AM on the survey day were reviewed. Records documenting the use of antimicrobial agent were included, and total number of inpatients in each ward was recorded to estimate antimicrobial use. Patients admitted after 8:00 AM on the survey day were excluded. Patients receiving ongoing antimicrobial regimens (e.g., every 24 hours, every 48 hours, or once weekly) were also included. Additional details of PPS methodology for hospitalized patients have been described previously [19]. All outpatient (OPD) records and prescriptions from patients attending the pediatric, medicine, and surgical OPDs of all participating hospitals between 8:00 AM and 4:00 pm on the survey days were screened for antimicrobial prescriptions. If the same outpatient returned for consultation during the same survey period, this patient was included in the survey and each visit was defined as a new consultation. Topical antimicrobials were not included in the antimicrobial use calculation for both OPD and hospitalized patients. Data were retrospectively collected for five OPD consultation days (excluding weekends) to ensure that the number of prescriptions was representative of routine OPD activity.

### Procedures

Baseline antimicrobial prescription data were collected at month-0. Then, pediatric and adult versions of the PP-G were distributed to all prescribers at participating hospitals, followed by a session focused solely on guideline use. No training on diagnosing infectious diseases or prescribing antimicrobials was provided. Once the PP-G was introduced at all sites at the start of the trial, the six participating hospitals officially entered the PP-G period. At month-4, the intervention was introduced to the first hospital group. Subsequently, at ~4-month intervals, each group of hospitals crossed over from the reference phase to the intervention phase until all groups had implemented the intervention. This resulted in an overall 1:1 ratio of intervention to reference observation periods by the end of the trial (month 16). During the App-G period, prescribers in participating hospitals still had access to paper-based guidelines distributed at the start of the study. A PPS was conducted just before each hospital transitioned to the intervention (App-G) period (at months 4, 8, and 12 after initial PP-G introduction). By month-16, all hospitals had entered the intervention period, for at least 4 months.

### Sample size calculation

A point prevalence survey of hospital antimicrobial use in four general hospitals in Laos in 2017 showed 13% adherence to national treatment guidelines. However, as previous guidelines contained limited information on antibiotic prescribing, reliable baseline data were lacking. The sample size of six hospitals was calculated assuming 30% of antimicrobial prescription adherence with the paper-based prescribing guideline (reference), an intra-cluster correlation coefficient of 0.05, and an average of 40 doctors per hospital. Using the Stata “steppedwedge” package [23] (S2 Text), it was estimated that this would provide more than 80% power to detect a 15% difference after introduction of the smartphone app prescribing guidelines at a 0.05 significance level.

### Statistical analysis

The consolidated standards of reporting trials (CONSORT) extension for the SW-CRT was followed (S1 Checklist) [24]. Analyses followed an *a priori* statistical analysis plan (S3 Text). All analyses were done according to intention-to-treat principles. Normally distributed continuous baseline characteristics were summarized with means and standard deviation or 95% confidence intervals (CIs). Skewed continuous data were summarized using median and inter-quartile range (± range). Categorical data were presented as frequency and percentages. Test of trends, Chi-square or Fisher’s exact tests were used as appropriate. A mixed-effects logistic regression model was used to account for the hierarchical structure of data and repeated measurements over times within clusters. We followed methodological guidance for stepped-wedge trial with binary outcomes [25,26]. The model compared the proportion of antimicrobial prescription adherence measured in all steps in the App-G from month 4 to month 16 with all steps in the PP-G from month-0 to month-12. The binary outcome (adherence) was modeled with intervention group as the primary exposure. The model included fixed effects for age group, gender (male or female), hospital department (surgery, intensive care, pediatrics, obstetrics and gynecology, medicine, and other), prescribing indication (prophylaxis or treatment), and sequence (1, 2, or 3). The unit of analysis was indication and random effects for patient and hospital-time were included.

Given the small number of clusters, which may affect the reliability of standard errors in mixed-effects models, results were interpreted with caution, with emphasis on effect estimates and CIs rather than statistical significance alone. Odds ratios (OR) and corresponding 95% CI are reported. Tests of significance were performed at the 5% level. Hospitalized patient data and OPD data were analyzed separately. Analyses were performed using STATA 18 (StataCorp, College Station, Texas, USA).

### Role of the funding source

This study was funded by the Wellcome Trust of Great Britain [Grant number: 214207/Z/18/Z] awarded to VC. The funder’s website is https://wellcome.org/. The funder had no role in study design, data collection and analysis, decision to publish, or preparation of the manuscript.

ChatGPT (OpenAI; GPT-5.5) was used only to support grammar and language editing. All content was checked and revised by the authors, who are fully responsible for the final manuscript.

### Inclusive in global health research

Additional information regarding the ethical, cultural, and scientific considerations specific to inclusivity in global research is included in the Supporting Information (S2 Checklist).

## Results

**Fig 1:**
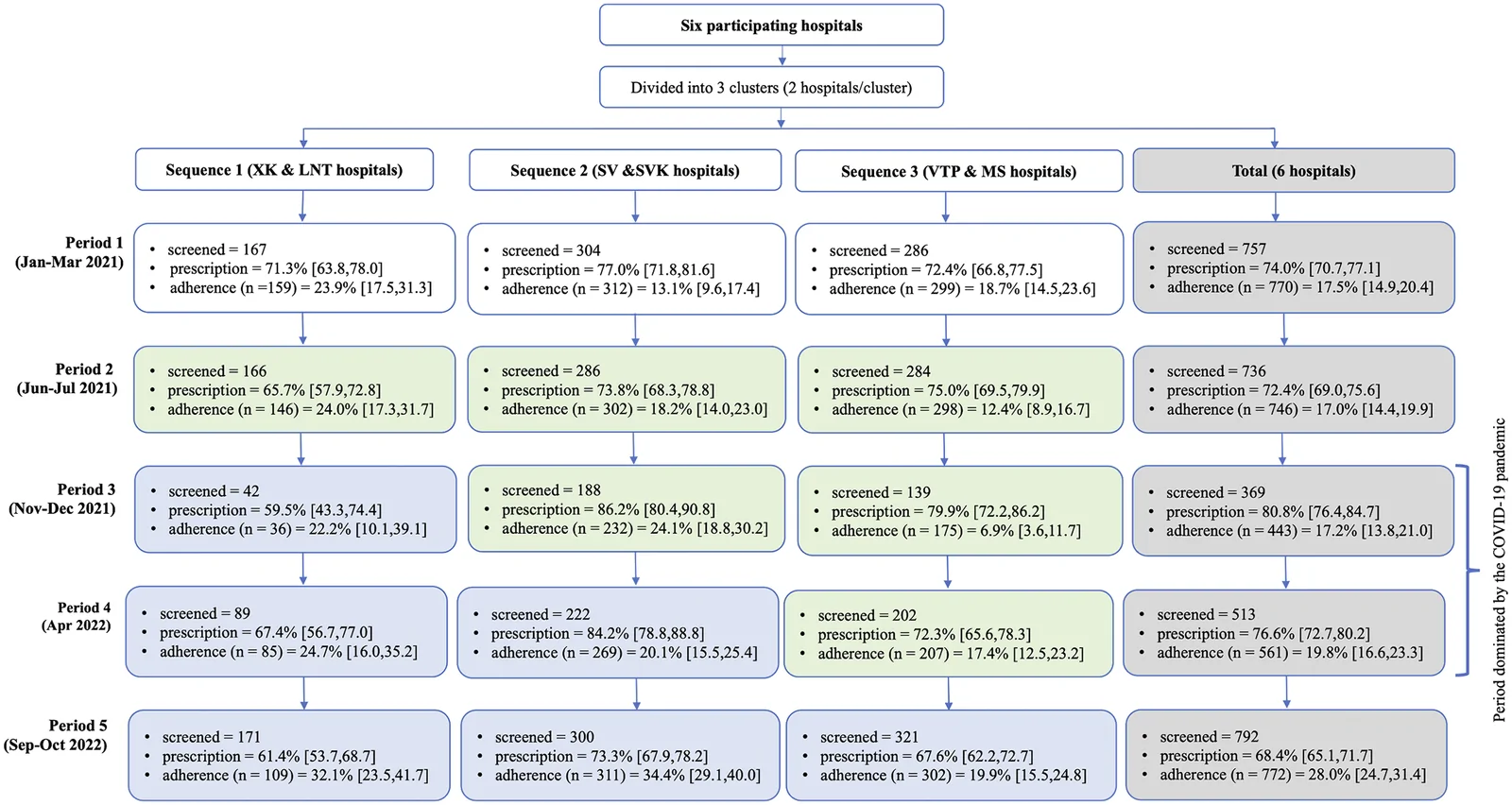
Lao AntiMicrobial Prescribing Application (LAMPA) trial profile for hospitalized patients. **Note**: White area (*n* = 852 prescriptions) = group with no antimicrobial prescribing guidelines, green area (*n* = 1,492 prescriptions) = group with paper-based version of prescribing guidelines (PP-G), blue area (*n* = 1,217 prescriptions) = group with application-based version of prescribing guidelines and antimicrobial stewardship training (App-G); XK = Xiengkhuang; LNT = Luang Namtha; SV = Salavan; SVK = Savannakhet; VTP = Vientiane Province; MS = Mahosot; screened = Number of screened patient’s documents; prescription = Percentage of patients prescribed an antimicrobial [95% CI]; adherence = Percentage of antimicrobial prescriptions fully adherent to the guidelines [95% CI]; *n* = Number of prescriptions with sufficient information to assess guideline adherence; Period 1 = Pre-guideline, Period 2 = Month-6 after PP-G implementation (all hospitals), Period 3 = First survey after App-G implementation in Xiengkhuang and Luang Namtha or month-11 after PP-G introduction, Period 4 = Second survey post-App-G for Xiengkhuang and Luang Namtha, first survey post-App-G for Salavan and Savannakhet, or month-17 after PP-G introduction, Period 5 = final survey after all clusters had App-G access or month-22 after PP-G introduction.

Between January and March 2021, the SW-CRT was initiated in six hospitals across Laos, during which baseline data collection (pre-guideline) and implementation of the PP-G took place. The median duration for these activities was 3.5 (range: 2–8) days. During the study period, 383/507 (75.5%; 95% CI [71.6,79.2]) prescribers had access to paper-based guidelines, while 362/477 (75.9%; 95% CI [71.8,79.7]) had access to the app-based guidelines (S2 and S3 Tables). The trial operated over 22 months, from January 2021 to October 2022 (Figs 1 and 3). Patient prescribing data were collected during five rounds of PPSs conducted throughout the trial period. The baseline survey was conducted between January and March 2021 before the implementation of PP-G. The second survey was conducted in June-July 2021, immediately before the implementation of the App-G in the first group of hospitals (XK and LNT; sequence-1). The third survey was conducted in November-December 2021, before App-G implementation in the second group (SV and SVK; sequence-2). The fourth survey was conducted in April 2022, before App-G implementation in the final group of hospitals (VTP and MS; sequence-3). The final survey was conducted between September and October 2022, by which time all hospitals had had access to App-G for at least 5–6-months. The interval between surveys was ~5–6 months, which was longer than expected due to COVID-19 pandemic restrictions. However, each PPS was completed before hospitals transitioned to the next phase, ensuring that outcome data were collected before exposure to the intervention (Fig 1 for hospitalized patients and Fig 3 for outpatients). A total of 3,167 inpatient records (median 95, range: 8−225 per survey per hospital) and 13,711 OPD prescriptions/records (median 377, range: 79−1,356 per survey per hospital) were screened for antimicrobial use across six hospitals over a 22-month period (S4 Table).

### Hospitalized patients

The percentage of inpatients prescribed antimicrobials was similar across all groups, at 74.0% in the pre-guideline group (560/757; 95% CI [70.7,77.1]), 75.2% in the PP-G group (952/1,265; 95% CI [72.8,77.6]), and 71.1% in the App-G group (814/1,145; 95% CI [68.4,73.7]) (Fig 1). Most patients were aged ≥15 years for all groups and nearly half of all patients received more than one antimicrobial. Treating infections was the most common prescribing indication for all groups and nearly all prescriptions were empirical (Table 1). The most commonly prescribed antimicrobials across all patient groups (S5 Table) and wards (S2 Fig) were cephalosporins (45.7% [1,629/3,561]; 95% CI [44.1,47.4]), followed by nitroimidazoles (only metronidazole is available in Laos: 18.4% [656/3,561]; 95% CI [17.2,19.7]), penicillins (13.5% [479/3,561]; 95% CI [12.3,14.6]), aminoglycosides (9.1% [323/3,561]; 95% CI [8.1,10.1]), and fluoroquinolones (2.5% [90/3,561]; 95% CI [2.0,3.1]). WHO AWaRe (Access, Watch, Reserve) [27] Access group antibiotics accounted for fewer than 50% of prescriptions before and after guideline introduction (S6 Table). Access group antibiotics were used more in children aged <15years (56.6%; 415/733 prescriptions; 95% CI [52.9,60.2] than in adults aged ≥15years (45.9%; 1,213/2,700 prescriptions; 95% CI [43.0,46.8]; *p* < 0.001; and even more in younger children aged ≤1year (68.7%; 281/409 prescriptions; 95% CI [64.0,73.2] compared to older children aged >1–14 years (41.4%; 134/324 prescriptions; 95% CI [35.9,46.9]; *p* < 0.001 (S3 Fig). Focusing solely on treatment of infections, the main prescribing indications, regardless of age, were pneumonia (19.2% [378/1,971]; 95% CI [17.5,21.0], sepsis (12.1% [238/1,971]; 95% CI [10.7,13.6], and appendicitis (10.4% [204/1,971]; 95% CI [9.0,11.8]) (S4 Fig).

**Table 1:** Characteristics of antimicrobial prescriptions in six hospitals in the pre-guideline, paper-based, and app-based guideline groups among hospitalized patients and outpatients.

Characteristics	Pre-guideline group		Paper-based group		App-based group	
	Inpatients *n* (%)	Outpatients *n* (%)	Inpatient *n* (%)	Outpatients *n* (%)	Inpatient *n* (%)	Outpatients *n* (%)
Screened patient documents - *n*	757	3,076	1,265	5,597	1,145	5,038
Patients with antimicrobial(s) prescribed - *n* (%)^a^	560 (74.0)	841 (27.3)	952 (75.2)	1,184 (21.2)	814 (71.1)	1,459 (28.9)
Antimicrobial use by hospital - *n/N* (%)^b^						
Xiengkhuang Provincial Hospital (XK)	79/111 (71.2)	134/274 (48.9)	74/110 (67.3)	124/438 (28.3)	129/202 (63.9)	358/930 (38.5)
Luang Namtha Provincial Hospital (LNT)	40/56 (71.4)	46/79 (58.2)	35/56 (62.5)	99/288 (34.4)	61/100 (61.0)	145/560 (25.9)
Salavan Provincial Hospital (SV)	72/86 (83.7)	248/464 (53.5)	118/151 (78.2)	372/1017 (36.6)	139/204 (68.1)	531/971 (54.7)
Savannakhet Provincial Hospital (SVK)	162/218 (74.3)	77/378 (20.4)	255/323 (78.9)	62/519 (11.9)	268/318 (84.3)	79/645 (12.3)
Vientiane Provincial Hospital (VTP)	70/94 (74.7)	84/750 (11.2)	167/217 (76.9)	153/1037 (14.8)	59/96 (61.5)	105/576 (18.2)
Mahosot Hospital (MS)	137/192 (71.4)	252/1131 (22.3)	303/408 (74.3)	374/2298 (16.3)	158/225 (70.2)	241/1356 (17.8)
Antimicrobial use by sequence of intervention implementation - *n* (%)						
Sequence 1 (XK + LNT)	119 (71.3)	180 (50.9)	109 (65.7)	223 (30.7)	190 (62.9)	503 (33.8)
Sequence 2 (SV + SVK)	234 (76.9)	325 (38.6)	373 (78.7)	434 (28.3)	407 (77.9)	610 (37.8)
Sequence 3 (VTP + MS)	207 (72.4)	336 (17.9)	470 (75.2)	527 (15.8)	217 (67.6)	346 (17.9)
Age, median (IQR) years	31 (16–50)	22 (4–43)	38 (22–57)	30 (9–48)	32 (14–54)	20 (3–40)
Age category - *n* (%)^c^						
≤ 1 year	66 (11.8)	100 (11.9)	75 (7.9)	68 (5.7)	107 (13.1)	157 (10.8)
> 1 to ≤5 year	43 (7.7)	159 (18.9)	36 (3.8)	160 (13.5)	60 (7.4)	315 (21.6)
> 5 to <15 years	24 (4.3)	110 (13.1)	44 (4.6)	136 (11.5)	37 (4.6)	197 (13.5)
≥ 15 years	427 (76.2)	472 (56.1)	797 (83.7)	820 (69.3)	610 (74.9)	790 (54.1)
Sex^c^						
Male	270 (48.2)	409 (48.6)	447 (46.9)	579 (48.9)	380 (46.7)	699 (47.9)
Female	290 (52.8)	432 (51.4)	505 (53.1)	605 (51.1)	434 (53.3)	760 (52.1)
Department - *n* (%)^a^						
Intensive Care	50/60 (83.3)	–	86/116 (74.1)	–	89/106 (83.9)	–
Pediatrics	89/107 (83.2)		92/126 (73.0)	–	124/171 (72.5)	–
Obstetrics and Gynecology	97/117 (82.9)	–	159/185 (85.9)	–	111/171 (64.9)	–
Medicine	136/245 (55.5)	–	253/421 (60.1)	–	223/377 (59.2)	–
Surgery	147/168 (87.5)	–	277/300 (92.3)	–	169/186 (90.9)	–
Other	41/60 (68.3)	–	85/117 (72.7)	–	98/134 (73.1)	–
General practice	–	841/3,076 (27.3)	–	1,184/5,597 (21.2)	–	1,459/5,038 (28.9)
Number of antimicrobial(s) - *n* (%)^c^						
One	298 (53.2)	769 (91.4)	508 (53.3)	1,035 (87.4)	484 (59.4)	1,312 (89.9)
Two	234 (41.8)	60 (7.1)	351 (36.9)	126 (10.6)	276 (33.9)	131 (9.0)
≥ Three	28 (5)	12 (1.5)	93 (9.8)	23 (2.0)	54 (6.6)	16 (1.1)
**Number of prescriptions - *n***	**852**	**926**	**1,492**	**1,357**	**1,217**	**1,622**
Route of administration - *n* (%)^d^						
Oral	119 (14.0)	926 (100)	225 (15.1)	1,355 (99.8)	274 (22.5)	1,615 (99.6)
Parenteral	733 (86.0)	0	1,267 (84.9)	2 (0.2)	943 (77.5)	7 (0.4)
Number of antibiotic prescriptions - *n* (%)^d^	842 (98.8)	826 (89.2)	1,446 (96.9)	1,197 (88.2)	1,145 (94.1)	1,503 (92.7)
AWaRe antibiotics - *n* (%)^d^						
Access	423 (50.2)	561 (67.9)	694 (48.0)	743 (62.1)	511 (44.6)	1,030 (68.5)
Watch	416 (49.4)	265 (32.1)	750 (51.9)	454 (37.9)	634 (55.4)	473 (31.5)
Reserve	0	0	0	0	0	0
Not recommended	3 (0.4)	0	2 (0.1)	0	0	0
Indication - *n* (%)^d^						
Treatment of infection	459 (53·9)	757 (82)	795 (53.3)	1,049 (77.1)	717 (58.9)	1,341 (82.7)
Medical prophylaxis-general	50 (5.9)	3 (0.3)	79 (5.3)	2 (0.2)	58 (4.8)	5 (0.3)
Medical prophylaxis-trauma	25 (2.9)	6 (0.7)	29 (1.9)	2 (0.2)	20 (1.6)	2 (0.1)
Medical prophylaxis-vaginal delivery	42 (4.9)	0	72 (4.8)	1 (0.1)	58 (4.8)	0
Single dose surgical prophylaxis	13 (1.5)	0	13 (0.9)	0	12 (1)	0
24-hours-surgical prophylaxis	32 (3.7)	0	11 (0.7)	0	35 (2.9)	0
More than 24 hours-surgical prophylaxis	131 (15.4)	3 (0.3)	307 (20.6)	2 (0.2)	202 (16.6)	1 (0.1)
Unclear indication	18 (2.1)	21 (2.3)	35 (2.3)	35 (2.6)	17 (1·4)	38 (2.3)
Unknown	82 (9.6)	136 (14.7)	151 (10.1)	266 (19.6)	98 (8.0)	235 (14.5)
Indication documented in patient records - *n* (%)^d^	752 (88.3)	769 (83.0)	1,306 (87.5)	1,056 (77.8)	1,102 (90.5)	1,349 (83.2)
Treatment - *n* (%)^d^						
Empirical	844 (99.1)	924 (99.8)	1,460 (97.9)	1,357 (100)	1,147 (94.2)	1,622 (100)
Targeted^e^	8 (0.9)	2 (0.2)	32 (2.1)	0	70 (5.8)	0
Stop/review date documented - *n* (%)^d^	0	855 (92.3)	0	1,339 (98.7)	50 (4.1)	1,445 (89.1)
Focused organ system - *n* (%)^d^						
Bone and Joint	25 (2.9)	22 (2.4)	30 (2.1)	28 (2.1)	33 (2.7)	17 (1.0)
Cardiovascular System	8 (0.9)	3 (0.3)	14 (0.9)	9 (0.7)	8 (0.6)	5 (0.3)
Central Nervous System	39 (4.6)	6 (0.7)	32 (2.1)	4 (0.3)	40 (3.3)	6 (0.4)
Ear-Nose-Throat	34 (4.0)	330 (35.6)	44 (2.9)	318 (23.4)	59 (4.8)	630 (38.8)
Gastrointestinal	195 (22.9)	142 (15.3)	433 (29.0)	271 (20.0)	251 (20.6)	238 (14.7)
Male genital tract	10 (1.2)	9 (1.0)	16 (1.1)	36 (2.6)	10 (0.8)	18 (1.1)
Obstetrics/Gynecology	162 (19.0)	57 (6.2)	311 (20.8)	112 (8.2)	234 (19.2)	70 (4.3)
Ophthalmology	4 (0.5)	1 (0.1)	15 (1.0)	3 (0.2)	1 (0.08)	0
Respiratory tract	140 (16.4)	164 (17.7)	173 (11.6)	156 (11.5)	254 (20.9)	222 (13.7)
Sepsis	64 (7.5)	2 (0.2)	97 (6.5)	1 (0.1)	94 (7.7)	4 (0.2)
Skin and Soft Tissue	59 (6.9)	61 (6.6)	102 (6.8)	146 (10.8)	57 (4.7)	113 (7.0)
Urinary tract	42 (4.9)	60 (6.5)	104 (7.0)	153 (11.3)	77 (6.3)	152 (9.4)
Not defined	70 (8.2)	69 (7.4)	121 (8.1)	120 (8.8)	99 (8.1)	147 (9.1)

**Note:** Paper-based group (PP-G) = group with paper-based version of the antimicrobial prescribing guidelines, App-based group (App-G) = group with application-based version of the antimicrobial prescribing guidelines and antimicrobial stewardship training. ^a^denominator was the total number of screened patient documents, ^b^denominator was the total number of screened patient documents in each hospital, ^c^denominator was number of patients with antimicrobial(s), ^d^denominator was number of prescriptions, ^e^targeted treatment refers to prescribing based on confirmed microbiological results (e.g., conventional culture or Gene-Xpert for tuberculosis), Other (department) = including VIP departments, ENT-EYE (Ear, Nose, Throat and Eyes).

#### Overall percentage of antimicrobial prescriptions fully adherent to prescribing guidelines between PP-G and App-G among hospitalized patients.

Of 3,561 inpatient prescriptions, 3,242 (91.0%; 95% CI [90.1,92.0]) had sufficient information to assess guideline adherence. Among the 319 prescriptions that could not be assessed, 21.9% (70/319) lacked clear prescribing indications such as pleural effusion or wound without information of infection conditions. Of 319 prescriptions, 78.1% (249/319) involved conditions not covered by guidelines. The most common topics not covered by the guidelines were related to gastrointestinal (GI) system conditions (34.5% [86/249]) such as GI bleeding or entero-colitis, followed by central nervous system (17.7% [44/249]) such as brain injury, and other conditions (14.5% [36/249]) such as polytrauma.

**Fig 2:**
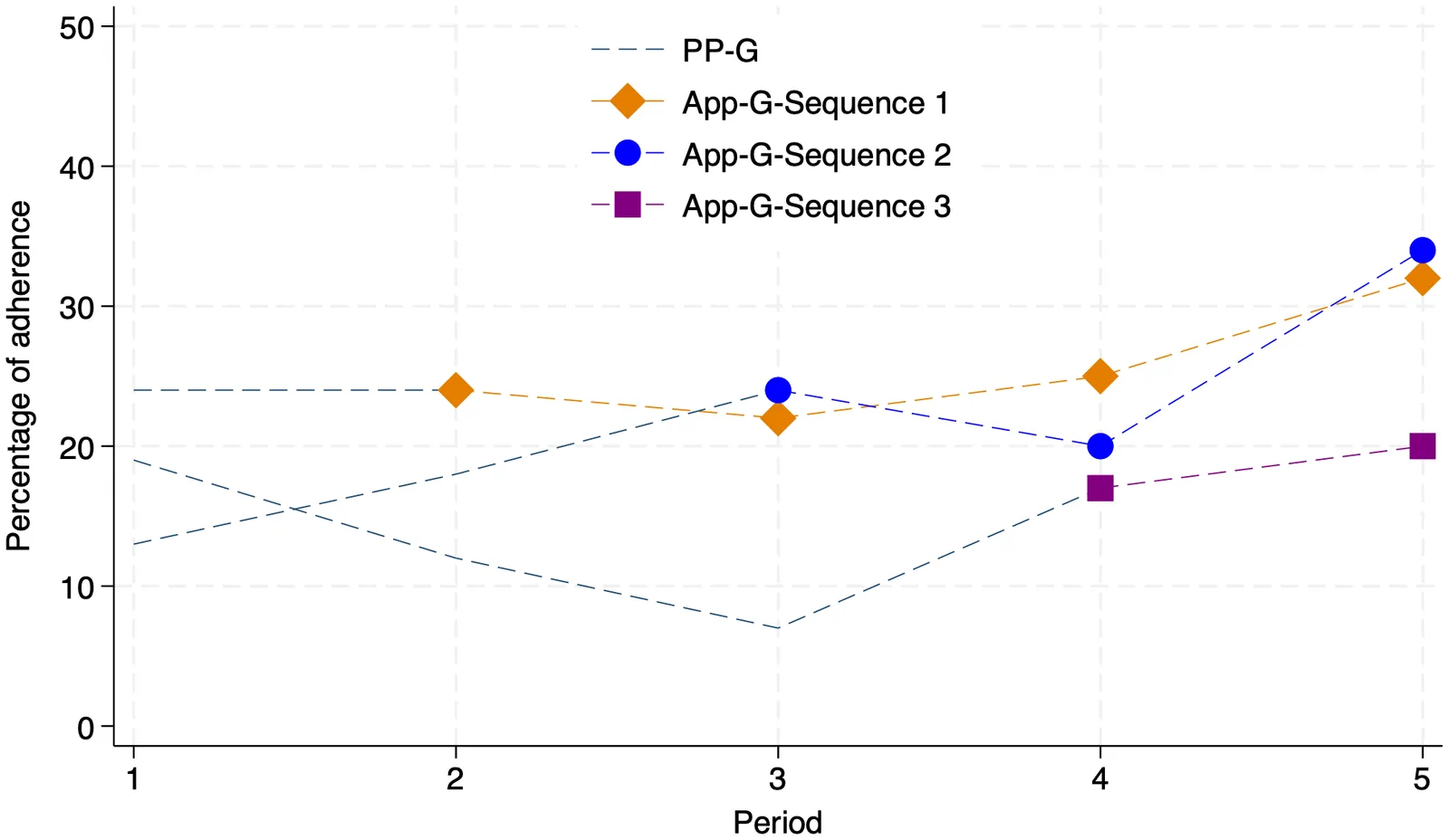
Percentage of antimicrobial prescriptions adherent to prescribing guidelines by sequence of intervention among hospitalized patients in six participating hospitals. **Note**: PP-G = group with paper-based version of the antimicrobial prescribing guidelines; App-G = group with application-based version of the antimicrobial prescribing guidelines and antimicrobial stewardship training; Sequence 1 (Xiengkhuang+Luang Namtha) = started at month-6 after PP-G implementation, Sequence 2 (Salavan+Savannakhet) = started at month-11 after PP-G, Sequence 3 (Mahosot+Vientiane Province) = started at month-17 after PP-G. Points represent observed adherence at each survey period. Connecting lines are included only to facilitate visualization of the stepped-wedge rollout and adherence patterns across sequences. They do not represent continuous measurements between observations.

After all hospitals had access to the paper-based guidelines for ~4–6 months, surveys were conducted across all hospitals to assess adherence. At this time point, adherence to the prescribing guidelines was 17.0% (127/746; 95% CI [14.9,19.9]). By the end of the trial, when all hospitals had access to both guideline formats, overall adherence increased to 28.0% (202/722; 95% CI [24.7,31.4]; *p* < 0.001) (Figs 1 and 2). The percentage of guideline adherence in each hospital varied across the survey periods, ranging from 6.7% (9/134; 95% CI [3.1,12.4] at VTP in period-2% to 36.0% (77/214; 95% CI [39.5,42.8] at SVK in period five (S5 Fig).

#### Primary outcome: Comparison of full adherence to the prescribing guidelines between PP-G and App-G among hospitalized patients.

During the guideline implementation period, adherence in the App-G group (25.6%; 95% CI [23.1,28.3]) had 76% higher odds compared to the PP-G group (17.0%; 95% CI [15.0,19.1]), S5 Fig), with an unadjusted odds ratio (OR) of 1.76 (95% CI [1.1,2.9]; *p* = 0.025). As time is a potential confounder in SW-CRT, to distinguish the effects of these variables on guideline adherence, the model included time, hospitals and patients as random effects (S3 Text). The analysis was conducted at the indication level (*n* = 1,455). Adherence to the guidelines and the intervention were associated with patient age, department, prescribing indications, type of prescription (empiric or targeted [prescribing based on confirmed microbiological results, e.g., conventional culture or Gene-Xpert for tuberculosis]), sequence of intervention implementation and gender. Empiric or targeted prescription was removed from the final model because 97.5% of prescriptions were empiric. In the final model, after adjusting for potential confounders and accounting for random effects, the adjusted odds ratio (aOR) of antimicrobial prescription adherence to guidelines in the App-G group compared to the PP-G group was 1.26 (95% CI [0.8,1.9]; *p* = 0.276) (Table 2). The sequence of intervention introduction was not associated with guideline adherence.

**Table 2:** Antimicrobial prescriptions’ adherence to prescribing guidelines between paper-based and app-based groups among hospitalized patients in six hospitals.

Variables	Unadjusted-OR [95% CI]	*p*-value	Adjusted-OR [95% CI]	*p*-value
Guideline delivery method				
Paper-based (PP-G)	1		1	
App-based (App-G)	1.76 [1.1,2.9]	0.025	1.26 [0.8,1.9]	0.276
Sequence of intervention				
Sequence 3 (MS + VTP)	1		1	
Sequence 2 (SVK + SV)	1.88 [1.1,3.1]	0.013	1.35 [0.8,2.2]	0.220
Sequence 1 (XK + LNT)	2.23 [1.2,4.1]	0.010	1.50 [0.8,2.7]	0.172
Age				
≤ 1 year	1		1	
> 1 to ≤5 years	0.47 [0.2,1.2]	0.107	0.67 [0.3,1.5]	0.322
> 5 to ≤14 years	0.22 [0.1,0.7]	0.008	0.45 [0.2,1.1]	0.081
≥ 15 years	0.26 [0.1,0.5]	<0.001	0.83 [0.4,1.8]	0.626
Gender				
Male	1		1	
Female	0.49 [0.3,0.7]	<0.001	0.74 [0.5,1.0]	0.086
Prescribing indication				
Prophylaxis	1		1	
Treatment	6.65 [3.8,11.5]	<0.001	2.69 [1.6,4.6]	<0.001
Department				
Surgery	1		1	
Obstetrics and Gynecology	0.21 [0.1,0.5]	<0.001	0.41 [0.2,1.0]	0.043
Medicine	3.21,2.0,5.1]	<0.001	2.52 [1.5,4.2]	<0.001
Pediatrics	2.96 [1.7,5.1]	<0.001	2.30 [1.0,5.5]	0.060
Intensive Care Unit	3.09 [1.7,5.5]	<0.001	2.33 [1.1,4.8]	0.022
Other	0.93 [0.5,1.7]	0.821	0.91 [0.5,1.8]	0.780

**Note**: PP-G = group with paper-based version of the antimicrobial prescribing guidelines, App-G = group with application-based version of the antimicrobial prescribing guidelines and antimicrobial stewardship training, Other (department) = including VIP departments, ENT-EYE (Ear, Nose, Throat and Eyes), XK = Xiengkhuang, LNT = Luang Namtha, SV = Salavan, SVK = Savannakhet, VTP = Vientiane Provincial, MS = Mahosot, Sequence 1 (Xiengkhuang+Luang Namtha) = started at month-6 after PP-G implementation, Sequence 2 (Salavan+Savannakhet) = started at month-11 after PP-G, Sequence 3 (Mahosot+Vientiane Province) = started at month-17 after PP-G. *p*-values were derived from the Wald Chi-Square test. The model was adjusted for sequence, age group, sex, prescribing indication, and hospital department. Indication was the unit of analysis, with random effects for patient and hospital-time.

Adherence to guidelines was twice as high in medical wards compared to surgical wards (aOR = 2.52; 95% CI [1.5,4.2]; *p* < 0.001). The odds of adherence in the ICUs were also twice as high as the surgical wards (aOR = 2.33; 95% CI [1.1,4.8]; *p* = 0.022). When compared to antimicrobial prescriptions for prophylaxis, prescriptions for the treatment of infections were nearly three times more likely to adhere to guidelines (aOR = 2.69; 95% CI [1.6,4.6]; *p* < 0.001).

Guideline adherence was low for both surgical and medical prophylaxis at 4.2% (31/743; 95% CI [2.8,5.9]) and 15.7% (48/306; 95% CI [11.8,20.2]), respectively. Among nonadherent surgical prophylaxis prescriptions, 87.6% (624/712; 95% CI [85.0,90.0]) involved incorrect antibiotics. Among incorrect choices, 86.9% (542/624; 95% CI [83.9,89.4]) were also prescribed for an incorrect duration. Of these, 66.5% (415/624; 95% CI [62.6,70.2]) were for elective obstetrics and gynecology (OBGY) surgeries, 13.1% (82/624; 95% CI [10.6,16.0]) for urological, and 9.8% (61/624; 95% CI [7.6,12.4]) for gastrointestinal procedures. In addition to incorrect antibiotic choices for elective surgical prophylaxis, 11.8% (84/712; 95% CI [9.5,14.4]) used correct antibiotics and dose but incorrect duration; and 0.6% (4/712; 95% CI [0.1–1.4]) used correct antibiotics but with incorrect dose and duration. In most of these cases, antimicrobials were continued for more than 24 hours post-operatively. For medical prophylaxis, 62.8% (162/258; 95% CI [56.6,68.7]) of nonadherent prescriptions were associated with uncomplicated vaginal deliveries.

Among pneumonia cases, the most common prescribing indication, after adjusting for patient age and accounting for the stepped-wedge design, the adjusted-OR for guideline adherence in the App-G group compared to the PP-G group was 1.61 (95% CI [0.6,4.0]; *p* = 0.300). Prescriptions for adults were less likely to adhere to guidelines than those for infants (≤1 year), with 95% lower odds of adherence (aOR = 0.05; 95% CI [0.02,0.1]; *p* < 0.001).

When adherence was assessed based on antimicrobial agent selection alone, overall adherence in the App-G group was 34.6% (385/1,112; 95% CI [31.8,37.5]) and 30.6% (416/1,360; 95% CI [28.1,33.1]) in the PP-G group. There was no evidence of a difference between groups, with an aOR of 1.02 (95% CI [0.7,1.4]; *p =* 0.915) for App-G compared with PP-G.

#### Secondary outcomes among hospitalized patients.

Focusing on the proportion of antimicrobial prescriptions (*n* = 3,167 inpatient records), after adjusting for hospital departments, sequence of intervention implementation, and accounting for the stepped-wedge design, the odds of adherence in the App-G group compared to the PP-G group were 0.82 (95% CI [0.59,1.13]; *p* = 0.224). Nonsurgical wards had substantially lower antimicrobial prescription percentages than surgical wards (S7 Table).

The overall DDD per 100 patient-days among adult inpatients was 105.3. DDD varied between hospitals and over time. In the pre-guidelines group, DDD was 103.4 per 100 patient-days, compared to 113.0 in the PP-G group and 97.3 in the App-G group (S8 Table). The most prescribed antibiotic among adult inpatients was the Watch antibiotic, ceftriaxone which contributed 49.3 DDD per 100 patient-days in the pre-guidelines group, 50.5 DDD in the PP-G group, and 41.4 DDD in the App-G group.

Intravenous antimicrobial use among hospitalized patients accounted for 2,943/3,561 (82.6%; 95% CI [81.4,83.9]) prescriptions. In the pre-guidelines group, 733/852 (86.0%; 95% CI [83.5,88.3]) received IV antimicrobials. IV antimicrobial use was 1,267/1,492 (84.9%; 95% CI [83.0,86.7]) in the PP-G group, and 943/1,217 (77.5%; 95% CI [75.0,79.8]) in the App-G group. After adjusting for sequence of intervention introduction, patient’s age, and department, the odds of IV antimicrobial use in the App-G group compared to the PP-G group were 0.77 (95% CI [0.5,1.0]; *p* = 0.103) (S9 Table).

### Outpatient settings

Out of 13,711 OPD patients, 3,484 (25.4%; 95% CI [24.7,26.2]) received antimicrobials. This included 27.3% (841/3,076; 95% CI [25.8,28.9]) in the pre-guideline group, 21.1% (1,184/5,597; 95% CI [20.1,22.2]) in the PP-G group, and 28.9% (1,459/5,038; 95% CI [27.7,30.2]) in the App-G group. Most patients (59.7% [2,082/3,484]) were aged ≥15 years, and almost all OPD patients (89.4% [3,116/3,484]) received a single antimicrobial (Table 1). Cefalexin was the most frequently prescribed agent, followed by amoxicillin and azithromycin. Overall, 66.2% (2,334/3,905) of antibiotics belonged to WHO AWaRe Access category (S5 and S10 Tables). The use of Access antibiotics was above 70% in children (84.5% [268/317]; 95% CI [80.1,88.3]) in infants aged ≤1year, 78.7% (462/587; 95% CI [75.2,81.9]) in those >1–≤5years, 76.6% (291/380; 95% CI [72.0,80.7]) in those >5–<15 years) and 58.6% (1,313/2,242; 95% CI [56.5,60.6]) in adults. These patterns remained similar across intervention and control groups (S6 Fig). Tonsillopharyngitis was the most common prescribing indication, accounting for 29.6% (1,159/3,905) of all antimicrobial prescriptions. It accounted for 20.0% (272/1,357; 95% CI [17.9,22.2]) in the control group and 36.2% (588/1,622; 95% CI [33.9,38.6]) in the intervention group (S7 Fig).

#### Overall percentage of antimicrobial prescriptions fully adherent to prescribing guidelines between PP-G and App-G among outpatients.

**Fig 3:**
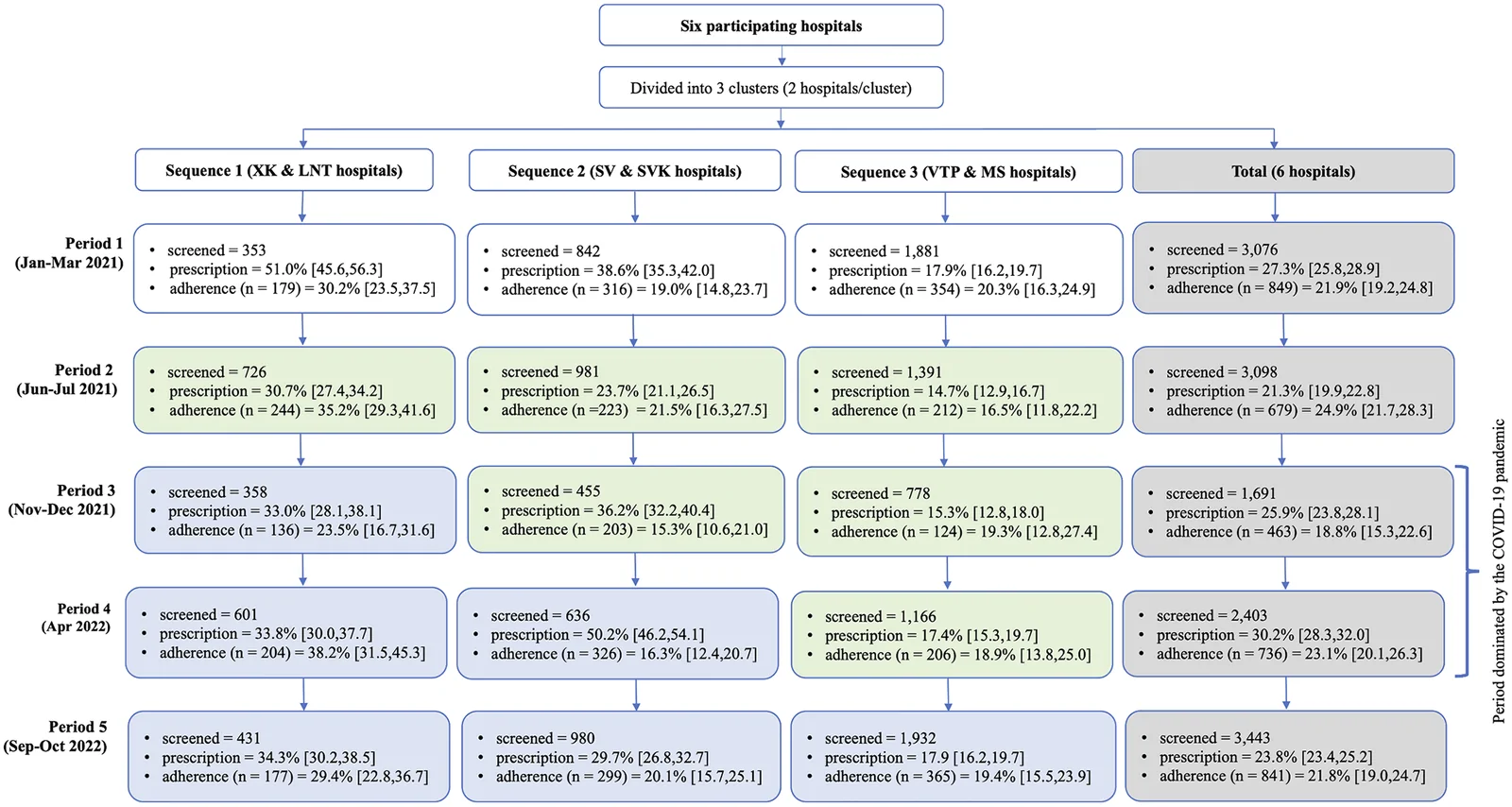
Lao AntiMicrobial Prescribing Application (LAMPA) trial profile for outpatients. **Note**: White area (*n* = 926 prescriptions) = group with no antimicrobial prescribing guidelines, green area (*n* = 1,357 prescriptions) = group with paper-based version of prescribing guidelines (PP-G), blue area (*n* = 1,622 prescriptions) = group with application-based version of prescribing guidelines and antimicrobial stewardship training (App-G); XK = Xiengkhuang; LNT = Luang Namtha; SV = Salavan; SVK = Savannakhet; VTP = Vientiane Province; MS = Mahosot; screened = Number of screened patient’s documents; prescription = Percentage of patients prescribed an antimicrobial [95% CI]; adherence = Percentage of antimicrobial prescriptions fully adherent to the guidelines [95% CI]; *n* = Number of prescriptions with sufficient information to assess guideline adherence; Period 1 = Pre-guideline, Period 2 = Month-6 after PP-G implementation (all hospitals), Period 3 = First survey after App-G implementation in Xiengkhuang and Luang Namtha or month-11 after PP-G introduction, Period 4 = Second survey post-App-G for Xiengkhuang and Luang Namtha, first survey post-App-G for Salavan and Savannakhet, or month-17 after PP-G introduction, Period 5 = final survey after all clusters had App-G access or month-22 after PP-G introduction.

Of 3,905 OPD antimicrobial prescriptions, 3,568 (91.4%; 95% CI [90.5,92.2]) could be assessed for guideline adherence. Adherence was comparable between the PP-G and App-G groups, at 21.7% (263/1,212; 95% CI [19.4,24.1]) and 23.0% (346/1,507; 95% CI [20.9,25.2]), respectively. After all hospitals had used the paper-based guidelines for ~6 months, overall adherence was 24.9% (169/679; 95% CI [21.6,28.1]) (Fig 3). Variation was observed across hospitals. By the end of the trial, when all hospitals had access to both guideline formats, the overall adherence across hospitals was 21.8% (183/841; 95% CI [19.0,24.7]) (S8 Fig).

#### Primary outcome: Comparison of full adherence to the prescribing guidelines between PP-G and App-G among outpatients.

**Fig 4:**
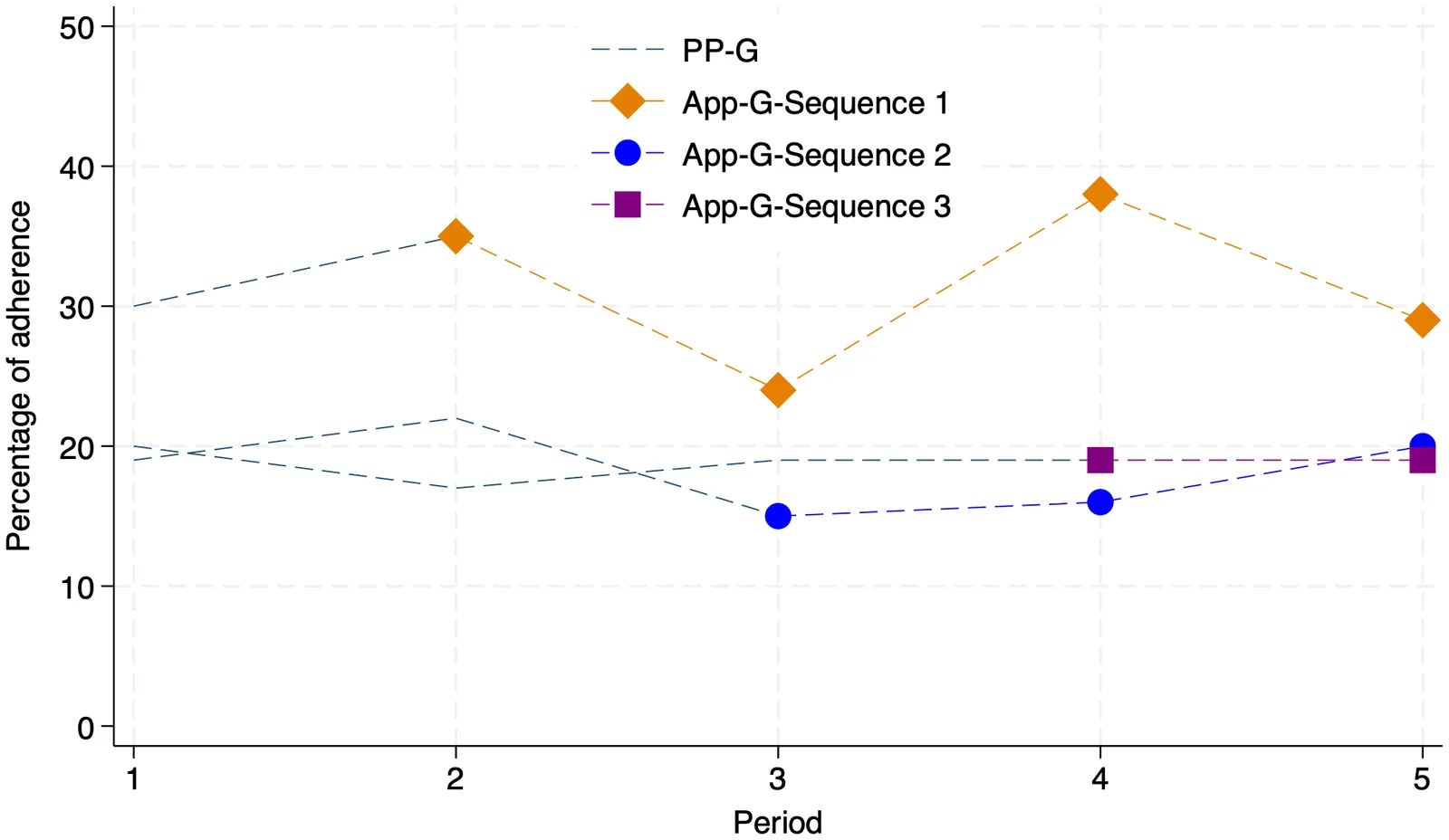
Percentage of antimicrobial prescriptions adherent to prescribing guidelines by sequence of intervention among outpatients in six participating hospitals. **Note**: PP-G = group with paper-based version of the antimicrobial prescribing guidelines; App-G = group with application-based version of the antimicrobial prescribing guidelines and antimicrobial stewardship training; Sequence 1 (Xiengkhuang+Luang Namtha) = started at month-6 after PP-G implementation, Sequence 2 (Salavan+Savannakhet) = started at month-11 after PP-G, Sequence 3 (Mahosot+Vientiane Province) = started at month-17 after PP-G. Points represent observed adherence at each survey period. Connecting lines are included only to facilitate visualization of the stepped-wedge rollout and adherence patterns across sequences. They do not represent continuous measurements between observations.

Among 2,477 indications, guideline adherence in the PP-G group was similar to the App-G group, with unadjusted-OR= 1.23 (95% CI [0.8,1.8]; *p* = 0.305) and aOR = 0.91 (95% CI [0.7,1.1]; *p* = 0.406), after adjusting for patient age, hospital group (sequence of intervention introduction), and accounting for time, patient and cluster random effects. Adherence varied by hospital group, with the first group exposed to the App-G showing higher odds of adherence (32.1% [302/940]; 95% CI [29.1,35.2]) compared with the last group (19.1% [241/1,261]; 95% CI [16.9,21.4]) with an aOR of 1.95 (95% CI [1.4,2.7]; *p* < 0.001), whereas no difference was observed between the second and last groups (Fig 4 and Table 3).

**Table 3:** Antimicrobial prescriptions’ adherence to prescribing guidelines between paper-based and app-based groups among outpatients in six hospitals.

Characteristics	Unadjusted-OR [95% CI]	*p*-value	Adjusted-OR [95% CI]	*p*-value
Guideline delivery method				
PP-G	1		1	
App-G	1.23 [0.8,1.8]	0.305	0.91 [0.7,1.1]	0.406
Sequence of intervention				
Sequence 3 (MS + VTP)	1			
Sequence 2 (SVK + SV)	0.91 [0.7,1.2]	0.466	0.82 [0.6,1.1]	0.140
Sequence 1 (XK + LNT)	2.1 [1.5,2.9]	<0.001	1.95 [1.4,2.7]	<0.001
Age				
≤ 1 year	1		1	
> 1 to ≤5 years	1.30 [0.8,2.0]	0.220	1.27 [0.8,1.9]	0.267
> 5 to ≤14 years	1.60 [1.0,2.5]	0.041	1.58 [1.0,2.5]	0.047
≥ 15 years	0.80 [0.5,1.2]	0.269	0.78 [0.5,1.2]	0.225
Gender				
Male	1			
Female	0.99 [0.8,1.2]	0.956	–	–
Prescribing indication				
Prophylaxis	1			
Treatment	0.91 [0.1,5.2]	0.918	–	–

**Note**: PP-G = group with paper-based version of the antimicrobial prescribing guidelines, App-G = group with application-based version of the antimicrobial prescribing guidelines and antimicrobial stewardship training, Other (department) = including VIP departments, ENT-EYE (Ear, Nose, Throat and Eyes), XK = Xiengkhuang, LNT = Luang Namtha, SV = Salavan, SVK = Savannakhet, VTP = Vientiane Provincial, MS = Mahosot, Sequence 1 (Xiengkhuang+Luang Namtha) = started at month-6 after PP-G implementation, Sequence 2 (Salavan+Savannakhet) = started at month-11 after PP-G, Sequence 3 (Mahosot+Vientiane Province) = started at month-17 after PP-G. *p*-values were derived from the Wald Chi-Square test. The model was adjusted for sequence and age group. Indication was the unit of analysis, with random effects for patient and hospital-time.

Tonsillopharyngitis was the most common indication. No difference in guideline adherence was observed between the App-G and PP-G groups (aOR = 1.45; 95% CI [0.8,2.7]; *p* = 0.245). Adults had 67% lower odds of adherence compared with infants (aOR = 0.33; 95% CI [0.2,0.6]; *p* = 0.001).

Using antimicrobial agent selection as the criterion for adherence, 43.5% of prescriptions in the App-G group were adherent (656/1,507; 95% CI [43.0,46.1]), compared with 42.2% in the PP-G group (511/1,212; 95% CI [39.4,45.0]). The aOR for guideline adherence was 0.82 (95% CI [0.7,1.0]; *p =* 0.062) for App-G relative to PP-G.

#### Secondary outcomes among outpatients.

Overall antimicrobial prescribing among outpatients did not differ between the PP-G group (21.2%; 95% CI [20.1,22.3]) and the App-G group (29.0%; 95% CI [27.8,30.2]), after adjusting for intervention sequence and random effects, odds of prescriptions in the App-G group compared to the PP-G group was 1.28 (95% CI [0.72,2.29]; *p* = 0.411). Among adult outpatients, antibiotic use in DDDs is presented in S11 Table. Intravenous antibiotic use in outpatients was not assessed, as only nine prescriptions (0.2%) of the 3,905 OPD prescriptions were given intravenously.

### Patterns of App usage

Cumulative app data showed peak access between 8–10 AM and around 1–2 pm, highest at 9 am, aligning with typical consultation hours. Over 22 months, the app was accessed 6,239 times, or 24.8 times/prescriber (6,239/251 prescribers who reported using the app), or ~1/month. The cumulative number of app searches during the study period was 527, and the most frequently searched topic was melioidosis, followed by pneumonia and antimicrobial agents (S9 and S10 Figs). Searches reflect use of the search function, while direct navigation was counted as access.

## Discussion

Antimicrobial prescribing guidelines are considered to be fundamental to good antimicrobial prescribing practice; however, there is limited evidence comparing the impact of different delivery formats, particularly paper-based and app-based guidelines on adherence. In this study, we document the development and implementation of local antimicrobial prescribing guidelines in Laos. This SW-CRT compared adherence to newly released local antimicrobial prescribing guidelines delivered in both paper-based and mobile app formats.

Full adherence to antimicrobial prescribing guidelines was 17.5% (95% CI [14.9,20.4]) in the pre-guideline group, 17.0% (95% CI [15.0,19.1]) in the PP-G group, and 25.6% (95% CI [23.1,28.3]) in the App-G group. These adherence percentages were lower than those reported in other LMICs (31.9% in Myanmar [28], 52.2% in Indonesia [29], 54.5% in Uganda [30], 69.2% in Vietnam [31], 77.4% in India [32], 84% in Tanzania [33], 85.8% in HICs like North America [34], and 83.6% in Canada [35]). Most of these studies classified guideline adherence based only on the correct choice of antimicrobial agents or it was not clear how guideline adherence was defined. Full adherence to prescribing guidelines in this study was classified as correct antimicrobial agent and dose based on provided national antimicrobial prescribing guidelines, except for surgical prophylaxis which included duration of prophylaxis. It is essential to establish standardized criteria and definitions for adherence to prescribing guidelines to ensure data comparability.

The primary outcome of this study is that the odds of guideline adherence were 26% higher in the App-G than in the PP-G group but statistical significance was not observed (aOR = 1.26; 95% CI [0.8,1.9]; *p* = 0.276). It is possible that the study was underpowered to detect a difference, due to the number of participating hospitals. Although this increase in adherence was not substantial, it suggests that structured easily accessible antimicrobial use guidelines may support prescribing. However, the relatively infrequent app use indicates that it was not used consistently as a routine clinical support tool in this setting. We did not observe an association between guideline adherence and the sequence of intervention implementation. Our results highlight the need for continued efforts to implement and strengthen adherence to the guidelines which could be targeted in departments where nonadherence is highest. Previous studies have reported improvements in adherence to antimicrobial prescribing guidelines after implementing smartphone applications. Most of these studies converted existing prescribing guidelines in their settings into a smartphone application format [10,11,13,16,17], and focused on specific infectious diseases, for example: pyelonephritis [9], diarrhoeal disease [11], community-acquired pneumonia (CAP) and urinary tract infections (UTI) [16,17], or specific situations such as pediatric patients [13,14] or surgery procedures [12]. A similar study in HICs found no difference in appropriate antibiotic use between the mobile app and standard care groups, mainly because prescribers forgot to use the app or relied on other available tools [36]. While the pocketbook is the traditional way to share guidelines, the mobile app offers a more modern, innovative approach, aligned with the move towards digital solutions in healthcare. In Laos, when new treatment guidelines become available, it is difficult to ensure that all hospital prescribers have access. Typically, only a few copies are provided per ward, which prescribers must share. In this study, the paper-based and app-based guidelines were distributed to all prescribers in participating hospitals which may have increased adherence in the paper-based group compared to usual practice. However, even with full availability of new guidelines and training in their use, adherence remained low. Some studies have described that guidelines uptake could be influenced by the acceptance of the prescribers and how they perceived the guidelines [37,38]. In-depth interviews with 16 prescribers across two hospitals involved in this SW-CRT revealed that both the paper-based and app-based guidelines were generally well accepted by Lao prescribers. However, many interviewees admitted they rarely referred to the guidelines, either because they had memorized the recommendations or because they relied on habitual prescribing practices [39]. This suggests that prescribing decisions are driven not only by guideline availability but also by established clinical routines and contextual factors within routine practice. Lack of access to diagnostics for many infections or AMR also impedes antimicrobial stewardship. These findings suggest that, in this setting, prescribing behavior may be shaped by established clinical habits and broader contextual factors, highlighting the potential need for additional strategies to support effective antimicrobial stewardship.

Low adherence to guidelines in the surgical and obstetrics and gynecology departments contributed to the overall low adherence observed in this study. A possible explanation for this could be concerns about surgical site infections related to perceptions about the cleanliness of the hospital environment, which may be associated with extended durations of antimicrobial prophylaxis or the unnecessary combination of antibiotics for broader coverage, even for routine procedures, such as uncomplicated vaginal deliveries, where prophylaxis is not required. Possible strategies to address this issue include improving clinical knowledge and training, monitoring prescribing practices and giving feedback, and implementing decision support systems for prescribers. Further research is needed to better understand the factors associated with this prescribing behavior, to inform context-appropriate interventions these groups.

In our outpatient setting, guideline adherence did not differ between the PP-G and App-G groups (aOR = 0.91; 95% CI [0.7,1.1]; *p* = 0.406). Inappropriate antimicrobial use among outpatients is widely reported. Ardrillon and colleagues (2023) found that 76.5% (95% CI [75.1,77.9]) of outpatients in three LMICs received antibiotics unnecessarily [40]. A national outpatient survey from China (2017–2019) similarly reported inappropriate prescribing in 70.5% (147,758/209,662; 95% CI [70.3,70.7]) of consultations [41]. High levels of inappropriate use have also been documented for common conditions such as UTIs, pneumonia, and upper respiratory infections. For example, among patients with UTI in the USA (2013–2018), only 47% of prescriptions were appropriate (2,762/5,870; 95% CI [45.8,48.3]) [42]. In British Columbia, 44% of 42,452 prescriptions for pediatric outpatients with CAP (2014–2018) were inappropriate [43]. A cluster RCT by Rambaud-Althaus and colleagues (2017) compared a clinical algorithm for childhood illness delivered via paper-based and mobile application formats in outpatient departments. Both formats were associated with lower antibiotic prescribing compared with standard practice (paper: 26%, OR= 0.4, 95% CI [0.2,0.6]; app: 25%, OR= 0.3, 95% CI [0.2,0.5], both *p* < 0.001), with no difference between formats (OR= 1.1; 95% CI [0.6,1.6]; *p* = 0.825). Guideline adherence was not assessed [14]. In this study, upper respiratory infections were the most common prescribing indication in outpatients with 98.5% classified as tonsillopharyngitis. Cefalexin and azithromycin were frequently prescribed across sites and periods despite not being recommended in the guidelines, with high levels of inappropriate antimicrobial use observed in OPD settings. These findings highlight a need for strengthened outpatient antimicrobial stewardship activities in Laos.

The COVID-19 pandemic may have been associated with low adherence in this study. The intervention was implemented during the peak of COVID-19 in Laos. This period was characterized by changes in hospital operations, including the relocation of many doctors to COVID-19 centers, which may have limited their exposure to the App-G and left them unavailable for training sessions. Hospital patient numbers also decreased, with as few as eight patients recorded in a single survey in Luang Namtha. Additionally, some wards were designated for COVID-19 case management. This unexpected situation may have influenced prescribing patterns and guidelines uptake. However, data from routine PPS conducted after the trial have shown similar patterns, suggesting that COVID-19 was unlikely to be the main determinant of the observed findings. Additionally, the app and AMS training were delivered once per hospital, which may have limited familiarity and confidence in using the app. Repeated trainings on an annual basis could be considered to support sustained use.

In some regions of Laos, healthcare providers may have limited digital literacy, or there may be a reluctance to adopt new technology due to unfamiliarity or complexity. In our study, all participating prescribers had access to smartphones, and the application was designed to function offline; therefore, internet connectivity limitations were unlikely to be a major barrier in this setting. However, differences in confidence and experience with mobile applications may have been associated with variations in uptake. This digital divide may be reflected in lower utilization of app-based guidelines, with some prescribers continuing to use the traditional paper-based version rather than switching to the app-based version. However, paper-based guidelines also have limitations, including challenges in nationwide dissemination, delays in updating content, and costs associated with printing and distribution, especially when compared to app-based guidelines. In many healthcare settings, long-standing habits have been reported to influence prescribing behaviors. These established practices may be difficult to change, even in the presence of new guidelines that are locally appropriate. High workload has also been described as being associated with challenges in guideline adherence. Lack of feedback and reinforcement by senior staff have also been reported in previous studies [39]. To maintain the study’s pragmatic study design, we did not have active contact with prescribers unless they had questions about using the guidelines. Accordingly, encouragement and reinforcement of guidelines use were not systematically applied across all study sites. In this context, observed changes in guideline adherence may be limited in settings where such mechanisms were not consistently present.

Improving guideline adherence is challenging, and maintaining adherence over time may present additional difficulties. Given the limited number of studies on sustainability of guideline adherence following the implementation of smartphone applications [10,13,17], it is difficult to fully understand the factors that may be related to these changes or to draw firm conclusions on approaches to support adherence. To improve understanding of these processes, further mixed-methods research with extended monitoring may be useful.

There are several limitations to this trial. First, we did not measure individual prescriber behavior changes after the intervention implementation. Data were collected from patients’ medical records, from which it was not possible to reliably attribute prescriptions to individual prescribers. Second, although the study was adequately powered based on available data at the time, the inclusion of only six participating hospitals may have affected the precision of subgroup analyses of guideline adherence in some specific infectious diseases or conditions, due to the relatively small number of cases. In addition, variation in cluster sizes meant that some clusters contributed fewer observations, which may have affected the precision of estimates of differences between groups. Third, the PPS method was used to measure outcomes at each time point. While it provides a useful snapshot of antimicrobial use, it does not capture the full picture of antimicrobial use in the study hospitals over the entire study period. Fourth, this trial was conducted during the COVID-19 pandemic. This was associated with delays in intervention implementation and outcome measurements.

This study suggests that technology-based delivery of antimicrobial prescribing guidelines is feasible in a low-resource setting, such as Laos, and may be relevant to ongoing efforts to support healthcare quality. However, overall guideline adherence remained low, and there was insufficient evidence that using a smartphone application to deliver prescribing guidelines was associated with differences in adherence compared to the paper-based guidelines. These findings suggest that guidelines alone are insufficient to support appropriate antimicrobial use. Addressing broader, multifaceted barriers may be important, including improving guideline awareness, access to appropriate technology and skills, and strengthening healthcare services in the context of human resource constraints. Regular feedback and sustained support from senior hospital leadership and the Ministry of Health may also be relevant to support consistent guideline use and improve patient care.

## Supporting information

S1 Fig Stepped-wedge cluster randomized controlled trial study sites in Laos. Note: Mahosot Hospital is located in Vientiane Capital, while Vientiane Provincial Hospital is in Vientiane Province. The base layer data for the country and provincial boundaries were obtained from the GADM website: https://gadm.org/. The license information for academic use is available at: https://gadm.org/license.html. The map was created by the research team using QGIS.
(TIFF)

S2 Fig 10 most prescribed antimicrobial agents, by ward. Note: Labels from top to bottom correspond to columns from left to right, ordered from highest to lowest values.
(TIFF)

S3 Fig WHO AWaRe group use by age category among hospitalized patients in the paper-based and app-based guideline group. Note: PP-G = group with paper-based version of the antimicrobial prescribing guidelines, App-G = group with application-based version of the antimicrobial prescribing guidelines and antimicrobial stewardship training.
(TIF)

S4 Fig Antimicrobial prescriptions by selected prescribing indications among hospitalized patients. Note: Labels from top to bottom correspond to columns from left to right, ordered from highest to lowest values; CNS = Central Nervous System.
(TIFF)

S5 Fig Percentage of antimicrobial prescriptions fully adherent to prescribing guidelines among hospitalized patients, % (95% CI). Note: White area = group with no antimicrobial prescribing guidelines, green area = group with paper-based version of prescribing guidelines (PP-G), blue area = group with application-based version of prescribing guidelines and antimicrobial stewardship training (App-G), Period 1 = Pre-guideline, Period 2 = Month-6 after PP-G implementation (all hospitals), Period 3 = first survey after App-G implementation in Xiengkhuang and Luang Namtha or month-11 after PP-G introduction, Period 4 = second survey post-App-G for Xiengkuang and Luang Namtha, first survey post-App-G for Salavan and Savannakhet, or month-17 after PP-G introduction, Period 5 = final survey after all clusters had App-G access or month-22 after PP-G introduction, (a) = Guideline adherence by groups (Pre-guideline, PP-G and App-G), (b) = Guideline adherence by hospital in each survey.
(TIFF)

S6 Fig WHO AWaRe group use by age category among outpatients in the paper-based and app-based guideline group. Note: PP-G = group with paper-based version of the antimicrobial prescribing guidelines, App-G = group with application-based version of the antimicrobial prescribing guidelines and antimicrobial stewardship training.
(TIFF)

S7 Fig Five most common prescribing indications among outpatients, by age group. Note: Labels from top to bottom correspond to columns from left to right, ordered from highest to lowest values; Unknown = without a written diagnosis or with a diagnosis unrelated to infection treatment or prophylaxis; STI = sexually transmitted infection.
(TIFF)

S8 Fig Percentage of antimicrobial prescriptions fully adherent to prescribing guidelines among outpatients, % (95% CI). Note: White area = group with no antimicrobial prescribing guidelines, green area = group with paper-based version of prescribing guidelines (PP-G), blue area = group with application-based version of prescribing guidelines and antimicrobial stewardship training (App-G), Period 1 = Pre-guideline, Period 2 = Month-6 after PP-G implementation (all hospitals), Period 3 = first survey after App-G implementation in Xiengkhuang and Luang Namtha or month-11 after PP-G introduction, Period 4 = second survey post-App-G for Xiengkuang and Luang Namtha, first survey post-App-G for Salavan and Savannakhet, or month-17 after PP-G introduction, Period 5 = final survey after all clusters had App-G access or month-22 after PP-G introduction, (a) = Guideline adherence by groups (Pre-guideline, PP-G and App-G), (b) = Guideline adherence by hospital in each survey.
(TIFF)

S9 Fig Frequency of antimicrobial prescribing guideline access by time of day (data from MicroGuide). (TIFF)

S10 Fig Guideline search patterns among prescribers (Data from MicroGuide). Note: MRSA = Methicillin-resistant *Staphylococcus aureus*; PCP = Pneumocystis pneumonia; ESBL = Extended-Spectrum Beta-Lactamase; UTI = Urinary Tract Infection.
(TIF)

S1 Table Stepped-wedge cluster randomized controlled trial (SW-CRCT) study sites. (DOCX)

S2 Table Total number of prescribers with access to paper-based and app-based antimicrobial prescribing guidelines. (DOCX)

S3 Table Specialty distribution of prescribers with access to prescribing guidelines included in the study. (DOCX)

S4 Table Number of screened hospitalized patient (IPD) records and outpatient (OPD) prescriptions in participating hospitals. (DOCX)

S5 Table List of antimicrobial prescriptions in six hospitals in each survey period. (DOCX)

S6 Table Antimicrobial prescriptions categorized by WHO AWaRe (Access, Watch, Reserve) group among hospitalized patients. (DOCX)

S7 Table Comparison of antimicrobial use between app-based and paper-based guidelines groups among hospitalized patients in six hospitals. (DOCX)

S8 Table Overall antibiotic defined daily doses (DDD) per 100 patient days in adult hospitalized patients in six hospitals before and after antimicrobial prescribing guidelines implementation. (DOCX)

S9 Table Comparison of the use of intravenous antimicrobials between PP-G and App-G groups among hospitalized patients. (DOCX)

S10 Table Antimicrobial prescriptions categorized by WHO AWaRe (Access, Watch, Reserve) group among outpatients. (DOCX)

S11 Table Overall antibiotic defined daily dose (DDD) among adult outpatients in six hospitals before and after antimicrobial prescribing guidelines implementation. (DOCX)

S1 Text LAMPA (Lao Anti-Microbial Prescribing App) Study Proposal. (PDF)

S2 Text Stata code for sample size calculation and statistical analysis. Note: Codes are also available at https://doi.org/10.6084/m9.figshare.32051097.
(DOCX)

S3 Text Statistical Analysis Plan (SAP). Note: SAP is also available at https://doi.org/10.6084/m9.figshare.32016120.
(DOCX)

S1 Checklist CONSORT checklist. Hopewell S, Chan AW, Collins GS, Hróbjartsson A, Moher D, Schulz KF, and colleagues. CONSORT 2025 Statement: updated guideline for reporting randomized trials. BMJ. 2025; 388:e081123. https://dx.doi.org/10.1136/bmj-2024-081123. © 2025 Hopewell and colleagues. This is an Open Access article distributed under the terms of the Creative Commons Attribution License (https://creativecommons.org/licenses/by/4.0/), which permits unrestricted use, distribution, and reproduction in any medium, provided the original work is properly cited.
(DOCX)

S2 Checklist Inclusivity in global research. (DOCX)

## Abbreviations

AMR: antimicrobial resistance
AMS: antimicrobial stewardship
aOR: adjusted odds ratio
CAP: community-acquired pneumonia
CI: confidence interval
CONSORT: consolidated standards of reporting trials
DDDs: defined daily doses
GI: gastrointestinal
HICs: high-income countries
LAMPA: Lao AntiMicrobial Prescribing Application
LMICs: low-and middle-income countries
OR: Odds ratios
PPS: point prevalence surveys
SW-CRT: stepped-wedge cluster randomized controlled trial
TrACSS: Tracking AMR Country Self-Assessment Survey
UTI: urinary tract infections.
