# Supplementary material for: Adherence to smartphone-delivered antimicrobial prescribing guidelines in hospitals in Lao PDR: A stepped-wedge cluster randomized controlled trial
Source: PLoS Med. 2026 Aug 27;23(8):e1005205. doi: 10.1371/journal.pmed.1005205 (PMC13521649; doi:10.1371/journal.pmed.1005205)
Supplement: Supplementary file 1 [file pmed.1005205.s022.pdf]

Evaluating the impact of a Lao language mobile phone antimicrobial use guideline application on antimicrobial prescribing in Laos

Short title: LAMPA (Lao Anti-Microbial Prescribing App)

Principal Investigator: Vilada Chansamouth

Co-investigators

Elizabeth Ashley
Paul Newton
Paul Turner
Rogier van Doorn
Mayfong Mayxay
Nicholas Day
Yoel Lubell
Pimnara Peerawaranun
Mavuto Mukaka
Manivanh Vongsouvath
Andrew Simpson
Dr Sommai Keomany
Dr Khamla Silavanh
Dr Nunthakone Sihapanya
Dr Phouvieng Duangmala
Dr Khamkeo Mivapadith
Dr Khamsai Dethleuxay
Dr Valy Keoluangkhot
Dr Sommana Rattana

Funder: Wellcome Trust

Study sponsor: University of Oxford

Table of Contents

TRIAL SYNOPSIS 4

I. INTRODUCTION 7

ANTIMICROBIAL RESISTANCE - GLOBAL SITUATION 7
COUNTRY PROFILE OF LAOS 8
HEALTHCARE SYSTEM IN LAOS 8
ANTIMICROBIAL RESISTANCE IN LAOS 10
HOSPITAL ANTIMICROBIAL USE IN LAOS 10
THE NEED FOR ANTIMICROBIAL PRESCRIBING GUIDELINES 12
STUDY RATIONALE 13

II. STUDY OBJECTIVES AND HYPOTHESIS 13

STUDY OBJECTIVES 13
Primary objective 13
Secondary objectives 13

III. METHODS 14

3.1 STUDY DESIGN 14
3.2 STUDY SITES 14
3.3 SAMPLE SIZE AND POWER CALCULATION 16
3.4 RANDOMIZATION AND BLINDING 17
3.5 STUDY INTERVENTION AND REFERENCE 17
a. Pre-study phase 18
Lao antimicrobial prescribing guidelines 18
b. Reference phase 19
c. Intervention phase 20
3.6 ENDPOINTS 23
Primary endpoint 23
Secondary endpoints 23
Definitions of adherence to the guidelines 24
3.7 DATA COLLECTION METHOD 24
a. Point Prevalence Survey procedures 26
a.3 Antimicrobial stewardship (AMS) knowledge survey 31
Pre-prescribing guidelines survey 32
Prescriber feedback survey on paper-based prescribing guidelines 32
Prescriber feedback survey on mobile phone App prescribing guidelines 33
DATA MANAGEMENT AND MONITORING 33
STATISTICAL ANALYSIS 33
SAFETY CONSIDERATIONS/ADVERSE EVENTS 34
ETHICS 35
BENEFITS 35
RISKS 35
PATIENT AND DOCTOR CONFIDENTIALITY 36
TRIAL INSURANCE 36
DATA SHARING AND DISSEMINATION 36
FINANCE 37

REFERENCES 38

APPENDICES 42

A1. IN-PATIENT PPS WARD FORM 42

A2. In-patient PPS Patient Form 43
A3. Outpatient PPS Form 44
A4. Point Prevalence Survey (PPS) of hospital antimicrobial use Report Format 45
A5. Antimicrobial stewardship (AMS) Knowledge survey 51
A6. Introductory session of therapeutic and prophylactic antimicrobial prescribing guidelines 55
A7. Introductory session of antimicrobial prescribing guidelines mobile phone application (MicroGuide) 58
A8. Introductory (paper/App guidelines) session registration sheet 59
A9. Pre-prescribing guideline Survey 60
A10. Prescriber feedback survey on paper-based prescribing guidelines 61
A11. Prescriber feedback survey on mobile phone app prescribing guidelines 63
A12. Antimicrobial stewardship (AMS) training 65
A13. Antimicrobial stewardship (AMS) training session registration sheet 66
A14. Timelines and milestones 67

Trial synopsis

**Primary Objective**	To compare the proportion of antimicrobial prescriptions adherent with prescribing guidelines delivered by mobile phone application (app) versus paper-based prescribing guidelines in both in- and outpatients in six general hospitals in Laos
**Secondary objectives**	1. To compare the change in antimicrobial defined daily doses (DDDs) after introduction of paper-based versus mobile phone app prescribing guidelines plus antimicrobial stewardship (AMS) training in both in- and out patients<br>2. To compare the change in proportion of patients receiving antimicrobials by the intravenous route between paper-based and mobile phone app prescribing guidelines + AMS training in both in- and out patients<br>3. To compare the AMS knowledge level of prescribers before and after prescribing guidelines delivered by mobile phone app and AMS training<br>4. To compare the prescriber satisfaction with/acceptability of paper-based prescribing guidelines versus mobile phone app prescribing guidelines<br>5. To compare antimicrobial costs before and after prescribing guidelines delivered by mobile phone app and AMS training in both in- and outpatient departments<br>6. To compare the proportion of patients admitted to hospital receiving an antimicrobial prescription before and after introduction of the paper-based prescribing guidelines; and the mobile phone app prescribing guidelines + AMS training
**Methodology**	
Study design	An open cohort stepped-wedge cluster randomized controlled trial 3-step trial (4-month intervals at each step) with a four-month pre-intervention period)
Study sites	Six general hospitals in Laos (two in the north (Luang Namtha and Xiengkhuang Provincial Hospitals), two in the centre (Mahosot Hospital and Vientiane Provincial Hospital) and two in the south (Savannakhet and Salavan Provincial Hospitals)
Study duration	16 months (four months preintervention period and 4-month interval between each step (three steps))
Sample size and power calculation	The sample size of six hospitals was estimated assuming 30% antimicrobial prescription adherence with the paper-based prescribing guidelines (reference), and assuming 0.05 intra-cluster correlation coefficient. This will have 80% power to be able to detect a 15% minimum difference in adherence after introduction of the mobile phone app prescribing guidelines (intervention) at a 0.05 significance level.
Randomization and blinding	Simple random sampling will be used to select the order (step) of interventions implementation for a group of hospitals (two hospitals per group and one group per step). Neither hospitals nor investigators will be blinded to the timing of intervention.
Intervention	A MicroGuide antimicrobial prescribing guideline mobile phone application and

	AMS training
Reference	Paper-based antimicrobial prescribing guidelines
Outcomes/Primary endpoint	The proportion of antimicrobial prescriptions adherent with prescribing guidelines delivered by mobile phone app versus paper-based prescribing guidelines at month 16 (12 months' exposure in each group)
Secondary endpoints	1. Total antimicrobial defined daily doses (DDDs) prescribed in mobile phone app versus paper-based groups after 12 months' exposure in each group<br>2. Proportion of patients prescribed antimicrobials by the oral route in mobile phone app versus paper-based groups after 12 months' exposure in each group<br>3. AMS knowledge level of prescribers prescribed in mobile phone app versus paper-based groups after 12 months' exposure in each group<br>4. Prescriber satisfaction/acceptability of the prescribing guidelines in mobile phone app versus paper-based groups after 4 months' exposure in each group<br>5. Total costs of antimicrobials prescribed in mobile phone app versus paper-based groups after 12 months' exposure in each group<br>6. Proportion of patients admitted to hospital receiving an antimicrobial prescription before and after introduction of the paper-based prescribing guidelines; and the mobile phone app prescribing guidelines + AMS training after 12 months' exposure in each group
Outcome measurement	• Point prevalence surveys (PPS) of hospital antimicrobial use<br>• Prescriber questionnaires<br>• AMS knowledge surveys of prescribers
Data management	Open Data Kit (ODK) system will be used as data collection instrument. We will collect data on patients' characteristics, antimicrobial prescription status, the frequency of use of the antimicrobial prescribing guidelines (both paper-based and mobile app), AMS knowledge level of prescribers, acceptability of the guidelines and the cost of antimicrobial prescriptions in both groups.<br><br>All data will be kept securely, protected by password access with automatic daily backup.
Statistical analysis	A logistic mixed-effects regression model will be used to compare the proportion of antimicrobial prescription adherence measured cross-sectionally at month 0, 4, 8, 12 and month 16 after introduction of prescribing guidelines delivered by mobile phone application versus paper-based. The model will compare all wedges in the intervention from month 4 to month 16 with all wedges in the reference arm from month 0 to month 12. The duration of exposure of each hospital to the reference or intervention arm will be included in the model as interaction term with the study arm in order to assess whether the duration of exposure is the effect modifier. The model will consider the correlation among the outcomes' measurements from the same hospital (cluster) by adjusting for clustering in the model. Odds ratios and the corresponding confidence intervals will be obtained and reported.
Ethics	University of Health Sciences, Vientiane, Lao PDR and Oxford Tropical Ethics Committee (OxTREC), University of Oxford, United Kingdom

Trial insurance	Provided by University of Oxford
Dissemination	1/ Quarterly and annual reports (PPS dashboard and paper report) to participating hospitals, Lao Ministry of Health<br><br>2/ Publication in national and international journals<br><br>3/Oral presentation/poster in both national and international levels
Sponsor	University of Oxford
Funder	Wellcome Trust of Great Britain

I. Introduction

Antimicrobial resistance (AMR) is the ability of microorganisms, including bacteria, fungi, viruses and parasites, to withstand exposure to antimicrobial medicines, such as antibacterial, antifungal, antiviral, anti-protozoal and anti-helminthic, so that the treatment becomes ineffective (1). Resistance can be acquired or intrinsic. Intrinsic resistance is the innate ability of bacteria to withstand the activity of some antimicrobial compounds whilst acquired resistance is the ability of bacteria to resist a particular antimicrobial compound which it was originally susceptible to. In a clinical setting, treating infections caused by bacteria having acquired novel resistance mechanisms is the main problem of AMR (2).

Antimicrobial Resistance - global situation

AMR is a major global public health concern with rising frequencies of AMR in key pathogens to key antimicrobials. The evidence suggests that this increase is accelerated by use, misuse or overuse of antimicrobials (3). AMR results in both direct consequences, such as longer duration of illness, increased mortality and prolonged stay in hospital, and indirect consequences such as reduced productivity caused by sickness and increased family, community and health system economic burden (4). O'Neill estimated that ~700,000 people die every year from drug resistant pathogens, including bacterial infections, HIV and malaria. If AMR continues to increase with no appropriate action being taken, this could contribute 10 million excess deaths and a cost of 100 trillion USD worldwide by 2050 (5). In 2019, Limmathurotsakul *et al.* argued that the estimation of the AMR global burden may have been distorted because of limitations in the methodological approaches (6). Since 2000, the number of antibiotics in development has fallen sharply. Many pharmaceutical companies have left the antibiotics market, stopping production and engagement in research and development (R&D), as the returns in profit of drugs given as short courses and with a short therapeutic life are far lower than those from drugs intended for lifelong use such as anti-hypertensive, or cholesterol-lowering drugs that do not fall to resistance (7), With drug-resistant infections potentially causing global economic damage on a par with the 2008 financial crisis, low-income countries could lose more than 5% of their GDP. AMR could push up to 28 million people into poverty, mostly in developing countries. Healthcare costs may increase by $300 billion to more than $1 trillion per year. A decline in global livestock production could range from 2.6% to 7.5% per year (8).

In order to address this major threat to global public health, there have been a number of

international initiatives. The Global Action Plan (GAP) to combat AMR was established by WHO in 2015, which consists of five strategies: 1) improving awareness and understanding of antimicrobial resistance; 2) strengthening knowledge through surveillance and research; 3) reducing the incidence of infection; 4) optimizing the use of antimicrobial agents; and 5) ensuring sustainable investment in countering antimicrobial resistance. The GAP mainly focuses on antibiotic resistance and provides a framework for National Action Plans to combat antimicrobial resistance over the next 5 to 10 years (9). In the same year, the global antimicrobial resistance surveillance system (GLASS) was established to support GAP. GLASS focuses on some specific microorganisms in specific samples such as: *Escherichia coli, Klebsiella pneumoniae, Acinetobacter baumannii, Staphylococcus aureus, Streptococcus pneumoniae* and *Salmonella* spp. in blood; *Escherichia coli* and *Klebsiella pneumoniae* in urine; *Salmonella* spp. and *Shigella* spp. in faeces; and *Neisseria gonorrhoeae* from urethral and cervical swabs (10). In February 2017, three global priority tiers of antibiotic-resistant bacteria were proposed by WHO to guide research, discovery and development of new antibiotics, including critical, high and medium priority pathogens (11).

Country profile of Laos

Lao PDR (Laos) is a land-linked country in Southeast Asia and bordered by China, Myanmar, Thailand, Cambodia and Vietnam. The majority (63%) of the population of ~6.77 million people live in rural areas and are predominantly dependent on farming. In 2013 the Gross National Income per capita was US$ 4,570/year and life expectancy was 66 years. The World Bank reclassified Laos as a lower middle-income country in 2013 (9). From 2005 to 2015 Laos had one of the fastest growing economies in the world, with an average GDP growth rate of 7.8% per annum (12). Total health expenditure per capita increased from US$29 in 2010-2011 to $59 in 2015-2016 but total health expenditure as a proportion of GDP ranged from 2.4% to 3% during 2010 and 2016 (13). This is low compared to surrounding countries where the GDP expenditure is 6.5% in Thailand, 5.7% in Cambodia, and 7% in Vietnam, but is similar to that of Myanmar (2.3% of GDP) (12).

Healthcare system in Laos

The governmental healthcare system in Laos comprises three main administrative levels, including the Ministry of Health (MoH), Provincial Health Offices and District Health Offices. Based on data from the Curative and Healthcare Department in 2017, there are five central hospitals in Vientiane City and 17 provincial hospitals, 135 district hospitals (29 with ability of performing surgery and 106 unable to perform surgery) and 1,020 health centers (866 with ability to perform normal

vaginal delivery and 154 unable to perform normal vaginal delivery), country-wide (Curative and Healthcare Department, unpublished data 2017). There were 0.8-hospital beds per 1,000 population in 2010-2011. The most common issues found in the service delivery system are overcrowding of patients at central and provincial levels and underuse of district and community-level facilities (14). There are also a number of private international medical facilities in Vientiane, with links to Vietnam, China, France and the United Nations. There are also Thai government and private hospitals within easy reach of Vientiane and other towns in the Mekong Valley in Thailand. An Infection Prevention Control (IPC) committee was formed by MoH in 2008 and guidance on IPC at central and provincial hospitals was released in the same year. In 2014, IPC guidance for all healthcare levels was released. There are IPC committees in all central and provincial hospitals in Laos, as well as in some district hospitals.

In 2016, there were 20,484 health workers for the whole country (6,758,000 population) or 3/1,000 population. There were 2,716 physicians or 0.4/1,000 and 913 pharmacists or 0.13/1,000 population, and one medical school and one-degree level pharmacy programme within the University of Health Sciences in Vientiane (Ministry of Health, unpublished data 2016).

Limitations of laboratory services and clinical research infrastructure and capacity challenge the development of effective and evidence-based clinical and public health management strategies. In rural Laos, most treatment of infections is empirical, with basic laboratory testing such as Gram staining available at some provincial hospitals (15).

The national laboratory network structure in Laos is in line with the overall five-tiered organization of the national health system. Of these five laboratories, only two laboratories (National Center for Laboratory and Epidemiology (NCLE) and Microbiology Laboratory/Lao-Oxford-Mahosot Hospital Wellcome Trust Research Unit (LOMWRU) routinely culture bacteria and perform antimicrobial susceptibility testing. Some laboratories in central (Sethathirad Hospital and Mittaphab Hospital) and provincial hospitals are able to culture and identify some bacteria. The National TB Laboratory and Centre d'Infectiologie Christophe Mérieux of Laos (CICML) perform GeneXpert TB assays (16). Until mid-2019, both Mahosot Hospital and NCLE laboratories used disk diffusion according to Clinical & laboratory Standards Institute (CLSI) guidelines for most of their antibiotic susceptibility testing and before changing to European Committee on Antimicrobial Susceptibility Testing (EUCAST).

Antimicrobial resistance in Laos

Awareness of AMR is relatively recent in Laos and it is becoming a major public health concern. The main current concerns are Extended Spectrum Beta-Lactamase (ESBL) producing Enterobacteriaceae. ESBL producing *K. pneumoniae* and *E. coli* were first identified from the Mahosot Hospital Laboratory in clinical samples in 2000 and 2004, respectively. The frequency of ESBL-producing *E. coli* bacteraemia during 2004-2016 was 135/693 (19%), but the proportion of isolates producing ESBL increased 5-fold from 2004 2/28 (7%) to 2016 – 27/78 (35%). Whilst the proportion of *K. pneumoniae* producing ESBL was 39/284 (14%) between 2000-2016 in patients at Mahosot Hospital, there was no clear trend for an increase in ESBL producing *K. pneumoniae*, with levels varying from 3% to 41% over this period (Microbiology Laboratory/LOMWRU, unpublished data). Carbapenem resistance in *E. coli* was first identified in Laos in 2015 from a pus specimen from a patient admitted to Mahosot Hospital. Since then there have been three further isolates with the first carbapenem resistant *E. coli* isolated from blood in 2016 and 2017. All four isolates carried a New Delhi metallo-β-lactamase (NDM) gene (17). An analysis of 18 years of *Salmonella* Typhi bacteraemia data (2000-2018) in Laos showed 79% (712/898) were susceptible to all tested antimicrobials, 6.5% were multidrug-resistant and only 1% was fluoroquinolone resistant (18). Methicillin-resistant *Staphylococcus aureus* (MRSA) was also infrequent but it requires close monitoring; 18 years of bacteraemia data from Mahosot Hospital (2000-2018) only showed 2 patients with invasive MRSA infection. However, of concern, during 2017 and 2018, 71/426 (17%) *Staphylococcus aureus* isolates cultured from pus from clinical specimens at Mahosot Hospital were MRSA (Microbiology Laboratory/LOMWRU, unpublished data). There is scattered information on other GLASS pathogens available in Laos.

Hospital antimicrobial use in Laos

The Lao Food and Drug Department (FDD) is responsible for drug registration, licensing of pharmacies/drug companies/drug industries, and overseeing their distribution, import, drug quality control, domestic manufacture and also monitoring the supply chain. In 2016, 218 antibiotic active pharmaceutical ingredients (APIs) and combination APIs were registered by the Lao FDD; of these 38 (17%) were produced in Laos. The main sources of packaged antibiotics are from neighbouring countries such as Thailand and China (http://www.fdd.gov.la/showContent.php?contID=48).

In 2006, Keohavong *et al*. described that nearly half (47%) of Lao outpatients within 30 health

facilities investigated throughout the entire country were prescribed antibiotics for mild diseases such as simple diarrhoea or common colds (19). Few data on hospital antimicrobial use exist in Laos; at one northern provincial hospital, 51% of 1,095 patients (2008-2010) received antibiotics but only 7% of these received appropriate antibiotics (20). Nearly 70% of Lao doctors prescribed antibiotics at least once daily and stated that they had not received much information on appropriate antibiotic use during their careers. The doctors stated that they mainly prescribed antibiotics according to the National Standard Treatment Guidelines, advice from their peers, more experienced colleagues, pharmaceutical companies or the Internet. Doctors indicated a preference for local antibiotic guidelines written in Lao language to international ones in other languages (21).

The first hospital antibiotic use survey in Laos was conducted in 2017 using the Global Point Prevalence Survey methodology (LOMWRU, unpublished data). In 2018, five general hospitals across Laos participated in this survey, including two from the north, two from the centre and one from the south of Laos. Of 1,981 in-patient hospitals chart screened, 72% documented an antimicrobial (s) prescription, 43% patients received more than one antimicrobial, 98% were prescribed empirically and nearly half of them received antimicrobials for prophylaxis (LOMWRU, unpublished data). These hospital antimicrobial prescribing rates were much higher than rates (47%) reported in 2006 by Keohavong *et al.* (2006) (19).

Keohavong *et al.* (2019) interviewed 54 healthcare providers at outpatient departments (OPD) in five health facilities in Savannakhet Province, Laos, finding that 98% prescribed antibiotics based on patients' symptoms. The majority of them (91%) had heard about antimicrobial resistance but only 46.3% had been trained in the rational use of drugs. Medical records of 576 OPD patients under 5 years-old with upper respiratory tract infection (URTI) or common cold were screened for antibiotic prescription, 96% of URTI patients received antibiotic (s) and 5% for common cold patients. Of 405 patients given antibiotics, 395 (97.5%) received first line antibiotics (amoxicillin, ampicillin, erythromycin and penicillin V) based on Lao National Standard Treatment Guidelines. However, only a few of them used correct doses and durations based on these Guidelines (22).

There is evidence that multi-activity antimicrobial stewardship (AMS) programs could significantly reduce the proportion of therapeutic and prophylactic antibiotic prescriptions in both in- and outpatients from a study of a large patient population in China during 2010 and 2016 (23). Scheetz *et al*. (2009) also showed that combining antimicrobial stewardship with standard treatment of bloodstream infections had favourable cost-effectiveness compared to many other standard treatments of care (24). AMS programs have only been recently established in Laos. The first

training session on AMS to improve the rational use of antibiotic of Lao doctors, will be held in Vientiane City by Ministry of Health in mid-2020 with some pilot hospitals.

The need for antimicrobial prescribing guidelines

Lao Standard Treatment Guidelines (STG) were first established in 1998 and the latest edition was published in 2012. The guidelines are broad and cover a variety of issues such as medicines, surgery, and emergency medicines for both adults and children but do not include AMS or guidelines for treatment of many common infections. There are no approved Lao evidence-based infectious disease treatment guidelines, except for some specific diseases, e.g. malaria, TB, Sexually Transmitted Diseases (STDs) and HIV. LOMWRU conducted a small survey on the need for antimicrobial prescribing guidelines in Laos during November and December 2019; 70 Lao doctors from either central or provincial hospitals reported the main challenges to making decisions on the choice of antimicrobials were lack of access to a microbiology laboratory 31/70 (44%), no suitable antimicrobial prescribing guidelines 30/70 (43%) and poor availability of some antimicrobial agents 30/70 (43%). The majority of doctors used Lao treatment guidelines 58/70 (83%) and guidelines from neighboring countries 40/70 (57%) with a small proportion using European or US treatment guidelines 16/70 (23%). Only 17/70 (24%) had used any antimicrobial prescribing guideline/treatment guidelines delivered by a mobile application. Of these, 41% used the Medscape App (LOMWRU, unpublished data).

Antimicrobial prescribing guidelines have now been developed in country and are being adapted with the Ministry of Health based on AMR data from Laos, existing guidelines and international antimicrobial prescribing/treatment guidelines (e.g. WHO, CDC, NICE, Infectious Diseases Society of America (IDSA), Sanford). No antimicrobial use applications (apps) for the Lao PDR are available in English or Lao. Some 90% of Lao doctors from three provincial hospitals have smartphones (unpublished data) and apps are likely to be more attractive to physicians than bulky handbooks. Antimicrobial prescribing guidelines delivered by mobile phone app are now widespread in the world. Some evidence has suggested that antibiotic guidelines on a mobile phone app increased guideline adherence by prescribers, reduced unnecessary antibiotic use in hospital, reduced poor outcomes, improved quality of care and reduced antimicrobial consumption cost (25-27). Panesar *et al.* (2016) reported a survey on the use of MicroGuide smartphone application compared to pocket antimicrobial prescribing guidelines during a 3-month period at the University College London Hospital NHS Trust. Of 116 responses from pre-app survey questionnaires and 112 responses from

post-app survey questionnaires, the prescribing app was used more than pocket prescribing guides ($p<0.01$). The majority of responses (>90%) agreed that the app was easy to navigate and it was the most appropriate means to access hospital guidelines. The app also improved their antimicrobial stewardship awareness (RR 6.8 (CI 2.1-21.7) (28). A study in three main hospitals of Imperial College Healthcare Trust, London demonstrated that when introducing mobile health to the setting with an existing antimicrobial stewardship program, the proportion of antimicrobial prescriptions adherence with the prescribing policy was significantly increased but there was no significant effect on antimicrobial prescription trends (29).

Study rationale

Although the severity of the AMR situation in Laos remains lower than elsewhere in Asia, misuse of antimicrobials is a major driver of resistance. This is a therefore an important opportunity for implementation of a simple technology, which may help reduce further worsening of the AMR situation. Antimicrobial prescribing apps are a promising tool to improve prescribing. However, there has been no evidence from trials to support whether the antimicrobial prescribing guidelines in the format of a mobile phone application are superior to conventional means (i.e. paper–based) in low- and middle-income countries (LMIC). This trial could have a significant impact in Laos and in other LMICs, and inform policy globally, by improving prescribing and hence reducing AMR.

II. Study objectives and hypothesis

Study objectives

Primary objective

To compare the proportion of antimicrobial prescriptions adherent with prescribing guidelines delivered by mobile phone application (app) versus paper-based prescribing guidelines in both in- and outpatients in six general hospitals in Laos

Secondary objectives

1. To compare the change in antimicrobial defined daily doses (DDDs) after introduction of paper-based versus mobile phone app prescribing guidelines plus antimicrobial stewardship (AMS) training in both in- and out patients

2. To compare the change in proportion of patients receiving intravenous antimicrobials route between paper-based and mobile phone app prescribing guidelines + AMS training in both in- and out patients
3. To compare the AMS knowledge level of the prescribers before and after prescribing guidelines delivered by mobile phone app and AMS training
4. To compare the prescriber satisfaction with/acceptability of paper-based prescribing guidelines versus mobile phone app prescribing guidelines
5. To compare antimicrobial costs before and after prescribing guidelines delivered by mobile phone app and AMS training in both in- and outpatient departments
6. To compare the proportion of patients admitted to hospital receiving an antimicrobial prescription before and after introduction of the paper-based prescribing guidelines; and the mobile phone app prescribing guidelines + AMS training

III. Methods

3.1 Study design

The study design will be an open cohort stepped wedge cluster randomized controlled trial (SW-CRT) in six central/provincial hospitals across Laos. SW-CRT is appropriate in the Lao context because it requires fewer sites than a parallel cluster trial, but has similar observational periods for reference and intervention phases. As the interventions will be delivered by one team travelling to each cluster in turn, implementing in stages will be more practical. In addition, all participating hospitals will have access to the novel interventions at the end of the study. Prescribers are allowed to move in and out of clusters during the trial.

3.2 Study sites

Six hospitals (two referral (200-450 beds) and four provincial hospitals (60-100 beds) have been purposively selected based on their capacity, location, previous participation in our PPS program with available hospital antimicrobial use and antimicrobial susceptibility data.

Mahosot Hospital (MS) has 450 beds and is a central referral hospital situated in Vientiane City in Laos (17°95'N 102° 61'E, 268m above mean sea level). There are 968 staff, including 222 physicians,

414 nurses, 19 dentists, 46 pharmacists, 11 radiologists and 147 non-health worker staff. There are 18 in-patient wards and one main out-patient and small number of OPDs within in-patient wards.

Vientiane Provincial Hospital (VTP) has 100 beds. There are 211 staff, including 30 physicians, 99 nurses, 6 dentists and 9 pharmacists (data from 2018). There are six in-patients wards and one outpatient ward. The hospital is ~70km from Vientiane City (18°47’N 102° 48’E 497m above mean sea level).

Xiengkhuang Provincial Hospital (XK) has 150 beds. There are 305 staff, including 66 physicians, 117 nurses, 6 dentists and 24 pharmacists (data from 2019). There are 11 in-patient wards and one main out-patient units and a small number of OPDs within in-patient wards. It is in a broad valley in the northeast highlands (19° 27’N 103° 10’E, 1,150m above mean sea level).

Luang Namtha Provincial Hospital (LNT) has 50 beds. There are 117 staff, including 18 physicians, 36 nurses, 3 dentists and 10 pharmacists (data from 2018). There are 6 in-patients wards and OPDs within in-patient wards. It is in the highlands on the China and Myanmar borders in the far NW of Laos (21° 00’N 101° 24’ E; 570m above mean sea level)

Savannakhet Provincial Hospital (SVK) has 200 beds. There are 293 staff, including 60 physicians, 139 nurses, 7 dentists and 13 pharmacists (data from 2019). There are 9 in-patient wards and one main out-patient and small number of OPDs within in-patient wards. It is located in the southern part of the country in the largest province in Laos (16° 55’N 104° 74’E; 726 m above mean sea level)

Salavan Provincial Hospital (SV) has 70 beds. There are 191 staff, including 30 physicians, 50 nurses, 3 dentists and 11 pharmacists (data from 2018). There are 7 in-patients wards and OPDs within in-patient wards outpatient. It is on the western slopes of Annamite mountains in southern Laos (15°43’N 106°25’ E; 184m above mean sea level).

Figure 1. SW-CRCT study sites

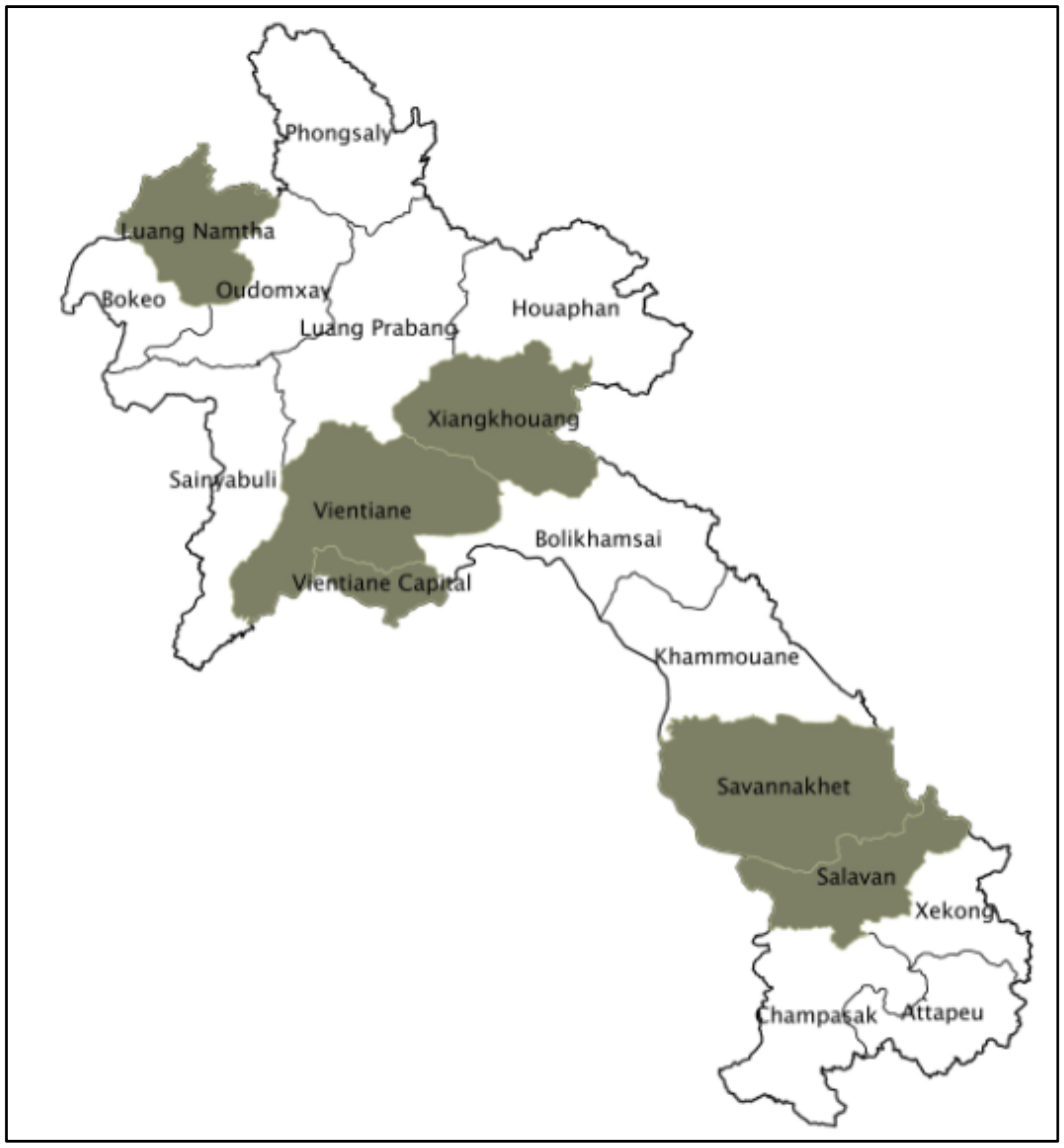

3.3 Sample size and power calculation

A PPS of hospital antimicrobial use in four general hospitals in Laos in 2017 showed 13% compliance to Lao treatment guidelines (LOMWRU unpublished data) but previous guidelines did not contain much information on antibiotic prescribing, therefore reliable baseline data are lacking.

The sample size of 6 hospitals was estimated assuming 30% of antimicrobial prescription adherence with the paper-based prescribing guideline (reference), and assuming 0.05 intra-cluster correlation coefficient. This will have 80% of power of the test to be able to detect 15% minimum difference after introduction of the mobile phone app prescribing guidelines at 0.05 significant level.

This will be a 3-step trial with one baseline measurement after introduction of the paper-based prescribing guidelines. The outcomes will be measured every four months. These will give four outcomes measurements in total (excluding baseline measurement at start), until the trial

completes at month 16.

3.4 Randomization and blinding

Six participating hospitals have been divided into 3 pairs based on the geographical location and convenience for conducting the surveys. The first paired hospitals include two provincial hospitals from the north of Laos (Luang Namtha and Xiengkhuang), the second pair includes hospitals in the center (Mahosot Hospital-central referral hospital and Vientiane provincial hospital which is 80 km from the center of the capital to the north). The third pair includes two provincial hospitals from the south of Laos (Savannakhet and Salavan).

As there are only 3 orders to be assigned, clusters will be randomly assigned to one of three start dates using a simple random sampling, to have access to the study intervention. Three paired hospitals (LNT+XK, MS+VTP and SVK+SV) will be written on three different paper slips (similar paper size and paper texture). All slips will be folded to the same size and put in the non-transparent container, covered firmly and mixed well. A person who is not related to the study will be asked to pick up the paper slip one by one and read out to the PI and a co-investigator. Simple random sampling processes will be performed before the start of the project and the order of each paired hospital to intervention introduction will be recorded. At month 4 after the start of the project, the intervention will be introduced to the first selected paired hospital, then every four months each pair of hospitals will be rolled out from the reference phase to the intervention phase until all have started the intervention. This will give 1:1 ratio of intervention *vs* reference period of observation (figure 2). We will therefore be able to compare the effects of the reference and intervention over a reasonable period of time. Neither hospitals nor investigators will be blinded to the introduction of the intervention.

3.5 Study intervention and reference

Before starting the project, PI, co-investigators and research team will visit each participating hospital to discuss about the project's objectives and how we would like to conduct this project during 16 months to the directors of the hospitals. These six hospitals have already participated in Global PPS, led by the PI and research team of this project. Hospital staff will be informed about the study by the PI and a director of hospital (also co-investigator) before starting the project and all prescribers in participating hospitals will be encouraged to participate in the training and surveys.

a. Pre-study phase

Four participating hospitals (MS, XK, LNT and SV) in this study have already participated in Global-PPS since 2017, whilst VTP participated in 2018 and SVK in 2019. Antimicrobial prescription data of all six hospitals are available for the whole of 2019. This is the period when there were no available Lao therapeutic and prophylactic antimicrobial prescribing guidelines. These data will be used in this project to compare the change in proportion of antimicrobial prescriptions and the change in administration route before and after the introduction of the prescribing guidelines (both paper-based and mobile phone app versions). To be able to answer our study question, Lao therapeutic and prophylactic antimicrobial prescribing guidelines were developed to be introduced at month 0 of the project. Details of Lao therapeutic and prophylactic antimicrobial prescribing guidelines are described below.

Lao antimicrobial prescribing guidelines

Lao National antimicrobial prescribing guidelines are based on existing Lao treatment guidelines, current data on infectious disease pathogens and antimicrobial susceptibility patterns in Laos and international antimicrobial prescribing/treatment guidelines (e.g. WHO, CDC, NICE, ISID, Sanford). The guidelines are separated into paediatric and adult guidelines for ease of use. These guidelines include recommendation for therapy, prevention and antibiotic stewardship.

Most recommendations are for empiric treatment. For pathogens and syndromes for which Lao data are available, common causative pathogens and their antimicrobial susceptibilities are provided as a guide. This guideline also refers to some specific Lao treatment guidelines (e.g. Dengue guideline for diagnosis and treatment, Lao PDR or National Guidelines for the Use of Antiretroviral Therapy in Adults and Children) to avoid duplication.

Common and important infectious diseases are arranged by involved organ systems. Each infectious disease section includes causative pathogen (s) of the diseases, choices of antimicrobial regimen (primary and alternative), doses, duration and important notes on allergy or toxicity or to help guide treatment.

b. Reference phase

Study duration will be divided into two parts, reference and intervention phases. At the start (month 0), a point prevalence survey (PPS) on hospital antimicrobial use will be performed (more details on conduct of PPS are given in following section) to collect baseline data before introducing paper-based guidelines. The participating hospitals will receive a report of the PPS findings within one month of the survey (Appendix A4).

After PPS, all medical doctors of each participating hospital will be asked to complete an AMS knowledge survey (Appendix A5) and pre-prescribing guidelines survey (Appendix A9). Then, paper-based prescribing guidelines in both adult and paediatric versions (one adult guideline and one paediatric guideline) will be distributed to all doctors at participating hospitals, followed by guideline introductory session of the guidelines (Appendix A6). This session will not cover how to diagnose infectious diseases or prescribe antimicrobials but how to use these guidelines.

After introduction of the paper-based guidelines, all six hospitals will be formally included in the reference period. PI and research team will not become involved with any antimicrobial prescribing decisions of local prescribers at participating hospitals for individual patients. There will be contact details of the PI in the guidelines in case any prescribers need more clarification on the guidelines. A prescriber feedback survey on the paper-based version will be performed just before each paired hospital enters to the intervention phase (month 4, month 8 and month 12 after introduction of the paper-based prescribing guidelines).

After the first paired hospital is randomly selected to have access to the mobile phone app prescribing guidelines at month 4, the second pair of hospitals will be randomly selected to have to access to the mobile phone app at month 8, and the last pair of hospitals will have access to a mobile phone app at month 12 (Figure 2).

The order of activities at the start of the project are listed below (these activities will be applied to all six hospitals, one by one):

1. PPS on hospital antimicrobial use for all 6 hospitals (Appendix A1, A2 and A3)
2. AMS knowledge survey (Appendix A5)
3. Pre-prescribing guidelines survey (Appendix A9)
4. Access to paper prescribing guidelines for all 6 hospitals
5. Introductory session to paper prescribing guidelines (Appendix A6)
6. PPS reports (Appendix A4)

Activities in the reference phase after the start of the project (month 0) until the last paired hospital is assigned to the intervention (month 12) are listed below:

1. PPS on hospital antimicrobial use (Appendix A1, A2 and A3)
2. PPS reports (Appendix A4)

c. Intervention phase

c.1 Antimicrobial Prescribing guidelines mobile phone application (MicroGuide)

Both paediatric and adult antimicrobial prescribing guidelines will be transferred to MicroGuide; which is a medical guideline application. MicroGuide was launched in 2013 and has been adopted by 114 medical organizations, with more than 100,000 users in 173 countries. MicroGuide is an offline application, free-of-charge for the individual users. The app can monitor the number of users and which guidelines are being accessed.

The app will be installed to the personal smart phones of local physicians by the PI. The project will not provide a smart phone to any physician without one (approximately <10% of physicians in provincial hospitals– unpublished data). The contents of the mobile app guidelines will be the same as the paper version but using hyperlinks to information, rather than displaying it as in paper guidelines. Both mobile app and paper guidelines will be available in Lao and English languages.

c.2 Antimicrobial stewardship (AMS) training

The aim of AMS training is to increase knowledge and awareness of rational antimicrobial prescriptions of Lao prescribers. AMS training sessions will be given to all prescribers in participating hospitals when they firstly enter to the intervention phase. This session will be given alongside an introduction of mobile phone app prescribing guideline. The training details will be based on the existing information in the paper-based therapeutic and prophylactic antimicrobial prescribing guidelines. The main part of the training will focus on good practices of antimicrobial prescription. Prescribers can also access information on AMS in both paper-based and mobile phone app prescribing guidelines by themselves. Providing training after introducing treatment guidelines is not routine practice in many hospitals in Laos. The combination of the AMS training and easy access to prescribing guidelines could be a new strategy to improve rational use of antimicrobial of Lao prescribers. For more details on how the training will be conducted see Appendix A12. *Antimicrobial Stewardship Training.*

c.3 Intervention delivery

The intervention period will start at month 4 after introducing the paper-based prescribing guidelines. This intervention will be introduced in one pair of hospitals at a time. The process will be repeated every 4 months until all six hospitals have access to the intervention. The order of the activities during intervention phase of each step (paired hospitals) are listed below:

1. PPS on hospital antimicrobial use (Appendix A1, A2 and A3)
2. AMS knowledge survey (Appendix A5)
3. Prescriber feedback survey on paper-based prescribing guidelines (Appendix A10)
4. Access to MicroGuide mobile phone app prescribing guidelines
5. Introductory session on MicroGuide mobile phone app prescribing guidelines (Appendix A7)
6. AMS training session (Appendix A12)
7. PPS reports (Appendix A4)

Note:

a. A survey on the mobile phone app prescribing guidelines (Appendix A11) will be performed at the end of the project only when all participating hospitals have access to the mobile phone app prescribing guidelines (month 16).

b. During the intervention period, prescribers in participating hospitals still have access to paper-based prescribing guidelines distributed at the start of the study. Mobile phone prescribing guidelines surveys (Appendix A11) will be used as a tool to access the use of the mobile phone app prescribing guidelines and the paper prescribing guidelines at the end of the study

Figure 2: Study procedures of SW-CRT on Evaluation of Lao-language antimicrobial use guidelines mobile app

	Pre-study phase	Study phase			
Hospital 6	PPS+Report	PPS+Report+Paper-based	PPS+Report+Paper-based	PPS+Report+Paper-based	PPS+Report+App+AMS
Hospital 5	PPS+Report	PPS+Report+Paper-based	PPS+Report+Paper-based	PPS+Report+Paper-based	PPS+Report+App+AMS
Hospital 4	PPS+Report	PPS+Report+Paper-based	PPS+Report+Paper-based	PPS+Report+App+AMS	PPS+Report+App+AMS
Hospital 3	PPS+Report	PPS+Report+Paper-based	PPS+Report+Paper-based	PPS+Report+App+AMS	PPS+Report+App+AMS
Hospital 2	PPS+Report	PPS+Report+Paper-based	PPS+Report+App+AMS	PPS+Report+App+AMS	PPS+Report+App+AMS
Hospital 1	PPS+Report	PPS+Report+Paper-based	PPS+Report+App+AMS	PPS+Report+App+AMS	PPS+Report+App+AMS
	Month 0	Month 4	Month 8	Month 12	Month 16

Activity at month 0	Activity at month 4	Activity at month 8	Activity at month 12	Activity at month 16
AMS survey for all sites	AMS survey*	AMS survey*	AMS survey*	AMS survey for all sites
Pre-prescribing survey	AMS training*	AMS training*	AMS training*	Prescriber feedback survey on the App guideline**
Introduce paper-based guideline	Introduce App guideline*	Introduce App guideline*	Introduce App guideline*	
	Prescriber feedback survey on paper-based guideline*	Prescriber feedback survey on paper-based guideline*	Prescriber feedback survey on paper-based guideline*	
		Prescriber feedback survey on the App guideline**	Prescriber feedback survey on the App guideline**	

* This activity will only be performed in a new selected paired hospital

** This activity will only be performed 4 months after introducing App guideline in each paired hospital

c.4 Contamination prevention

Although the clusters are geographically quite remote from each other, travel and communications between doctors in Laos is quite frequent. There is a possibility of contamination of the app to the reference group (however this is judged to be unlikely). The research team will record this if it occurs. Examples of how this could happen are:

- Doctors from the intervention group move to the reference group for any possible reason (training or changing workplace)
- Doctors from the intervention group give their smart phones to other doctors in the reference group (unlikely)
- Doctors from the intervention group lose their smart phones that are subsequently found and used by doctors in a reference group (unlikely)

To avoid or reduce the problem of contamination, at the start of the intervention phase, all participating hospitals will be requested to avoid sharing the mobile app prescribing guidelines to anybody outside their hospitals while they are still under evaluation. The app guidelines will be installed by the PI. This prescribing guidelines App will not be publicly available on the internet to download by users without the approval from the PI. Transferring the App from one phone to other device will be prevented by informatic code provided by the MicroGuide team. The functional system, security and app maintenance will be controlled by the MicroGuide team and PI of the project.

3.6 Endpoints

Primary endpoint

The proportion of antimicrobial prescriptions adherent with prescribing guidelines delivered by mobile phone app versus paper-based prescribing guidelines at month 16 (12 months' exposure in each group)

Secondary endpoints

1. Total antimicrobial defined daily doses (DDDs) prescribed in mobile phone app versus paper-based groups of 12 months' exposure in each group

2. Proportion of patients prescribed antimicrobials by the intravenous route in mobile phone app versus paper-based groups of 12 months' exposure in each group
3. AMS knowledge level of prescribers prescribed in mobile phone app versus paper-based groups of 12 months' exposure in each group
4. Prescriber satisfaction/acceptability of the prescribing guidelines in mobile phone app versus paper-based groups of 4 months' exposure in each group
5. Total costs of antimicrobial prescribed in mobile phone app versus paper-based groups of 12 months' exposure in each group
6. Proportion of patients admitted to hospital receiving an antimicrobial prescription before and after introduction of the paper-based prescribing guidelines; and the mobile phone app prescribing guidelines + AMS training of 12 months' exposure in each group

Definitions of adherence to the guidelines

1. Full adherence with the guidelines = using correct antimicrobial agent (s) and correct dose based on the provided antimicrobial prescribing guidelines

2. Partial adherence with the guidelines = using correct antimicrobial agent (s) with incorrect dose based on provided antimicrobial prescribing guidelines

3. Non-adherence with the guidelines = using a different antimicrobial agent (s) compared to that suggested in provided antimicrobial prescribing guidelines

3.7 Data collection method

Open Data Kit (ODK) system will be used as the data collection tool for PPS. Outcomes will be measured by regular PPS of hospital antimicrobial prescribing in both in- and outpatients (details as below) at all six hospitals during 4 monthly visits (five visits including one baseline data collection). Data will be collected using separate electronic questionnaires for in- and outpatients.

Paper case report forms (CRF) will be used for the prescriber surveys (Appendix A9. *Pre-prescribing guideline survey*; A10. *Prescriber feedback survey on paper-based prescribing guidelines*, A11. *Prescriber feedback survey on mobile phone app prescribing guidelines* and A5*. Antimicrobial stewardship knowledge survey*) will be distributed to all doctors in each study hospital in different time periods (Figure 2).

Each data collector will be trained on the study purpose/processes and the use of the data collection instruments before starting the project. IPD and OPD PPS data will be collected in ODK on patients' characteristics and treatment status, including age, sex, body weight, wards, antimicrobial agent (s), administration, dosage, unit, diagnoses, prescribing indication and antimicrobial start date. IPD PPS data will be collected directly from hospital charts and OPD PPS data will be obtained from the scanned prescription sheets. Data on the frequency of use of the mobile app can also be downloaded from the MicroGuide App.

Basic information (age, sex, specialty, departments, graduated year) of prescribers and their prescribing behaviors will be collected via survey forms (either prescriber feedback survey or AMS survey). All complete survey forms by prescribers will be returned to study team on the same day as the survey day. All data will be entered to MACRO system.

We will only consider antibiotic costs. Name of antimicrobials, dose, dosage form, manufacturing country of antibiotics will also be collected from each pharmacy hospital.

a. Point Prevalence Survey procedures

a.1 In-patient point prevalence survey

Design – Point prevalence survey

Survey site - All in-patient wards from all 6 participating hospitals:

1. Mahosot Hospital - 16 wards, including infectious disease paediatrics, general paediatrics, neonatal-paediatric intensive care unit (ICU), ICU adult, general medicine, gastro-intestinal, cardiovascular, infectious disease adult, lung, psychology, ear-nose-throat (ENT), abdominal surgery, uro-surgery, plastic surgery, general surgery, post operation room, obstetrics and gynaecology (OBGY)
2. LNT provincial hospital - 7 wards, including medicine, surgery, paediatrics, OBGY, intensive care unit, ENT
3. XK provincial hospital - 11 wards, including cardiology, infectious disease, OBGY, ICU adult, ICU paediatrics, surgery, post operation, medicine, eyes, and specialty ward (mixture of medicine, surgery, OBGY or paediatric patients – this ward is for patients who need private and more comfortable room)
4. VTP provincial hospital - 5 wards, including paediatrics, OBGY, medicine, surgery and ICU
5. SVK provincial hospital - 9 wards, including medicine 1, medicine 2, lung, infectious diseases (HIV/AIDs ward), surgery, OBGY, ICU and specialty ward
6. SV provincial hospital - 7 wards, including medicine 1 (general medicine), medicine 2 (mostly infectious disease patients), lung, paediatrics, surgery, OBGY and ICU

Survey dates and times – PPS will be conducted every four months throughout the year. There will be 5 PPS in total from the start of the project (month 0) until the finish date of the project (month 16) (Figure 2).

PPS must be completed within a maximum of 5 consecutive working days from the start date of collecting data in hospital of that PPS round. All hospital charts of all admitted patients on a ward must be screened at 8 o'clock in the morning on the day of the survey. Survey days will be from Monday to Friday, excluding public holidays.

Survey staff

- Research staff, *OR*

- Local doctor who is familiar with their own local hospital charts and has been trained on data collection for this project

Inclusion and exclusion criteria

Inclusion criteria

1. All patient hospital charts from all in-patient wards at 8:00 o'clock in the morning of a survey day will be counted as the denominator
2. All patient hospital charts from all in-patient wards with any kind of antimicrobial agent (s) prescribed at 8 o'clock in the morning of a survey day will be counted as numerator.

Definition for IPD surveys

- **Denominator**: all in-patients admitted on a ward on the survey day at 8:00 o'clock in the morning. Patients who are admitted on a ward after 8:00 am (8:01 am onward) of the survey day will be excluded from the survey
- **Numerator**: all in-patients admitted on a ward on the survey day at 8:00 o'clock in the morning who are prescribed an antimicrobial agent (s). Patients who are admitted on a ward after 8:00 am (8:01 am onward) of the survey day will be excluded from the survey, even if s/he is on antimicrobial agent (s)
- **On antimicrobial agent (s)**:
    - Documented antimicrobial agent (s) on a hospital chart on the survey day. This antimicrobial agent must be prescribed by 8:00am of the survey day
    - No antimicrobial agent is documented on the hospital chart on the survey day but the patient received an antimicrobial agent (s) within the previous 24 hours, or every 48 hours or once a week (any ongoing antimicrobial therapy or prophylaxis)

- **Surgical prophylaxis**: check 24 hours before surgical date to define surgical prophylaxis status as below:
    - Single dose prophylaxis (SP1) – patient received only single dose of antimicrobial agent before surgical procedure
    - One-day prophylaxis (SP2) – patient receives multiple doses of an antimicrobial agent within 24 hours. There are no further doses of prophylaxis after 24 hours
    - > One day prophylaxis (SP3) - patient received an antimicrobial agent as surgical prophylaxis for more than 24 hours (one day), regardless of how many doses a day

    - If a patient receives antimicrobial prophylaxis 48 hours before (or earlier) the survey day but no antimicrobial agent in the 24 hours before survey date or on survey day, this patient will be defined as "no antimicrobial agent"

- **Number of beds** = total number of beds in the ward (occupied + empty). Note: number of beds should usually be more than the number of inpatients at 8 o'clock. In case, there is an epidemic of some disease and the number of patients is more than the usual number of beds, count those extra beds (occupied beds + extra beds)

- **Antimicrobial start date:** this should be stated in the patient hospital chart before the survey start. Patients should be prescribed before 8:00am of the survey day. It is required the surveyor to check the previous record in the patient hospital chart

Exclusion criteria

- Patients who are admitted to a ward after 8:00 o'clock (8:01am) in the morning of the survey day
- Patients who are on topical antimicrobial agent(s) alone will be excluded from numerator but they will still be in the denominator

Survey setting

Research team will inform the hospital directors three days before starting each PPS (both IPD and OPD). Research staff will start collecting data on the ward at 8:00 o'clock in the morning of the survey day with permission of the hospital director few days before starting survey (both at IPD and OPD). The ward doctors and nurses will be informed about the survey on the same day as the survey day. Research staff will work on hospital charts only without interviewing any patients or ward staff, unless the number of charts and number of patients are not consistent or there is unclear handwriting of doctors or nurses. Research staff must complete the survey of a ward within a day. Research staff must finish a survey of one ward before moving to another ward.

If research staff cannot start the survey at 8:00 o'clock in the morning of the survey day for all wards, starting a new ward after 8:00am is acceptable with the condition of excluding all patients who are admitted to hospital after 8:00am (8:01am) of the survey day.

If some hospital charts are not available at the wards during the survey time (i.e. consulting with other medical specialties at other wards), the survey team can come back to that ward in the same day for that hospital chart but excluding all information after 8:00am of that survey day.

For more details of in-patient PPS data collection form, see **Appendix A1.** *In-patient PPS Ward Form* and **Appendix A2***. In-patient PPS Patient Form*.

a.2 Out-patient point prevalence survey

Design – Point prevalence survey

Survey site – In most hospitals, many wards have their own OPD within their wards. In this study, we will focus on three main wards (Paediatrics, Medicine and Surgery) where we would expect that the most antimicrobial prescribing would occur.

Survey dates and times – PPS will be conducted every four months throughout the year or three times a year with 4-month intervals between each survey. There will be 5 surveys in total. PPS in OPD will be performed for 5 consecutive working days in total from the start date of collecting data in the hospital in that PPS round.

All outpatient prescriptions will be screened between 8:00 o'clock in the morning and 4:00 o'clock in the afternoon on the day of survey. Survey days should be from Monday to Friday, excluding public holiday and weekends.

Surveyor qualification

- Research staff, *OR*
- Local doctor who received training for data collection for this project

Inclusion and exclusion criteria

Inclusion criteria

1. All patients consulting at OPD of paediatrics, medicine and surgical wards of all participating hospitals during 8:00am and 4:00pm of a survey day will be counted as the denominator

2. All patients consult at OPD of these three wards of all participating hospitals who are prescribed antimicrobial (s) between 8:00am and 4:00pm on a survey day will be counted as the numerator
3. If the same outpatient comes back for consultation during the same survey period (same round, any day during these five days) this patient will also be included in the survey and be defined as a new consultation.

Definitions for OPD surveys

- **Denominator**: all outpatients who consult at paediatrics, medicine and surgical OPDs on the survey day between 8:00am and 4:00pm for 5 consecutive working days. Patients who come outside this period of time will be excluded from the survey
- **Numerator**: all outpatients who consult at paediatrics, medicine and surgical OPDs and are prescribed antimicrobial (s) on the survey day between 8:00am and 4:00pm for 5 consecutive working days. Patients who come outside this period of time will be excluded from the survey, even if they are prescribed any antimicrobial (s).

Exclusion criteria

- Patients who come for consultation outside the survey time (before 8:00am or after 4:00pm) of the survey day
- Patient who are prescribed topical antimicrobial (s) only will be excluded from numerator but they will still be in the denominator

Study setting

OPD PPS will be conducted for 5 consecutive working days. Hospital directors of each hospital will be informed three days before the survey. Head of OPD will be informed about the survey on the same day as they survey day. Data will be collected from an OPD prescription sheet at the OPD pharmacy. There will not be any private discussion or interview of patients or doctors.

The OPD prescription sheet from patients will be scanned and saved as an electronic file for data entry by research staff on the same day. The reason for scanning OPD prescription sheets is to avoid disrupting the OPD workflow of the rush hours of each participating hospital. The total number of OPD patients (denominator) will be collected from the OPD logbook at the end of each survey day.

There are different systems of OPD data recording in each hospital. This project will not create a new system for the hospital but follow the existing routine systems of each hospital, ensuring that we can capture all antimicrobial prescription data and the denominators. Scanned prescription files will be securely kept and discarded one month after the scanned date.

For more details of outpatient PPS data collection form, see **Appendix A3.** Outpatient PPS Form.

Point prevalence survey report

PPS reports of IPD and OPD survey will be available within one month after each survey. With the agreement of MoH, these PPS will support antimicrobial use surveillance in the country. The reports will be in the format of computer dashboard (AMU-Laos App) which will be published on the MoH website. Access to AMU-Laos App will be publicly available. Everybody can view the results of the survey but not access the raw data. AMU-Lao App will be managed and operated by the PI and a dashboard programmer only. AMU-Laos App will display prevalence of antimicrobial prescriptions of each time points in different participating hospitals, as well as the overview of antimicrobial prescriptions (e.g. classes, agents, route and indications) and pattern of the prescription by age, by specialty in both IPD and OPD. Paper reports will be also available via this AMU-Laos App (Appendix A4. *Point Prevalence Survey (PPS) of hospital antimicrobial use report format*). However, paper reports will be also provided by the research team to the participating wards/doctors as there is limited access to computers and internet in some areas of Laos.

a.3 Antimicrobial stewardship (AMS) knowledge survey

This survey will be performed before introduction of the paper version of the therapeutic and prophylactic antimicrobial prescribing guidelines (at month 0) and every 4 months before the implementation of the mobile phone app guidelines and AMS training in the paired hospitals starting the intervention. The survey will be performed again in month 16 in all study hospitals before the study ends. Therefore, each hospital will participate this AMS knowledge survey 3 times during the whole study period (1st time= at the start of the project, 2nd time= before entering to the intervention period and 3rd time= at the end of the project)

This survey questionnaires will be in Lao language. The survey will provide information on the level of knowledge of rational antibiotic prescribing of prescribers before and after having access to paper prescribing guidelines and the app prescribing guidelines at different time points. For more details on what questions will be asked, see Appendix A5. *Antibiotic stewardship knowledge survey.*

Each survey will include 8 questions in total. The 8 questions will be randomly selected (simple random sampling – using a computer-generated random allocation sequence) from a set of questions in Appendix A5. The answers to all questions are in the guidelines.

Maximum 10 minutes will be requested to complete 8 questionnaires. The questionnaires will be multiple answers. There is no need to put the name of responders to the survey form but age, graduated year, department and speciality are needed. The survey will be conducted in the same day as introduction to the guidelines (paper (month 0)/app), introductory session of the guidelines (paper (month 0)/app), pre-prescribing guidelines survey (month 0), AMS training (only for those firstly enter to intervention period). Complete survey forms will be collected by the research team before starting the introductory session of the guidelines (paper (month 0)/app).

Pre-prescribing guidelines survey

The aim of conducting a pre-prescribing guidelines survey is to understand how prescribers prescribe antimicrobials and how much they use any prescribing guidelines in their routine work. This will provide some explanation as to what prescribers think about the provided prescribing guidelines. The survey will be performed once at the beginning of the study (month 0) after the first round of PPS and before introduction of the paper prescribing guidelines. Survey form (Appendix A9. *Pre-prescribing guidelines survey*) will be distributed to all prescribers at each study hospital and collecting back by the research team in the same day. The survey form contains 6 questions and it will take 5 minutes maximum to complete the survey.

Prescriber feedback survey on paper-based prescribing guidelines

Each participating hospital will participate this survey only once before they enter to the intervention period. The aim of the survey is to get feedback on the provided paper-based prescribing guidelines from the prescribers as well as self-reported use of the guidelines. Survey form (Appendix A10. *Prescriber feedback survey on paper-based prescribing guidelines*) will include questionnaires on the frequency of the use of paper-based guidelines and Likert scale questionnaires on prescribers' opinions on the paper-based prescribing guidelines. The survey forms will be distributed to all prescribers after PPS and before introduction of the mobile phone app prescribing guidelines. By doing this, we will obtain different opinions about the paper prescribing guidelines at different time points. This will also provide feedback on the guidelines and the change in prescribing behaviours in in participating hospitals exposed to paper and app

prescribing guidelines for the same duration. Complete survey forms will be collected from all prescribers at each study hospital in the same days as the survey day.

Prescriber feedback survey on mobile phone App prescribing guidelines

This survey will be conducted at the end of the study (month 16) when all participating hospitals having access to the mobile phone app at least for 4 months. The aim of the survey is to get feedback on the mobile phone app prescribing guidelines from prescribers, as well as how prescribers find this app compared to the paper version of the prescribing guidelines. The survey forms include questionnaires on the frequency of the use of paper-based guidelines after introducing mobile phone app guidelines, as well as the frequency of the use of the mobile phone app guidelines use and Likert scale questionnaires on prescribers' opinions of the mobile phone app prescribing guidelines. Complete survey forms will be collected from all prescribers at each study hospital on the same days as the survey day. The same survey form will be used for all participating hospitals (Appendix A11. *Prescriber feedback survey on mobile phone app prescribing guidelines*).

Data management and monitoring

All data will be entered by PI and trained research staff. All complete survey forms will be kept securely in a locked filling cabinet. Only the PI of the project and project data management team will have access to the raw data. All electronic data will be kept securely, protected by password access with automatic daily backup on the server at Lao-Oxford-Mahosot Hospital-Wellcome Trust Research Unit (LOMWRU) office and regular offsite tape backups. All scanned OPD prescription sheets will be appropriately destroyed within one month after the scanned date. Anonymized data from PPS will be fed back to the participating hospitals and Ministry of Health through a project-generated computer dashboard and reports one month after each PPS in each hospital. Data checking and cleaning will be done by the PI after each survey. Data inconsistency will be discussed with the research team immediately.

Statistical analysis

Normally distributed continuous baseline characteristics will be summarized with means and standard deviations. Skewed continuous data will be summarized using median and inter-quartile range. Categorical data will be presented as frequency and percentages. A logistic mixed effects regression model will be used to compare the proportion of antimicrobial prescriptions adherence

measured cross-sectionally at month 0, 4, 8, 12 until month 16 after introduction of prescribing guidelines by mobile phone application versus paper-based (30). The model will compare all wedges in the intervention from month 4 to month 16 with all wedges in the reference arm from month 0 to month 12. The duration of exposure of each hospital to the reference or intervention arm will be included in the model as interaction term with the study arm in order to assess whether the duration of exposure is the effect modifier. The model will consider the correlation among the outcome's measurements from the same hospital (cluster) by adjusting for clustering in the model. Odds ratios and the corresponding confidence intervals will be obtained and reported. Tests of significance will be performed at 5% level. Analyses will be done using Stata 15.0 or higher. Likert scale analysis will be used to evaluate prescriber satisfaction with/ acceptability of paper-based compared to mobile phone app delivered antimicrobial prescribing guidelines. The satisfaction/acceptability scale will be assigned in six categories, including strongly agree=5, agree=4, neutral=3, disagree=2 and strongly disagree=1, no opinion/not use=0. Mann-Whitney test will be used to assess the differences between paper-based population and mobile phone app population. AMS knowledge level of prescribers will be assessed using questionnaires based on information in the prescribing guidelines. The overall proportion of correct answers in each time point will be compared (baseline, after paper-based guidelines (before AMS training and introduction of mobile phone app guidelines) and after AMS training and introduction of mobile phone app guidelines). Other factors such as extra AMR training program from hospital/MoH/other organizations, level of education and specialties of prescribers will also be taken into account.

Cost of antibiotic therapy will be calculated from the start date of treatment to the survey day. The unit costs for the antibiotics will be obtained from the International Medical Product Price Guide.

Antibiotic defined daily dose (DDD) will be calculated using data (antibiotic agent, start date, dose and DDD of each antibiotic from www.whocc.no) from PPS in both IPD and OPD. Antimicrobial cost data from participating hospitals will also be collected each hospital pharmacy for comparison with international cost.

The results will be reported in accordance with the Consolidated Standards of Reporting Trials (CONSORT) extension for the stepped wedge cluster randomised trial (SWCRT) (30).

Safety considerations/Adverse Events

The intervention is the introduction of antimicrobial prescribing guidelines delivered by a mobile app in line with national and international recommendations. Therefore, no adverse event monitoring is planned.

Ethics

Ethical approval will be requested from both the University of Health Sciences Ethics Committee, Ministry of Health, Lao PDR and the Oxford Tropical Research Ethics Committee (OxTREC), University of Oxford, United Kingdom to conduct this project. Verbal consent to participate to the study will be asked from the director of each participating hospitals.

Benefits

This study describes the introduction of the first therapeutic and prophylactic antimicrobial prescribing guidelines in Laos. The guidelines will encourage the rational use of antimicrobials. Prescribing guidelines in the format of mobile phone application will allow clinicians to access guidelines easier. This will help to improve clinical decision of doctors and consistency of care. This will give the potential to improve health outcomes of patients, reduce morbidity and mortality and give value for money.

Risks

Guidelines have been developed based on existing national treatment guidelines, current data on infectious diseases, antimicrobial susceptibility patterns in Laos, as well as international recommendations. Availability of antimicrobials in Laos is also considered. The guidelines will be transformed to a mobile phone App which will make it easier for the clinicians to access. This would be more likely to benefit than harm patients or clinicians. However, this App and paper prescribing guidelines will be introduced to 6 general hospitals across Laos for 16 months before the prescribing guidelines mobile phone App will be adapted and distributed nationwide. There might be some risk that MoH would like to introduce the guidelines and the App before the study finishes if the results are very positive. In addition, it could be challenging to manage and control the travel of Lao doctors from one hospital to another as many trainings, workshops or conferences (not related to this project) are conducted across the country throughout the whole year. In addition, some doctors may have to leave their local hospital for further education. Apart from this project, another 18-month AMR program (supported by the Fleming fund) is also conducted in three participating hospitals (SV, XK and LNT) to improve the quality of infectious diagnosis in these three hospitals. This program plans to start at the same time as this project. This AMR program could have

some positive effect on antimicrobial stewardship knowledge of Lao doctors. In this case, the number of trainings and training materials (if applicable) provided by this AMR program will be recorded by our study team. There are also another three participating hospitals (MS, SVK and VTP) not included in the Fleming Fund project, this could allow us to compare MS knowledge level between hospitals with and without another AMR program in the analyzing stage.

Patient and doctor confidentiality

There will not be any record of patients' personal details to be able to identify any individual. Only patients' age, gender, weight and some information on antimicrobial prescription will be collected from hospital charts. There will not be any direct interaction between the research team and patients.

This study targets the behaviour of all prescribers rather than individuals. We will therefore focus on cluster information rather than individual data. Only prescribers' age, speciality, and the year of graduation will be collected as their personal details. Nobody (including research team and the PI of the study) will be able to identify the person who fills the survey form from a completed survey CRF or who prescribe antimicrobial (s) from ODK systems or database. All complete survey forms will be kept secured in the locked filing cabinet and all electronic data will be protected by password access.

Trial insurance

The University of Oxford has a specialist insurance policy in place: Newline Underwriting Management Ltd, at Lloyd's of London, which would operate in the event of any participant suffering harm as a result of their involvement in the research.

Data sharing and dissemination

This stepped wedge cluster randomized controlled trial will generate valuable data for healthcare sectors and for diverse further research projects in AMR field in Laos. This trial will provide data of hospital antimicrobial use across Laos, which have never been reported before. It will introduce a Lao language antimicrobial prescribing guidelines mobile app for the first time. This project will also provide data on cost of antibiotics use comparing between the paper-based and the mobile phone app prescribing guidelines. The study will also have global impact as an evaluation of an increasingly popular intervention that lacks a strong evidence base.

AMU-Laos App (computer dashboard) will be used to demonstrate real time results of the point

prevalence survey on hospital antimicrobial use at each stage to local authorities, the MoH and policy makers. AMU-Laos App can generate the report to the users with just one click.

Information on each PPS on hospital antimicrobial use will be shared with participating hospital staff and authorities and public health departments of each participating provinces, as well as MoH and funder one month after each survey.

The Lao-language antimicrobial use guidelines mobile application will be discussed with the MoH before starting the trial and only shared with doctors in participating hospitals during the trial period.

Preliminary results will be shared with the Lao Ministry of Health and the Wellcome Trust. Final results of the trial will be publicly available on the computer dashboard. Paper reports will be shared with participating hospitals, local public health offices, and the MoH. Oral presentation and posters will be used as tools to deliver progress updates and the results of the study in national, regional and international conferences. Results will also be published in open access peer reviewed medical journals.

Finance

This research is supported by the Wellcome Trust of Great Britain, UK. No funding sources have any role in study design, data collection, or preparation of the protocol.

References

1. WHO. Action Agenda for Antimicrobial Resistance in the Western Pacific Region: http://www.wpro.who.int/entity/drug_resistance/documents/action_agenda.pdf; 2015 [Available from: http://www.wpro.who.int/entity/drug_resistance/documents/action_agenda.pdf.

2. Munita JM AC. Mechanisms of Antibiotic Resistance. Microbiol Spectr. 2016;4(2).

3. Holmes A ML, Sundsfjord A, Steinbakk M, Regmi S, Karkey A, Guerin P, Piddock L. Understanding the mechanisms and drivers of antimicrobial resistance. The Lancet. 2015:387.

4. WHO. Global Action Plan on Antimicrobial Resistance: http://www.wpro.who.int/entity/drug_resistance/resources/global_action_plan_eng.pdf; 2015 [Available from: http://www.wpro.who.int/entity/drug_resistance/resources/global_action_plan_eng.pdf.

5. J ON. Tacking drug-resistant infections globally: Final report and recommendations. The review on antimicrobial resistance https://amr-review.org/sites/default/files/160518_Final%20paper_with%20cover.pdf2016 [Available from: https://amr-review.org/sites/default/files/160518_Final%20paper_with%20cover.pdf.

6. Limmathurotsakul D DS, Fukuda K, Feasey NA, Okeke IN, Holmes AH, Moore CE, Dolecek C, van Doorn HR, Shetty N, Lopez AD, Peacock SJ. Surveillance and Epidemiology of Drug Resistant Infections Consortium (SEDRIC). Improving the estimation of the global burden of antimicrobial resistant infections. Lancet Infect Dis. 2019;19(11):e392-e8.

7. M M. We Need Antibiotics. They're Not Profitable To Make. Who Pays? http://phenomena.nation-algeographic.com/2015/05/23/oneill-amr-3/: http://phenomena.nation-algeographic.com/2015/05/23/oneill-amr-3/; 2015 [Available from: http://phenomena.nation-algeographic.com/2015/05/23/oneill-amr-3/.

8. World-Bank-Group. Drug-Resistant Infections: A Threat to Our Economic Future [Discussion Draft]: http://pubdocs.worldbank.org/en/527731474225046104/AMR-Discussion-Draft-Sept18updated.pdf; 2016 [Available from: http://pubdocs.worldbank.org/en/527731474225046104/AMR-Discussion-Draft-Sept18updated.pdf.

9. WHO. Lao People's Democratic Republic: WHO statistical profile: https://www.who.int/countries/lao/en/; 2015 [Available from: https://www.who.int/countries/lao/en/.

10. WHO. Global Antimicrobial Surveillance System: Manual for early implementation: https://apps.who.int/iris/bitstream/handle/10665/188783/9789241549400_eng.pdf?sequence=1 ; 2015 [Available from: https://apps.who.int/iris/bitstream/handle/10665/188783/9789241549400_eng.pdf?sequence=1.

11. WHO. Global Priority of antibiotic-resistant bacteria to guide research, discovery, and development of new antibiotics: https://www.who.int/medicines/publications/WHO-PPL-Short_Summary_25Feb-ET_NM_WHO.pdf?ua=1; 2017 [Available from: https://www.who.int/medicines/publications/WHO-PPL-Short_Summary_25Feb-ET_NM_WHO.pdf?ua=1.

12. World-Bank-Group. Managing Transition, Reaching the vulnerable while pursuing universal health coverage: http://documents.worldbank.org/curated/en/160201512147494228/pdf/121809-REVISED-v1-Executive-Summary-07-12-17.pdf 2017 [Available from: http://documents.worldbank.org/curated/en/160201512147494228/pdf/121809-REVISED-v1-Executive-Summary-07-12-17.pdf

13. Lao-MoH. National Health Account Report, fiscal years 2012-2013, 2013-2014, 2014-2015 and 2015-2016 https://thinkwell.global/wp-content/uploads/2018/04/Lao-NHA-2013-2016-report-_Final-Eng.pdf: Ministry of Health (MoH); 2017 [1-33]. Available from: https://thinkwell.global/wp-content/uploads/2018/04/Lao-NHA-2013-2016-report-_Final-Eng.pdf.

14. WHO. The Lao People's Democratic Republic health system review https://iris.wpro.who.int/bitstream/handle/10665.1/10448/9789290616481_eng.pdf; 2014 [Available from: https://iris.wpro.who.int/bitstream/handle/10665.1/10448/9789290616481_eng.pdf.

15. Peacock SJ NP. Public health impact of establishing the cause of bacterial infections in rural Asia. Trans R Soc Trop Med Hyg. 2008;102(1):5-6.

16. Lao-MoH. Structure of th national laboratory network. Operational guidelines for health laboratories networking in Lao PDR (version 1). 1 ed: Lao-Ministry of Health; 2015. p. 4.

17. Cusack TP PV, Duangmala K, Phoumin P, Turton J, Hopkins KL, Woodford N, Shetty N, Stoesser N, Phan HTT, Dance DAB. Molecular characterizationof carbapenem-resistant Escherichia coli and Acinetobacter baumannii in the Lao People's Democratic Republic. 2019;74(9):2810-21.

18. Roberts TR, Sayaphet & Phommasone, Koukeo & Chansamouth, Vilada & Davong, Viengmon & Valy, Keoluangkhot & Hongsakhone, Sitthivone & Bounsavath, Naly & Mayxay, Mayfong &

Vongsouvath, Manivanh & Dance, David & Newton, Paul. Typhoid in Laos: An 18-Year Perspective. The American Journal of Tropical Medicine and Hygiene. 2020;102(4):749.
19. Keohavong B SL, Sengaloundeth S, Nishimura A, Ito K. Rational use of drugs: prescribing and dispensing practices at public health facilities in Lao PDR. Pharmacoepidemiol Drug Saf. 2006;15(5):344-7.
20. Mayxay M C-VJ, Chansamouth V, Dubot-Pérès A, Paris DH, Phetsouvanh R, Tangkhabuanbutra J, Douangdala P, Inthalath S, Souvannasing P, Slesak G, Tongyoo N, Chanthongthip A, Panyanouvong P, Sibounheuang B, Phommasone K, Dohnt M, Phonekeo D, Hongvanthong B, Xayadeth S, Ketmayoon P, Blacksell SD, Moore CE, Craig SB, Burns MA, von Sonnenburg F, Corwin A, de Lamballerie X, González IJ, Christophel EM, Cawthorne A, Bell D, Newton PN. Causes of non-malarial fever in Laos: a prospective study. Lancet Glob Health. 2013;1(1):e46-54.
21. Quet F VE, Leyer C, Buisson Y, Newton PN, Naphayvong P, Keoluangkhot V, Chomarat M, Longuet C, Steenkeste N, Jacobs J. Antibiotic prescription behaviours in Lao People's Democratic Republic: a knowledge, attitude and practice survey. Bull World Health Organ. 2015;93(4):219-27.
22. Keohavong B VM, Phoummalaysith B, Louangpradith V, Inthaphatha S, Kariya T, et al. Antibiotic prescription for under-fives with common cold or upper respiratory tract infection in Savannakhet Province, Lao PDR. Tropical medicine and health. 2019;47(1):16.
23. Wang H WH, Yu X, Zhou H, Li B, Chen G, Ye Z, Wang Y, Cui X, Zheng Y, Zhao, R YH, Wang Z, Wang P, Yang C, Liu L. Impact of antimicrobial stewardship managed by clinical pharmacists on antibiotic use and drug resistance in a Chinese hospital, 2010-2016: a retrospective observational study. BMJ Open. 2019;9(8):e026072.
24. Scheetz MH BM, Postelnick M, Noskin GA, Lee TA Cost-effectiveness analysis of an antimicrobial stewardship team on bloodstream infections: a probabilistic analysis. J Antimicrob Chemother. 2009;63(4):816-25.
25. Tuon FF GJ, Wollmann LC, Moraes TP. Mobile health application to assist doctors in antibiotic prescription - an approach for antibiotic stewardship. Braz J Infect Dis. 2017;21(6):660-4.
26. Yoon CH RS, Duffy EJ, Thomas MG, McBride S, Read K, Chen R, Humphrey G. Impact of a smartphone app on prescriber adherence to antibiotic guidelines in adult patients with community acquired pneumonia or urinary tract infections. PLoS One. 2019;14(1):e0211157.
27. Antonello VS CM, Rosin ET, Castilhos JX, Beduschi FG, Bumaguin DB, Antonello ICF, Jimenez MF. The Use of Mobile Educational Tools to Improve Antimicrobial Prescription for the Treatment of

Acute Pyelonephritis in Pregnancy: A Retrospective Cross-sectional Study. Rev Bras Ginecol Obstet. 2019;41(2):97-101.

28. Panesar P JA, Aldous A, Kranzer K, Halpin E, Fifer H, Macrae B, Curtis C, Pollara G. Attitudes and Behaviours to Antimicrobial Prescribing following Introduction of a Smartphone App. PLoS One. 2016;11(4):e0154202.

29. Charani E GM, Moore LSP, Castro-Sanchéz E, Lawson W, Gilchrist M, Holmes AH. Effect of adding a mobile health intervention to a multimodal antimicrobial stewardship programme across three teaching hospitals: an interrupted time series study. J Antimicrob Chemother. 2017;72(6):1825-31.

30. Hemming K, Taljaard, M., & Forbes, A. Analysis of cluster randomised stepped wedge trials with repeated cross-sectional samples. 2017;18(1):101.

Appendices

A1. In-patient PPS Ward form

Evaluating the impact of a Lao-language antimicrobial prescribing guidelines on antimicrobial prescribing in Laos

Hospital Antimicrobial Prescribing Survey Form

Ward Form

**Date of Survey** (dd-mmm-yyyy)		**Surveyor name**	☐ Vilada	☐ Danoy	☐ ________	
**Hospital name**	☐ Mahosot	☐ VTP	☐ SVK	☐ SV	☐ XK	☐ LNT
**OPD/IPD**	☐ OPD	☐ IPD		**Ward name**		
**Department type**						

☐	Medicine
☐	Surgery
☐	Paediatrics
☐	OBGY
☐	ICU

**Total number of admitted patients by 8:00am on the day of survey**	Numeric
**Total number of bed (including extra bed)**	Numeric

A2. In-patient PPS Patient Form

**Evaluating the impact of a Lao-language antimicrobial prescribing guidelines on antimicrobial prescribing in Laos**
**Hospital Antimicrobial Prescribing Survey Form**

In-Patient Form

Survey number	Activity	Age (≥2years)	Age (1-23m)	Age (<30days)	Weight (kg)	Gender (M/F)
	M S IC					

	Antimicrobial 1	Antimicrobial 2	Antimicrobial 3	Antimicrobial 4	Antimicrobial 5
Antimicrobial name					
Antimicrobial start date					
Single Unit Dose					
Unit (g, mg, IU)					
Doses/day					
Route (P, O, R)*					
Diagnosis					
Diagnosis site					
Type of Indication					
Reason in Note (yes/no)					
Is a stop/review date documented? (yes/no)					
Treatment (empiric/targeted)					
**The next section is to be filled in only if the treatment choice is based on microbiology data (Treatment=targeted) AND the organism is one of the following**					
MRSA (yes/no)					
VRE (yes/no)					
ESBL-producing Enterobacteriaceae (yes/no)					
3rd generation cephalosporin resistant Enterobacteriaceae non-ESBL producing or ESBL status unknown (yes/no)					
Carbapenem-resistant Enterobacteriaceae (yes/no)					
ESBL-producing non-fermenter Gram-negative bacilli (yes/no)					
Carbapenem-resistant non-fermenter Gram- negative bacilli (yes/no)					
Targeted treatment against other MDR organisms (yes/no)					
Targeted treatment against other confirmed laboratory results (yes/no)					
Treatment based on biomarker data (yes/no)					
If yes, which biomarker (CRP, PCT or other)		Value		Unit (in μg/L, mg/L)	

Remark: One In-patient PPS Ward Form is for one ward. One In-patient PPS Patient Form is for one patient who is on antimicrobial agent (s). No In-patient PPS Patient Form will be filled for patients with any antimicrobial agent (s) as defined in this study. * P=Parenteral, O=Oral, R=Rectal.

A3. Outpatient PPS Form

Evaluating the impact of a Lao-language antimicrobial prescribing guidelines on antimicrobial prescribing in Laos

Hospital Antimicrobial Prescribing Survey Form

Outpatient Form

**Date of Survey** (dd-mmm-yyyy)	Add calendar	**Surveyor name**	□ Vilada	□ Danoy	□ ________	
**Hospital name**	□ Mahosot	□ VTP	□ SVK	□ SV	□ XK	□ LNT
**OPD/IPD**	□ OPD	□ IPD		**OPD type**	Drop down list	

Total number of consulted patients between 8:00 am and 4:00pm on the day of survey Numeric

Survey number	Activity	Age (≥2years)	Age (1-23m)	Age (<30days)	Weight (kg)	Gender

	Antimicrobial 1	Antimicrobial 2	Antimicrobial 3	Antimicrobial 4	Antimicrobial 5
Antimicrobial name					
Single Unit Dose					
Unit (g, mg, IU)					
Doses/day					
Route (P, O, R)					
Diagnosis					
Diagnosis site					
Type of Indication					
Reason in Note (yes/no)					
Is a stop/review date documented? (yes/no)					
Treatment (empiric/targeted)					
If targeted, specify:					
Antimicrobial stop date					

A4. Point Prevalence Survey (PPS) of hospital antimicrobial use Report Format

All participant hospitals will receive PPS report in the same format with their own hospital antimicrobial use data. The report will be available in both Lao and English languages. Participating hospitals will have access to both current and previous reports via AMU-Laos App. We would like to keep this report in a friendly format for all doctors with and without statistical knowledge background.

Lao People's Democratic Republic

Peace, independence, democracy, unity and prosperity

****

Ministry of Health

Lao-Oxford Mahosot Hospital

Wellcome Trust Research Unit (LOMWRU)

Point Prevalence Survey (PPS) of hospital antimicrobial use, [name of hospital], [name of province], Lao PDR

Survey date:	[start date] to [stop date]	
Survey team:	[name of surveyor]	Editor: Vilada Chansamouth
	[name of surveyor]	
	[name of surveyor]	

I. Background

This Point-prevalence survey (PPS) on hospital antimicrobial use in Laos has been developed based on the Global-PPS (www.global-pps.com) which was developed in 2006. Laos participated in Global-PPS in 2017 to provide the first data of hospital antimicrobial prescribing of Lao doctors to the country as a part of a project on surveillance of antimicrobial use and resistance in Laos. In 2017, four hospital participated in this survey (Mahosot Hospital, Xiengkhuang, Luang Namtha and Salavan Provincial Hospitals). Vientiane provincial Hospital joined the survey in 2018 and in 2019 Savannakhet Provincial Hospital also participated. There are now six hospitals in across Laos who participate in this PPS on hospital antimicrobial use.

II. Survey objectives

To provide hospital antimicrobial use data to participating hospitals and the country

To promote the rational use of antimicrobials in Laos

To contribute to national antimicrobial use and resistance surveillance

III. Survey methods

1. **Surveyed hospitals**: this PPS on hospital antimicrobial use in Laos was conducted in six hospitals, including Mahosot Hospital, Vientiane, Xiengkhuang, Luang Namtha, Savannakhet and Salavan Provincial Hospitals.
2. **Survey wards**: all in- and outpatient departments of each participant hospitals participated in this survey
3. **Survey date and time**: PPS on hospital antimicrobial use was conducted every 4 months.
   - **3.1. In- patient department (IPD):** All patient hospital charts from each IPD were screened at 8:00 o'clock in the morning of the survey day. Survey days were from Monday to Friday, excluding weekends and public holidays
   - **3.2. Outpatient department (OPD):** PPS in OPD (medicine, paediatrics and surgery) was conducted for 5 consecutive working days from 8:00am to 4:00pm, excluding weekends and public holidays

4. **Inclusion criteria**: all IPD and OPD patients (medicine, paediatrics and surgery) who were prescribed antimicrobial (s) during the defined survey period
5. **Data collection**: basic data on patient characteristics (age, sex, weight), information on antimicrobial prescriptions (e.g. dose, route, duration and indication) were collected. Neither doctors/nurses nor patients were interviewed in this survey.

IV. Survey findings

This survey was conducted from [start date to stop date] in [hospital name]. The survey was performed by [number of surveyors] in [number of in-patient wards] IPD and one OPD. During [number of survey days] days of IPD survey, [number of screened hospital charts] hospital charts were screened, of these, [number of patients with prescribed antimicrobial (s)] ([%]) received some kinds of antimicrobial (s). During 5 consecutive working days of the OPD survey, [number of prescriptions] were screened, of these [number of patients with prescribed antimicrobial (s)] ([%]) received antimicrobial (s). More details of prescriptions can be found as below:

Overall antimicrobial prescription by quarter at [name of hospital]

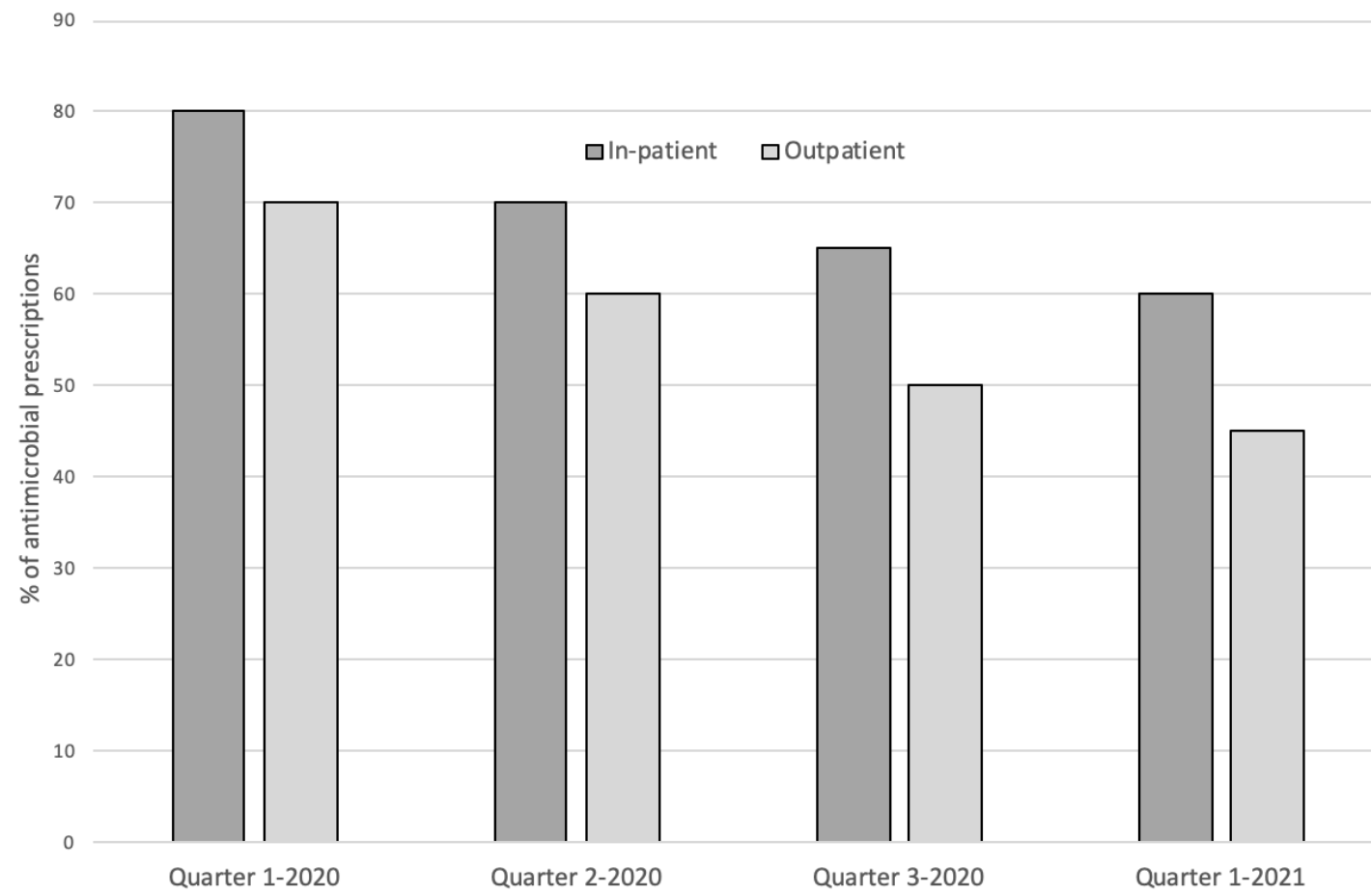

Overview of Point Prevalence Survey of hospital antimicrobial prescription at [name of hospital] from [survey start date] to [survey stop date]

Description	Number of in-patients (%)	Number of outpatients (%)
Number of screened hospital charts		
Number of patients with antimicrobial (s)		
Department:		
OPD	NA	
Medicine		NA
Surgery		NA
ICU		NA
Paediatrics		NA
OBGY		NA
Age:		
≤ 1 year		
>1- ≤ years		
>5 - ≤ 15 years		
>15 years		
Number of prescribed antimicrobials		
1 antimicrobial agent		
2 antimicrobial agents		
≥3 antimicrobial agents		
Total number of prescribed antimicrobial agents		
Administration route		
Parenteral		
Oral		
Rectal		
Prescribing indication		
Therapeutic		
Prophylactic		
Empirical treatment		
Documented reason for treatment (diagnosis)		
Documented stop/review date		

Overall proportion of patients receiving an antimicrobial prescription by antimicrobial class at [name of hospital] from [survey start date] to [survey stop date]

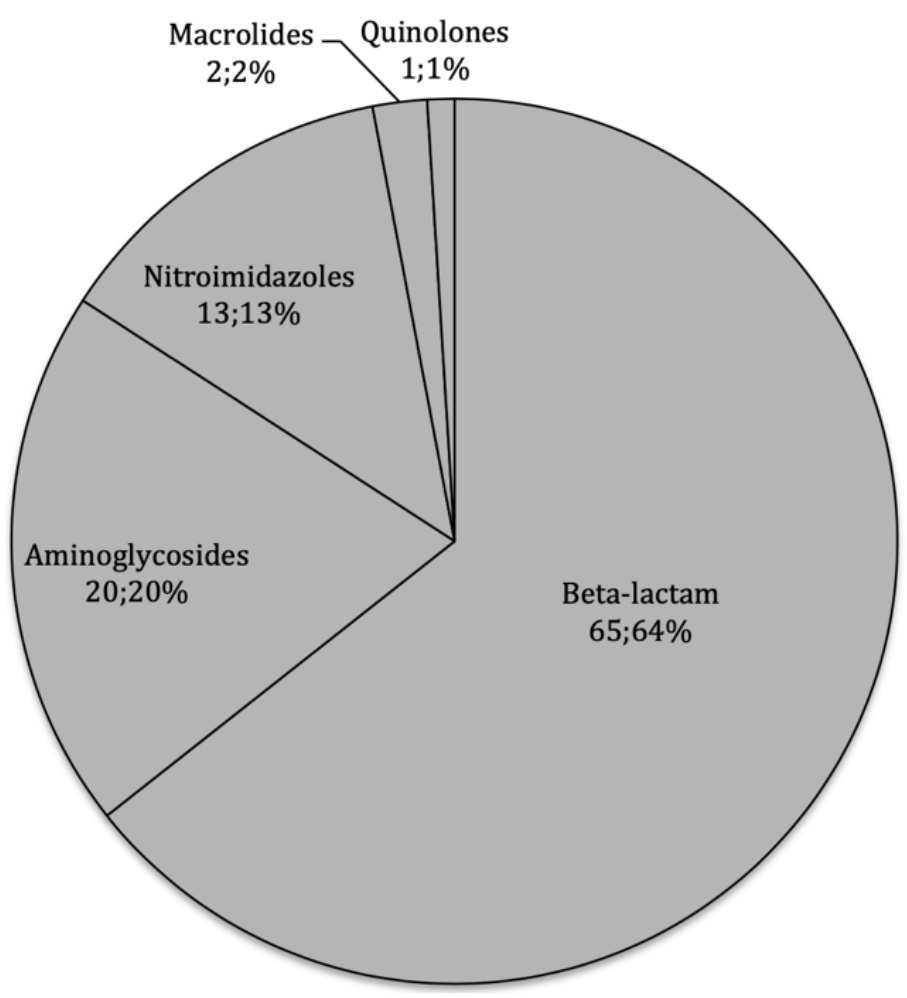

Overall proportion of antimicrobial prescriptions in outpatient ward

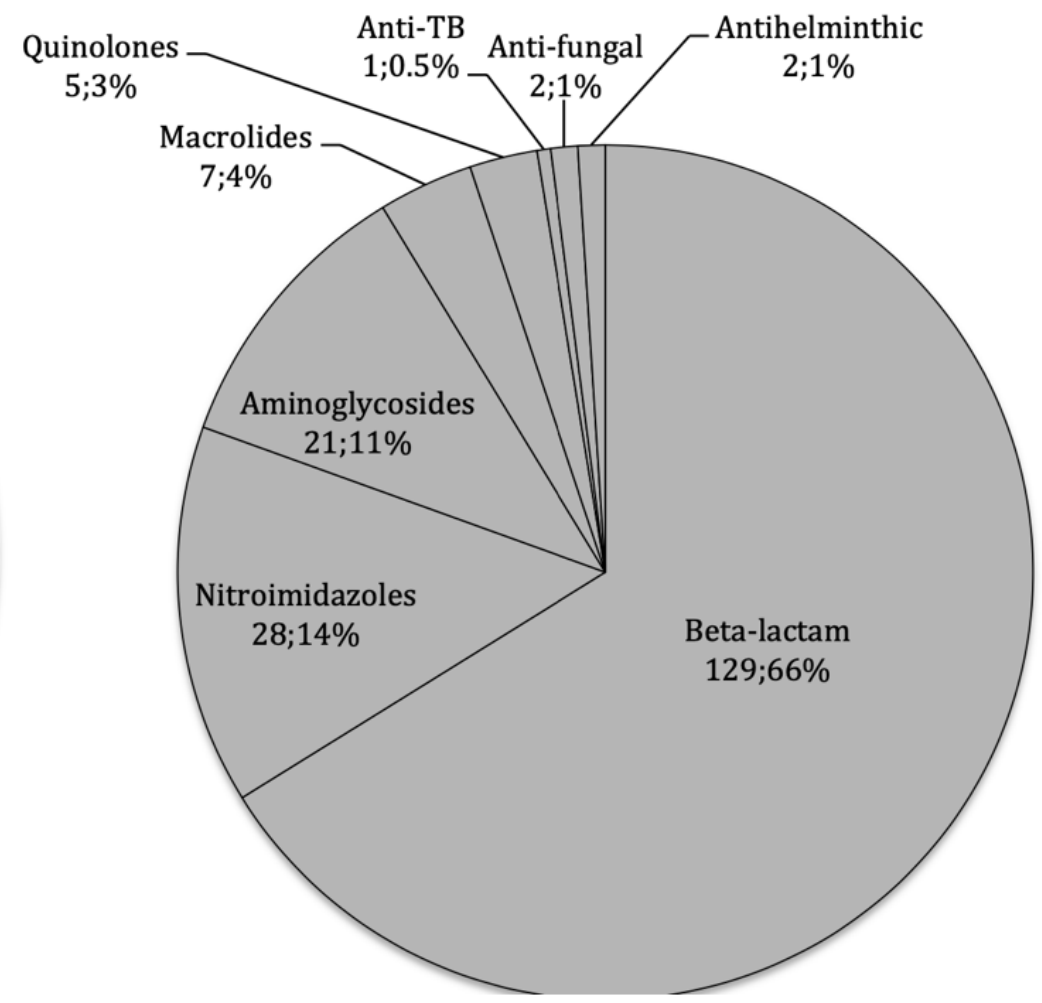

Overall proportion of antimicrobial prescriptions in in-patient ward

Overall antimicrobial prescription by antimicrobial class and age at [name of hospital] from [survey start date] to [survey stop date]

In- patients [not real data - example chart only]

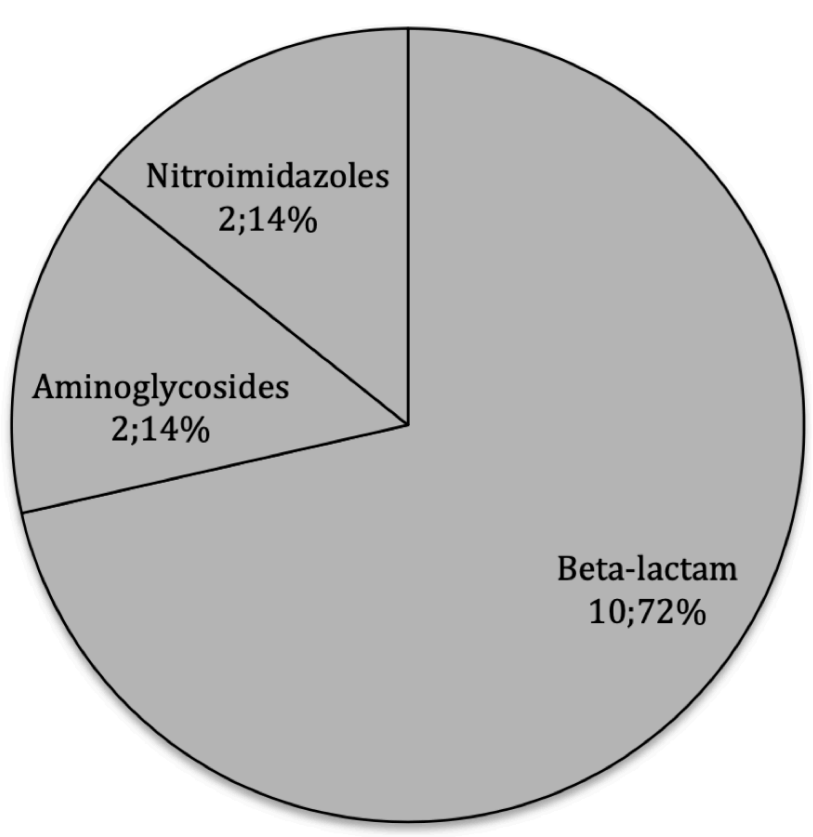

Proportion of antimicrobial prescriptions in children aged ≤ 15years

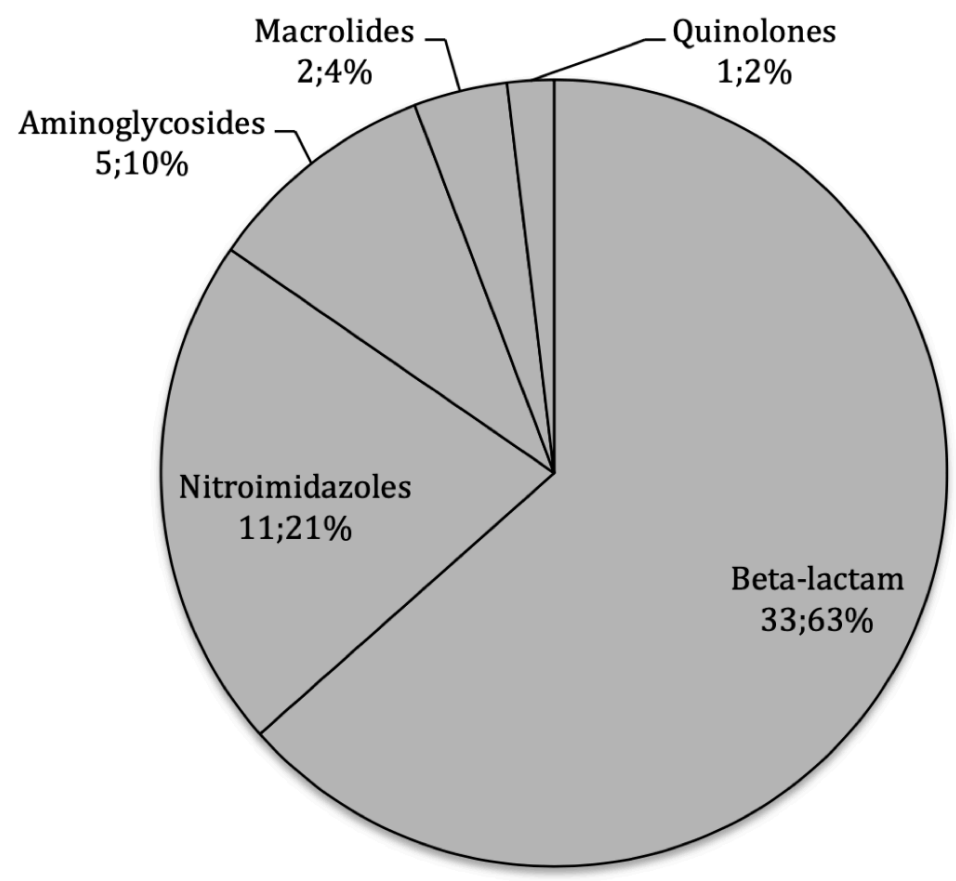

Proportion of antimicrobial prescriptions in adult aged > 15years

Outpatients [not real data - example chart only]

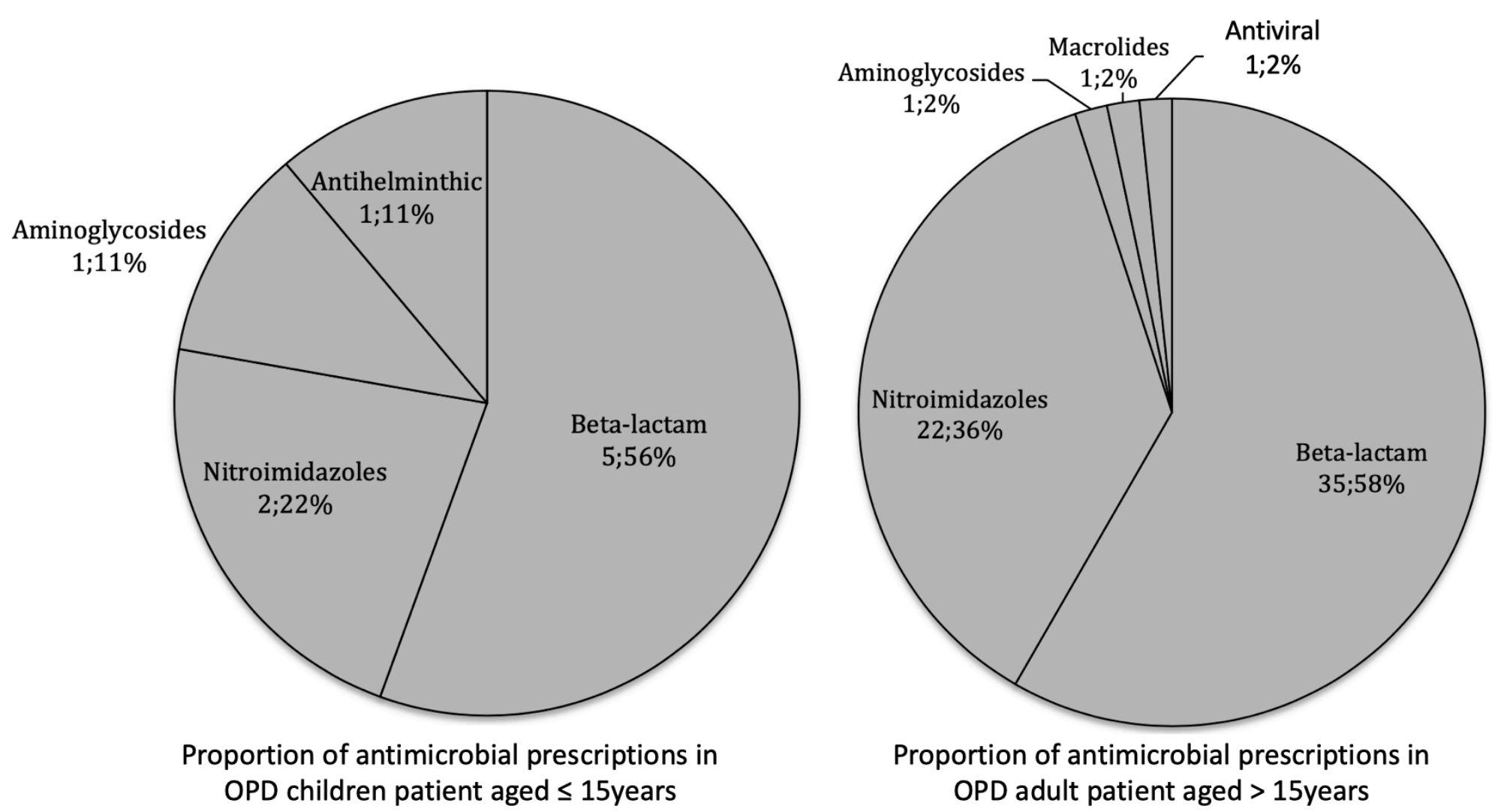

Overall antimicrobial prescription by 5 most common involved organs or systems

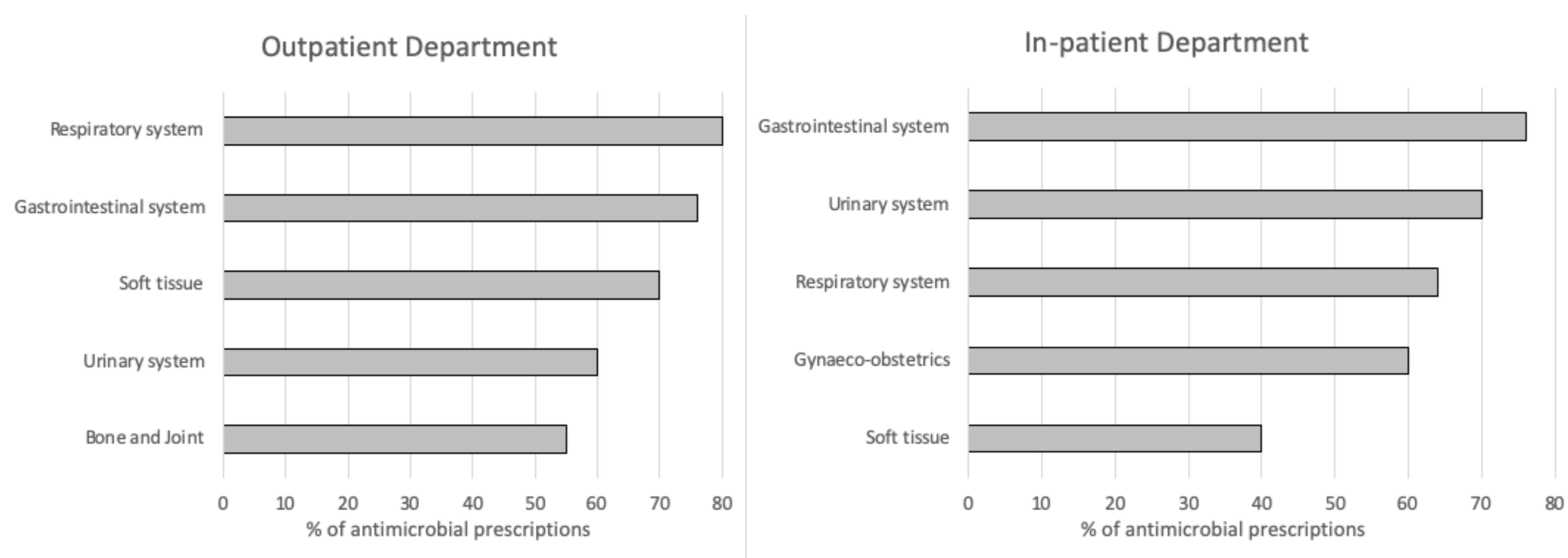

List of antimicrobial prescriptions at [name of hospital] from [survey start date] to [survey stop date]

Antimicrobial classes	Antimicrobial agent	Number of in-patient prescription (%); n= [total number of prescriptions]	Number of OPD prescription (%); n= [total number of prescriptions]
Beta lactams	Cephalosporins		
	[agent list]		
	Penicillin		
	[agent list]		
	Carbapenem		
	[agent list]		
	Beta-lactam inhibitors		
	[agent list]		
Aminoglycosides	[agent list]		
Quinolones	[agent list]		
Macrolides	[agent list]		
Sulfonamides	[agent list]		
Nitroimidazoles	[agent list]		
Tetracycline	[agent list]		
Antiviral	[agent list]		
Antifungals	[agent list]		
Anti-TB	[agent list]		
Antimalarial	[agent list]		
Anti-helminthic	[agent list]		

A5. Antimicrobial stewardship (AMS) Knowledge survey

Antibiotic stewardship (AMS) knowledge survey

(Multiple answers are possible. Please circle your answers)

Date: ____/____/______, Graduation year from medical school: ______________ Speciality: ______________

Department: ________________, Hospital: ______________

1. **Facts of antimicrobial resistance (AMR):**
   a. Overuse or misuse of antimicrobials is accelerating the process of AMR
   b. AMR can occur naturally
   c. AMR can spread between people, animals and environment
   d. Poor infection control encourages the spread of AMR

2. **Misuse or overuse of antibiotics include:**
   a. Prescribing an oral narrow spectrum antibiotic (e.g. amoxicillin) to treat the common cold
   b. Always using broad spectrum antibiotics as first choice
   c. Completing an antibiotic treatment course even though the patient is feeling better

3. **Which of the following is/are NOT correct?**

   a. Antibiotics kill both good and bad bacteria
   b. Antibiotics kill both viruses and bacteria
   c. Humans can become resistant to antibiotics
   d. Bacteria can become resistant to antibiotics

4. **What is considered as good practice in antibiotic prescribing in general practice?**
   a. Prescribe antibiotics with a clear clinical justification where a bacterial infection is suspected or proven
   b. Prescribe according to local antimicrobial susceptibility patterns
   c. Only prescribe when culture results are available
   d. Collect specimens for culture before starting antimicrobial therapy (but not delay therapy)

5. **What should consider before prescribing antibiotics?**
   a. Previous antibiotic history
   b. Previous infection with drug resistant organism
   c. Allergy to antibiotic
   d. Availability of antibiotic

6. **What should be consider when reviewing empiric antibiotic therapy?**
   a. Change antibiotics based on culture result
   b. Always keep IV therapy until patients discharge from hospital, then switch to oral
   c. Stop antibiotic if no evidence of infection
   d. Change to broader spectrum antibiotics if patients do not feel better

7. **Which of the following phrases is correct for choice of IV or oral antibiotic therapy?**
   a. Always start IV antibiotic first, then switch to oral antibiotic for all in-patients
   b. IV antibiotic needs to be kept for at least 3 days before switching to oral antibiotic
   c. Keep IV therapy for the whole course of bacterial meningitis treatment

8. **What should be considered before switching from IV to oral therapy?**
   a. Equivalent treatment efficacy
   b. No malabsorption problem
   c. availability of suitable oral antimicrobial drug
   d. Signs and symptoms of infection improving

9. **Which of the following antimicrobials are well absorbed from the gut and have excellent penetration into cells and tissues?**
   a. Co-amoxiclav (e.g augmentin)
   b. Metronidazole (e.g. flagyl)
   c. Ciprofloxacin
   d. Chloramphenicol

10. **What are clinical signs of severe penicillin allergy (type I)?**
   a. Anaphylaxis
   b. Maculopapular rash

c. Laryngeal oedema
d. Usually occurs with 1 to 4 hours after exposure

11. What medications are contradicted in patients with severe penicillin allergy?

a. Co-amoxiclav (e.g augmentin)
b. Metronidazole (e.g flagyl)
c. Ciprofloxacin
d. Cloxacillin

12. Antibiotic prophylaxis for elective surgery should usually be given for:

a. A maximum of 24 hours
b. A maximum of 3 days
c. A maximum of 7 days
d. Until discharge from hospital

13. Good practice for clean wound/surgery procedures regarding antibiotic prophylaxis is:

a. No antibiotic prophylaxis is needed
b. A single dose of IV combined antibiotic prophylaxis (e.g. ceftriaxone + metronidazole) is needed
c. IV mono-antibiotic prophylaxis for a maximum of 3 days is enough
d. A maximum of 5 days of oral antibiotic prophylaxis is needed

14. Which phrases are correct relating to viral respiratory infection:

a. Antibiotics should be given to prevent bacterial complications in seasonal flu patients
b. Antibiotics have no effect on colds
c. Empirical treatment of bacterial co-infection of seasonal flu, should be for highly suspected cases only

15. A 3-year-old girl comes to you complaining of 4 days of fever, running nose, sneezing, sore throat, cough with normal vital signs, which antibiotic you recommend?

a. Amoxicillin
b. Penicillin

c. Single dose of ceftriaxone
d. No antibiotic is needed

16. Which statements are correct relating to the use of gentamicin?

a. Gentamicin can cause serious nephrotoxicity and ototoxicity
b. Gentamicin is often used for serious Gram-negative bacterial infections
c. Gentamicin is cleared by the kidney, it is important to assess renal function before starting and during treatment courses
d. Creatinine clearance should be monitored if drug level monitoring is not available

17. Which of the following is NOT effective in preventing the emergence or spread of antimicrobial resistant organisms?

a. Good hand hygiene
b. Treating infections for a longer period to prevent resistance
c. Avoiding the use of antibiotics for treatment of colds and flu
d. Vaccination

18. A 35-year-old man comes to you complaining of 2 days watery stool (~4-5 times a day), no history of fever, which antibiotic will you recommend?

a. Ciprofloxacin
b. Co-trimoxazole
c. Azithromycin
d. No antibiotic is needed, oral rehydration only

A6. Introductory session of therapeutic and prophylactic antimicrobial prescribing guidelines

The propose of the session is to introduce paper versions of therapeutic and prophylactic antimicrobial prescribing guidelines in both adult and paediatric version to all doctors in study hospitals. This session will not cover how to diagnose infectious diseases or prescribe antimicrobials.

Trainees: this session will be given to all hospital doctors in all study participant hospitals at the start of the study (month 0).

Date, time and venue: the session will take a maximum of 30 minutes with 10 minutes of discussion/questions. Session date, time and venue will be informed to all doctors one week in advance and again 3 days before starting the session. The session will be run in each hospital separately after conducting point prevalence survey (PPS) of hospital antimicrobial use and after all doctors have completed survey form on AMS knowledge for baseline data. Verbal consent will be asked from participants to record participant's names, age, sex, working positions, departments, years of work and phone number onto the session registration sheet.

Instructor: The principal investigator of the study will be the session instructor. Training will be given in the format of a PowerPoint presentation, following by question and discussion.

Session material: paper versions of both adult and paediatric prescribing guidelines will be distributed to doctors who participate to this session. A handout of the introductory session (the same standard session for all hospitals) will also be provided.

Session details include introduction of the guidelines, objectives of guidelines, guidelines skeleton and how to use these guidelines, see details as below:

1. **Introduction to the guidelines**

This guideline is based on:

- existing Lao treatment guidelines
- current data on infectious disease pathogens and antimicrobial susceptibility patterns in Laos
- international antimicrobial prescribing/treatment guidelines (e.g. WHO, CDC, NICE, IDSA, Sanford)

- both Lao and international clinicians, international infectious diseases experts and microbiologists contributed to this guideline

2. **Giving the purpose of the having these guidelines:**
    - To support therapeutic clinical decision-making of Lao clinicians
    - To guide the reasonable use of antimicrobials to optimize cure and control antimicrobial resistance in Laos
    - To promote the use of essential medicines registered with the Lao Food and Drug Department
    - To diminish the cost of care for infectious diseases whilst ensuring optimal treatment
    - To promote patient safety by diminishing risks of allergy or toxicity
    - To promote the use of microbiology laboratory techniques for infectious disease diagnoses and for guiding treatment

3. **Explanation of overall guideline skeleton**
    - Therapeutic section
    - Prophylactic section
    - AMS section
    - References
    - Contact details
4. **How to use these guidelines**

- Guidelines provide the principal uses of antimicrobials. The antimicrobial prescribing guidelines are separated into paediatric and adult guidelines for ease of use.
- These guidelines include recommendations for therapy, prevention and AMS
- This guideline mostly suggests empiric treatment as facilities to microbiological diagnosis in Laos is limited

Structure:

- Common and important infectious diseases are arranged by involved organ systems

- Each infectious disease section includes causative pathogen(s) of the diseases, choices of antimicrobial regimen (primary and alternative), doses, duration and important notes on allergy or toxicity or to help guide treatment
- Sources of information and contact details of developers and coordinators are displayed at the end of the guidelines for users who need further information
- This guideline also refers to some specific Lao treatment guidelines (e.g. Dengue guideline for diagnosis and treatment, Lao PDR or National Guidelines for the Use of Antiretroviral Therapy in Adults and Children) to avoid duplication

A7. Introductory session of antimicrobial prescribing guidelines mobile phone application (MicroGuide)

Introductory session of the antimicrobial prescribing guideline mobile phone App will be given to all doctors at study hospitals in different time periods.

Training objective is to introduce MicroGuide mobile phone App of therapeutic and prophylactic antimicrobial prescribing guidelines in both adult and paediatric version to all doctors in study hospitals. This session will cover how to install, register, use and update the App. The session will not cover how to diagnose infectious diseases or prescribe antimicrobials.

Trainees: this session will be given to all hospital doctors in all study hospitals at the start of the intervention period of each pair of hospitals (month 4, month 8 and month 12).

Training date, time and venue: the session will take maximum 4±1 hours in total (including installation and registration step by step, also include discussion/questions). Training date, time and venue will be informed to all doctors one week in advance and again 3 days before starting the session. The session will be run in each hospital separately (2 hospitals on month 4, another 2 on month 8 and the last 2 hospitals on month 12) after conducting point prevalence survey (PPS) of hospital antimicrobial use. Verbal consent will be asked from participants to record each participant's name, age, sex, working position, department, years of work and phone number onto the session registration sheet.

Instructor: the principal investigator of the study will be the training instructor. Training will be given in the format of PowerPoint presentation and showing the real process of each step, followed by questions and discussion.

Training material: A handout of the training will be provided to all participants (the same standard session for all hospitals).

Training details: training session will include introduction to the MicroGuide, how to register, how to install, how to use step by step and how to update the App.

A8. Introductory (paper/App guidelines) session registration sheet

Introductory session of the use of therapeutic and prophylactic antimicrobial prescribing guidelines

Date: ______________________________, Time: ______________________________

Venue: ____________________________, Hospital: ___________________________

Participants list:

No.	Name and Surname	Age	Sex	position	Department	Year of graduation from medical school	Phone number
1							
2							
3							
4							
5							
6							
7							
8							
9							
10							
11							
12							
13							
14							
15							
16							
17							
18							
19							
20							
21							
22							
23							
24							
25							
26							
27							
28							
29							
30							

A9. Pre-prescribing guideline Survey

Pre-prescribing guideline Survey

Date: ___/___/_____, Year of graduation from medical school: ______, Speciality: ______________________, Department: ____________________Hospital: ___________________

1. **How frequently do you prescribe antimicrobial (s)?**

   ☐ Daily ☐ Nearly every day ☐ Weekly ☐ Monthly ☐ Never

2. **How often do you access any antimicrobial prescribing guidelines?**

   ☐ Daily ☐ Nearly every day ☐ Weekly ☐ Monthly ☐ Never

3. **Do you have a smart phone?** ☐ Yes ☐ No ☐ Sometimes; If answer is "No", you do not have to answer question 4, 5 and 6

4. **Do you carry your smart phone with you at work?** ☐ Yes ☐ No

5. **Do you have any antimicrobial prescribing guideline App installed on your smart phone?**

   ☐ Yes ☐ No; If answer is "No", you do not have to answer question 6

6. **How often do you access any antimicrobial prescribing mobile phone App?**

   ☐ Daily ☐ Nearly every day ☐ Weekly ☐ Monthly ☐ Never

A10. Prescriber feedback survey on paper-based prescribing guidelines

Prescriber feedback survey on paper-based prescribing guidelines

Date: ___/___/_____, Year of graduation from medical school: ______, Speciality: ____________________,

Department: ____________________Hospital: ___________________

1. Have you ever seen these paper-based antimicrobial prescribing guidelines?

   ☐ Yes ☐ No ; If answer is "No", you do not have to continue this survey

2. If yes, have you ever used it?

   ☐ Yes ☐ No ; If answer is "No", you do not have to continue this survey

   If the answer is "Yes", please continue the survey

3. Complete this table below: **Instruction**-please indicate your level of agreement with the statement listed below (by ticking the box:

	Strongly agree	Agree	Neutral	Disagree	Strongly disagree	No opinion
The name of the guidelines is appropriate						
The structure is well-organised and easy to follow						
The topics covered are relevant to my patient population						
I can find information which I look for in the guidelines easily						
Language used in the guidelines is easy to understand						
Prescribing instructions (antibiotic of choice, dose and duration) are easy to follow						
The recommended antibiotics are mostly available in my hospital						
I find these guidelines restrict my choice of antibiotics						
Extra information (*data on antibiotic susceptibility pattern, causes of infections in Laos, international recommendation*) is useful						
Extra information (*data on antibiotic susceptibility pattern, causes of infections in Laos, international recommendation*) is too detailed						
Prophylaxis section is useful						
I can get more information from provided references						
These paper-based guidelines are thick and difficult to carry for my routine work						
These paper-based guidelines encourage me to document the **indication** for antimicrobial on hospital charts						
These paper-based guidelines encourage me to document the **duration** for antimicrobial on hospital charts						
Antibiotic stewardship (AMS) section is useful and relevant to my work						
These paper-based guidelines have increased my knowledge of AMS						

4. How frequently do you prescribe antimicrobial (s)?

   ☐ Daily ☐ Nearly every day ☐ Weekly ☐ Monthly ☐ Never

5. How often do you access this provided paper-based prescribing guidelines?

   ☐ Daily ☐ Nearly every day ☐ Weekly ☐ Monthly ☐ Never

6. How often do you access other prescribing guidelines?

☐ Daily ☐ Nearly every day ☐ Weekly ☐ Monthly ☐ Never

7. How would you rate these paper-based prescribing guidelines overall?

☐ Very good and fitting with Lao context ☐ Good but need a bit more editing

☐ Moderate and need more editing ☐ Not good and need to review many sections

Please give comments in the box below:

A11. Prescriber feedback survey on mobile phone app prescribing guidelines

Prescriber feedback survey on mobile phone app prescribing guidelines

Date: ___/___/_____, Year of graduation from medical school: ______, Speciality: ______________________, Department: ____________________Hospital: ___________________

1. Have you ever seen this antimicrobial prescribing guideline mobile phone application?

   ☐ Yes ☐ No ; If answer is "No", you do not have to continue this survey

2. If yes, have you ever used it?

   ☐ Yes ☐ No ; If answer is "No", you do not have to continue this survey

   If the answer is "Yes", please continue the survey

3. Complete this table below**:** Instruction: please indicate your level of agreement with the statement listed below (by ticking the box):

	Strongly agree	Agree	Neutral	Disagree	Strongly disagree	No opinion
The structure and design are easy to follow						
I can find information which I look for more easily than from paper-based guidelines						
Language font size and colour are easy to read						
Prescribing instructions (antibiotic of choice, dose and duration) are easy to follow						
Links to extra information (*data on antibiotic susceptibility pattern, causes of infections in Laos, international recommendation*) are useful						
The offline mode makes it easy for me to access the guidelines anywhere						
The App guideline encourages me to document the **indication** for antimicrobial on hospital charts						
The App guideline encourages me to document the **duration** of antimicrobial on hospital charts						
The App guideline has increased my knowledge of antimicrobial stewardship (AMS)						
Antimicrobial stewardship training increased my knowledge of AMS						

4. How often do you prescribe antimicrobial (s)?

   ☐ Daily ☐ Nearly every day ☐ Weekly ☐ Monthly ☐ Never

5. How often do you access paper-based prescribing guidelines?

   ☐ Daily ☐ Nearly every day ☐ Weekly ☐ Monthly ☐ Never

6. Do you carry your smart phone (with this prescribing guideline App) with you at work?

   ☐ Yes ☐ No ☐ Sometimes

7. How often do you access the provided mobile phone app prescribing guideline?

☐ Daily ☐ Nearly every day ☐ Weekly ☐ Monthly ☐ Never

8. How often do you access other prescribing guidelines?

   ☐ Daily ☐ Nearly every day ☐ Weekly ☐ Monthly ☐ Never

9. How would you rate this app overall?

☐ Very good and I will continue to use it ☐ Good but needs a bit more editing and I will continue to use it

☐ Good but I prefer paper-based version ☐ moderate but I prefer paper-based version

☐ Not good and I prefer paper-based version ☐ Others ______________________________

Please give comments to improve this app:

A12. Antimicrobial stewardship (AMS) training

Training objective: to raise awareness of antibiotic good prescribing practices

Trainees: this session will be given to all hospital doctors in all study hospitals at the start of the intervention period of each paired hospital (month 4, month 8 and month 12).

Training date, time and venue: the session will take a maximum of 30 minutes with 10 minutes of discussion/questions. Training date, time and venue will be informed to all doctors one week in advance and again 3 days before starting the session. The session will be run in each hospital separately (2 hospitals on month 4, another 2 on month 8 and the last 2 hospitals on month 12) after conducting point prevalence survey (PPS) of hospital antimicrobial use, completing prescriber feedback survey and after finished introductory session of the prescribing guideline mobile phone App. Verbal consent will be asked from participants to record participant's names, age, sex, working positions, departments, years of work and phone number onto the session registration sheet.

Instructor: the principal investigator of the study will be the training instructor. Training will be given in the format of PowerPoint presentation, followed by question and discussion.

Training material: Handout of the training will be provided to all participants (the same standard session for all hospitals).

Training details: All training details already exist in the prescribing guidelines. The majority of training will be on the section of AMS and some part of the training will be from the therapeutic and prophylaxis sections. This training session will include good practice of antibiotic prescriptions, the idea and benefits of intravenous therapy to oral therapy switch, choice of prescription when patients report penicillin allergy.

Training evaluation: All participating hospitals will join AMS knowledge survey three times during the study period (1st time= at the start of the project, 2nd time= before entering to the intervention period and 3rd time= at the end of the project)

A13. Antimicrobial stewardship (AMS) training session registration sheet

Antimicrobial Stewardship (AMS) Training Session Registration Form

Date: ______________________________, Time: ______________________________
Venue: ______________________________, Hospital: ____________________________

No.	Name and Surname	Age	Sex	position	Department	Years of work (year)	Phone number
1							
2							
3							
4							
5							
6							
7							
8							
9							
10							
11							
12							
13							
14							
15							
16							
17							
18							
19							
20							
21							
22							
23							
24							
25							
26							
27							
28							
29							
30							

A14. Timelines and milestones

Activities	2020										2021												2022												2023
	3	4	5	6	7	8	9	10	11	12	1	2	3	4	5	6	7	8	9	10	11	12	1	2	3	4	5	6	7	8	9	10	11	12	1
Building App with MicroGuide and piloting the App	x	x	x	x																															
Receiving ethical approval to conduct study (minimal risk)			x	x	x																														
Meeting with Ministry of Health and Lao Infectious Society to introduce the application, formally informing the start of project and target hospitals				x	x																														
PPS on hospital antimicrobial use in both outpatient and in-patient departments in 6 hospitals						x																													
Reporting PPS results						x				x				x				x				x													
Pre-prescribing guideline survey						x																													
Antibiotic stewardship survey						x				x				x				x				x													
Introducing paper-based guidelines (paediatrics and adults) in 6 hospitals						x																													
Giving introductory session of paper-based guidelines in 6 hospitals						x																													
Prescriber feedback survey on paper-based guidelines*										x				x				x																	
Introduce the App to the first paired hospitals										x																									
Introduce the App to the second paired hospitals														x																					
Introduce the App to the third paired hospitals																		x																	
Giving introductory session of the App version of prescribing guidelines										x				x				x																	
Giving antibiotic stewardship training										x				x				x																	
Prescriber feedback survey on the App version of guidelines*														x				x				x													
Updating Ministry of health and Lao Infectious Society about the progress of the study						x					x				x				x				x												
Data cleaning																							x	x											
Analysing data																							x	x	x										
Writing thesis																							x	x	x	x	x	x							

Official DPhil start date: 29 April 2019; Transfer deadline date from PRS to DPhil is 24 April 2020; Complete confirmation DPhil status by the end of 9th term on 14th Jan 2022 (final term); Maximum submission date is 13 Jan 2023

* Prescriber feedback survey on the App will be performed in a paired hospitals/time only

	End of grant
	Maximum submission date
	Complete confirmation DPhil status
