# Supplementary material for: Adherence to smartphone-delivered antimicrobial prescribing guidelines in hospitals in Lao PDR: A stepped-wedge cluster randomized controlled trial
Source: PLoS Med. 2026 Aug 27;23(8):e1005205. doi: 10.1371/journal.pmed.1005205 (PMC13521649; doi:10.1371/journal.pmed.1005205)
Supplement: Supplementary file 2 [file pmed.1005205.s011.docx]

**S1 Table**: Stepped wedge cluster randomized controlled trial (SW-CRCT) study sites

| **Study sites** | **Mahosot (MS)** | **Vientiane Province (VTP)** | **Xiengkhuang (XK)** | **Luang Namtha (LNT)** | **Salavan (SV)** | **Savannakhet (SVK)** |
| --- | --- | --- | --- | --- | --- | --- |
| Region | Centre | Centre | North | North | South | South |
| Location (above mean sea level) | 17^o^95’N 102^o^ 61’E, 268m | 18^o^ 47’N 102^o^48’E 497m | 19°27’N 103°10’E, 1,150m | 21°00’N 101°24’ E; 570m | 15°43’N 106°25’ E; 184m | 16°55’N 104° 74’E; 726 m |
| Type of hospital | Central referral | Provincial | Provincial | Provincial | Provincial | Regional |
| Number of beds | 450 | 100 | 150 | 50 | 70 | 200 |
| Number of staff | 968**^a^** | 211**^b^** | 305**^c^** | 117**^b^** | 191**^b^** | 293**^c^** |
| Physician | 222 | 30 | 66 | 18 | 30 | 60 |
| Number of wards and OPD | 18 wards, one main OPD and small number of ward-based OPDs | Six wards and one OPD | 11 wards, one main OPD and small number of ward-based OPDs | Six wards and one main OPD | Seven wards and ward-based OPDs | Nine wards, one main OPD and small number of ward-based OPDs |

**Note: ^a^** data from 2020, **^b^** data from 2018, **^c^** data from 2019, **N**= North, **E**= East, **OPD**= Outpatient Department
