# Supplementary material for: Adherence to smartphone-delivered antimicrobial prescribing guidelines in hospitals in Lao PDR: A stepped-wedge cluster randomized controlled trial
Source: PLoS Med. 2026 Aug 27;23(8):e1005205. doi: 10.1371/journal.pmed.1005205 (PMC13521649; doi:10.1371/journal.pmed.1005205)
Supplement: Supplementary file 3 [file pmed.1005205.s012.docx]

**S2 Table:** Total number of prescribers with access to paper-based and app-based antimicrobial prescribing guidelines

| **Hospital** | **Prescribers with access to paper-based guidelines,**  **n (%; [95% CI])** | **Prescribers with access to app-based guidelines,**  **n (%; [95%CI])** |
| --- | --- | --- |
| Xiengkhuang | 51/53 (96.2; [87.0,99.5]) | 49/60 (76.6; [64.3,86.3]) |
| Luang Namtha | 27/35 (77.1; [59.9,89.6]) | 24/33 (72.7; [54.5,86.7]) |
| Salavan | 32/49 (65.3; [50.4,78.3]) | 36/44 (81.8; [67.3,91.8]) |
| Savannakhet | 70/83 (84.3; [74.7,91.4]) | 85/96 (88.5; [80.4,94.1]) |
| Vientiane Province | 40/40 (100) | 28/36 (77.8; [60.8,89.9]) |
| Mahosot | 163/247 (66.0; [59.7,71.9]) | 140/208 (67.3; [60.5,73.6]) |
| Total | 383/507 (75.5; [71.6,79.2]) | 362/477 (75.9; [71.8,79.7]) |
