# Supplementary material for: Adherence to smartphone-delivered antimicrobial prescribing guidelines in hospitals in Lao PDR: A stepped-wedge cluster randomized controlled trial
Source: PLoS Med. 2026 Aug 27;23(8):e1005205. doi: 10.1371/journal.pmed.1005205 (PMC13521649; doi:10.1371/journal.pmed.1005205)
Supplement: Supplementary file 4 [file pmed.1005205.s013.docx]

**S3 Table:** Specialty distribution of prescribers with access to prescribing guidelines included in the study

| **Specialties** | **Prescribers with access to paper-based guidelines,**  **n= 370 (%)** | **Prescribers with access to app-based guidelines,**  **n= 344 (%)** |
| --- | --- | --- |
| Medicine | 81 (21.9) | 77 (22.4) |
| Obstetrics and Gynecology | 52 (14.1) | 45 (13.1) |
| Surgery | 55 (14.9) | 44 (12.8) |
| Pediatrics | 26 (7.0) | 30 (8.7) |
| Intentive Care Unit | 39 (10 | 29 (8.4) |
| Ear-Nose-Throat | 21 (5.7) | 29 (8.4) |
| Emergency Medicine | 30 (8.1) | 26 (7.6) |
| Anesthesia | 5 (1.3) | 14 (4.1) |
| Outpatient Departments | 7 (1.9) | 6 (1.7) |
| Other | 54 (14.6) | 44 (12.8) |
