# Supplementary material for: Adherence to smartphone-delivered antimicrobial prescribing guidelines in hospitals in Lao PDR: A stepped-wedge cluster randomized controlled trial
Source: PLoS Med. 2026 Aug 27;23(8):e1005205. doi: 10.1371/journal.pmed.1005205 (PMC13521649; doi:10.1371/journal.pmed.1005205)
Supplement: Supplementary file 5 [file pmed.1005205.s014.docx]

# **S4 Table:** Number of screened hospitalized patient (IPD) records and outpatient (OPD) prescriptions in participating hospitals

| Hospital | Period 1 | | Period 2 | | Period 3 | | Period 4 | | Period 5 | |
| --- | --- | --- | --- | --- | --- | --- | --- | --- | --- | --- |
|  | IPD | OPD | IPD | OPD | IPD | OPD | IPD | OPD | IPD | OPD |
| Xiengkhuang | 111 | 274 | 110 | 438 | 34 | 266 | 45 | 321 | 123 | 343 |
| Luang Namtha | 56 | 79 | 56 | 288 | 8 | 92 | 44 | 280 | 48 | 188 |
| Salavan | 86 | 464 | 89 | 641 | 62 | 376 | 74 | 406 | 130 | 565 |
| Savannakhet | 218 | 378 | 197 | 340 | 126 | 179 | 148 | 230 | 170 | 415 |
| Vientiane Province | 94 | 750 | 117 | 493 | 56 | 196 | 44 | 348 | 96 | 576 |
| Mahosot | 192 | 1,131 | 167 | 898 | 83 | 582 | 158 | 818 | 225 | 1,356 |
| Total | 757 | 3,076 | 736 | 3,098 | 369 | 1,691 | 513 | 2,403 | 792 | 3,443 |

Note: **Period 1** = Pre-guideline, **Period 2** = Month-6 after distribution of paper-based guidelines (all hospitals), **Period 3** = First survey after intervention implementation in Xiengkhuang and Luang Namtha or month-11 after paper-based guidelines introduction, **Period 4** = Second survey post-intervention introduction for Xiengkhuang and Luang Namtha, first survey post-intervention for Salavan and Savannakhet, or month-17 after paper-based guidelines introduction, **Period 5** = final survey after all clusters had access to the intervention or month-22 after paper-based guidelines introduction.
