# Supplementary material for: Adherence to smartphone-delivered antimicrobial prescribing guidelines in hospitals in Lao PDR: A stepped-wedge cluster randomized controlled trial
Source: PLoS Med. 2026 Aug 27;23(8):e1005205. doi: 10.1371/journal.pmed.1005205 (PMC13521649; doi:10.1371/journal.pmed.1005205)
Supplement: Supplementary file 6 [file pmed.1005205.s015.docx]

# **S5 Table:** List of antimicrobial prescriptions in six hospitals in each survey period

| Antimicrobial class | Antimicrobial agent | Period 1 | | Period 2 | | Period 3 | | Period 4 | | Period 5 | | Total | |
| --- | --- | --- | --- | --- | --- | --- | --- | --- | --- | --- | --- | --- | --- |
|  |  | IPD  (n= 852) | OPD  (n= 926) | IPD  (n= 808) | OPD  (n= 748) | IPD  (n= 496) | OPD  (n= 515) | IPD  (n= 607) | OPD  (n= 809) | IPD  (n= 798) | OPD  (n= 907) | IPD  (n= 3,561) | OPD  (n= 3,905) |
| Antibiotics |  | 842 (98.8) | 826 (89.2) | 786 (97.3) | 646 (86.4) | 475 (95.8) | 476 (92.4) | 584 (96.2) | 731 (90.4) | 746 (93.5) | 847 (93.4) | 3,433 (96.4) | 3,526 (90.3) |
| WHO AWaRe |  |  |  |  |  |  |  |  |  |  |  |  |  |
| Access |  | 423 (50.2) | 561 (67.9) | 401 (51.0) | 431 (66.7) | 222 (46.7) | 287 (60.3) | 256 (44.0) | 496 (67.8) | 326 (44.0) | 559 (66.0) | 1,628 (47.4) | 2,334 (66.2) |
| Watch |  | 416 (49.4) | 265 (32.1) | 384 (48.9) | 215 (33.3) | 252 (53.1) | 189 (39.7) | 328 (56.0) | 235 (32.2) | 420 (56.0) | 288 (34.0) | 1,800 (52.4) | 1,192 (33.8) |
| Reserve |  | 0 | 0 | 0 | 0 | 0 | 0 | 0 | 0 | 0 | 0 | 0 | 0 |
| Not recommended |  | 3 (0.4) | 0 | 1 (0.1) | 0 | 1 (0.2) | 0 | 0 | 0 | 0 | 0 | 5 (0.2) | 0 |
| Aminoglycosides |  | 85 (10) | 0 | 70 (8.7) | 0 | 39 (7.9) | 0 | 68 (11.2) | 0 | 61 (7.6) | 1 (0.1) | 323 (9.1) | 1 (0.03) |
|  | Amikacin | 1 (0.1) | 0 | 1 (0.1) | 0 | 0 | 0 | 0 | 0 | 3 (0.4) | 0 | 5 (0.1) | 0 |
|  | Gentamicin | 84 (9.8) | 0 | 69 (8.5) | 0 | 39 (7.9) | 0 | 68 (11.2) | 0 | 58 (7.3) | 1 (0.1) | 318 (9.3) | 1 (0.03) |
| BL/BLIs |  | 4 (0.5) | 33 (3.6) | 8 (1.0) | 48 (6.4) | 6 (1.2) | 25 (4.8) | 3 (0.5) | 30 (3.7) | 15 (1.9) | 44 (4.9) | 36 (1.0) | 180 (4.6) |
|  | Amoxicillin/ clavulanic acid | 1 (0.1) | 33 (3.6) | 7 (0.9) | 48 (6.4) | 5 (1.0) | 25 (4.8) | 3 (0.5) | 30 (3.7) | 15 (1.9) | 44 (4.8) | 31 (0.9) | 180 (4.6) |
|  | Ceftriaxone/ sulbactam | 3 (0.3) | 0 | 1 (0.1) | 0 | 1 (0.2) | 0 | 0 | 0 | 0 | 0 | 5 (0.1) | 0 |
| Carbapenems | Meropenem | 10 (1.2) | 0 | 19 (2.3) | 0 | 10 (2.0) | 0 | 13 (2.1) | 0 | 21 (2.6) | 0 | 73 (2.1) | 0 |
| Cephalosporins |  | 388 (45.5) | 242 (26.1) | 357 (44.2) | 139 (18.6) | 224 (45.2) | 103 (20.0) | 304 (50.1) | 211 (26.1) | 356 (44.6) | 228 (25.1) | 1,629 (45.7) | 923 (23.6) |
| 1^st^GC | Cefalexin | 15 (1.8) | 189 (20.4) | 21 (2.6) | 109 (14.6) | 4 (0.8) | 72 (14.0) | 15 (2.5) | 180 (22.2) | 16 (2.0) | 184 (20.3) | 71 (2.1) | 734 (18.8) |
| 2^nd^GC | Cefuroxime | 0 | 0 | 0 | 0 | 0 | 0 | 0 | 0 | 2 (0.2) | 0 | 2 (0.06) | 0 |
| 3^rd^GC | Cefixime | 0 | 53 (5.7) | 3 (0.4) | 28 (3.7) | 0 | 31 (6.0) | 3 (0.5) | 31 (3.8) | 1 (0.1) | 42 (4.6) | 7 (0.2) | 185 (4.7) |
|  | Cefotaxime | 14 (1.6) | 0 | 9 (1.1) | 0 | 7 (1.4) | 0 | 12 (2.0) | 0 | 11 (1.4) | 0 | 53 (1.5) | 0 |
|  | Ceftazidime | 8 (0.9) | 0 | 12 (1.5) | 0 | 13 (2.6) | 0 | 13 (2.1) | 0 | 24 (3.0) | 0 | 70 (2.0) | 0 |
|  | Ceftriaxone | 354 (41.5) | 0 | 312 (38.6) | 2 (0.3) | 200 (40.3) | 0 | 261 (43.0) | 0 | 302 (37.8) | 2 (0.2) | 1,426 (41.5) | 4 (0.1) |
| Fluoroquinolones |  | 17 (2.0) | 80 (8.6) | 15 (1.9) | 84 (11.2) | 8 (1.6) | 63 (12.2) | 16 (2.6) | 82 (10.1) | 34 (4.3) | 86 (9.5) | 90 (2.5) | 395 (10.1) |
|  | Ciprofloxacin | 8 (0.9) | 8 (0.9) | 8 (1.0) | 22 (2.9) | 6 (1.2) | 11 (2.1) | 9 (1.5) | 21 (2.6) | 16 (2.0) | 20 (2.2) | 47 (1.4) | 82 (2.1) |
|  | Levofloxacin | 2 (0.2) | 0 | 0 | 1 (0.1) | 0 | 0 | 3 (0.5) | 0 | 9 (1.1) | 0 | 14 (0.4) | 1 (0.03) |
|  | Norfloxacin | 2 (0.2) | 30 (3.2) | 2 (0.2) | 19 (2.5) | 1 (0.2) | 29 (5.6) | 1 (0.2) | 20 (2.5) | 5 (0.6) | 31 (3.4) | 11 (0.3) | 129 (3.3) |
|  | Ofloxacin | 5 (0.6) | 42 (4.5) | 5 (0.6) | 42 (5.6) | 1 (0.2) | 23 (4.5) | 3 (0.5) | 41 (5.1) | 4 (0.5) | 35 (3.9) | 18 (0.5) | 183 (4.7) |
| Glycopeptide | Vancomycin | 0 | 0 | 0 | 0 | 0 | 0 | 0 | 0 | 2 (0.2) | 0 | 2 (0.06) | 0 |
| Lincosamides |  | 1 (0.1) | 1 (0.1) | 0 | 3 (0.4) | 0 | 0 | 0 | 0 | 0 | 0 | 1 (0.03) | 4 (0.1) |
|  | Clindamycin | 1 (0.1) | 0 | 0 | 0 | 0 | 0 | 0 | 0 | 0 | 0 | 1 (0.03) | 0 |
|  | Lincomycin | 0 | 1 (0.1) | 0 | 3 (0.4) | 0 | 0 | 0 | 0 | 0 | 0 | 0 | 4 (0.1) |
| Macrolides |  | 16 (1.9) | 131 (14.2) | 14 (1.7) | 99 (13.2) | 14 (2.8) | 95 (18.5) | 10 (1.7) | 121 (14.9) | 23 (2.9) | 158 (17.4) | 77 (2.2) | 604 (15.5) |
|  | Azithromycin | 11 (1.3) | 97 (10.5) | 13 (1.6) | 73 (9.8) | 12 (2.4) | 71 (13.8) | 8 (1.3) | 86 (10.6) | 20 (2.5) | 122 (13.5) | 64 (1.9) | 449 (11.5) |
|  | Clarithromycin | 1 (0.1) | 5 (0.5) | 1 (0.1) | 4 (0.5) | 2 (0.4) | 5 (1.0) | 1 (0.2) | 7 (0.9) | 1 (0.1) | 11 (1.2) | 6 (0.2) | 32 (0.8) |
|  | Erythromycin | 4 (0.5) | 26 (2.8) | 0 | 21 (2.8) | 0 | 18 (3.5) | 1 (0.2) | 28 (3.5) | 2 (0.2) | 24 (2.6) | 7 (0.2) | 117 (3.0) |
|  | Roxythromycin | 0 | 3 (0.3) | 0 | 1 (0.1) | 0 | 1 (0.2) | 0 | 0 | 0 | 1 (0.1) | 0 | 6 (0.1) |
| Nitroimidazole | Metronidazole | 164 (19.2) | 52 (5.6) | 167 (21) | 53 (7.1) | 113 (22.8) | 37 (7.2) | 105 (17.3) | 47 (5.8) | 107 (13.4) | 40 (4.4) | 656 (19.1) | 229 (5.9) |
| Penicillins |  | 141 (16.6) | 251 (27.1) | 124 (15.4) | 187 (25) | 50 (10.1) | 133 (25.8) | 53 (8.7) | 214 (26.5) | 111 (13.9) | 236 (26.0) | 479 (13.5) | 1,021 (26.2) |
|  | Amoxicillin | 58 (6.8) | 143 (15.4) | 44 (5.4) | 92 (12.3) | 16 (3.2) | 69 (13.4) | 14 (2.3) | 137 (16.9) | 43 (5.4) | 190 (20.9) | 175 (5.1) | 631 (16.2) |
|  | Ampicillin | 58 (6.8) | 22 (2.4) | 53 (6.5) | 21 (2.8) | 22 (4.4) | 21 (4.1) | 32 (5.3) | 30 (3.7) | 49 (6.1) | 4 (0.4) | 214 (6.2) | 98 (2.5) |
|  | Cloxacillin | 19 (2.2) | 36 (3.9) | 22 (2.7) | 52 (6.9) | 12 (2.4) | 32 (6.2) | 7 (1.1) | 28 (3.5) | 18 (2.2) | 26 (2.9) | 78 (2.3) | 174 (4.5) |
|  | Penicillin G | 4 (0.5) | 0 | 4 (0.5) | 0 | 0 | 0 | 0 | 0 | 0 | 4 (0.4) | 8 (0.2) | 4 (0.1) |
|  | Penicillin V | 2 (0.2) | 50 (5.4) | 1 (0.1) | 22 (2.9) | 0 | 11 (2.1) | 0 | 19 (2.3) | 1 (0.1) | 12 (1.3) | 4 (0.1) | 114 (2.9) |
| Sulfonamides | Co-trimoxazole | 7 (0.8) | 6 (0.6) | 5 (0.6) | 8 (1.1) | 5 (1.0) | 4 (0.8) | 3 (0.5) | 5 (0.6) | 8 (1.0) | 6 (0.7) | 28 (0.8) | 29 (0.7) |
| Tetracyclines | Doxycycline | 9 (1.1) | 30 (3.2) | 7 (0.9) | 25 (3.3) | 6 (1.2) | 16 (3.1) | 9 (1.5) | 21 (2.6) | 8 (1.0) | 48 (5.3) | 39 (1.1) | 140 (3.6) |
| Antifungals |  | 3 (0.4) | 12 (1.3) | 2 (0.3) | 18 (2.4) | 9 (1.8) | 4 (0.8) | 2 (0.3) | 16 (2.0) | 8 (1.0) | 11 (1.2) | 24 (0.7) | 61 (1.6) |
|  | Amphotericin B | 0 | 0 | 0 | 0 | 1 (0.2) | 0 | 1 (0.2) | 0 | 1 (0.1) | 0 | 3 (0.1) | 0 |
|  | Fluconazole | 3 (0.3) | 0 | 1 (0.1) | 0 | 7 (1.4) | 0 | 1 (0.2) | 2 (0.3) | 7 (0.9) | 2 (0.2) | 19 (0.5) | 4 (0.1) |
|  | Ketoconazole | 0 | 9 (1.0) | 0 | 14 (1.9) | 0 | 2 (0.4) | 0 | 5 (0.6) | 0 | 5 (0.5) | 0 | 35 (0.9) |
|  | Nystatin | 0 | 3 (0.3) | 1 (0.1) | 4 (0.5) | 1 (0.2) | 2 (0.4) | 0 | 9 (1.1) | 0 | 4 (0.4) | 2 (0.1) | 22 (0.5) |
| Antiparasitics |  | 2 (0.2) | 84 (9.1) | 7 (0.9) | 83 (11.1) | 0 | 31 (6.0) | 1 (0.2) | 60 (7.4) | 4 (0.5) | 42 (4.6) | 14 (0.4) | 300 (7.7) |
|  | Albendazole | 1 (0.1) | 79 (8.5) | 4 (0.5) | 63 (8.4) | 0 | 29 (5.6) | 1 (0.2) | 55 (6.8) | 4 (0.5) | 36 (4.0) | 10 (0.3) | 262 (6.7) |
|  | Mebendazole | 1 (0.1) | 3 (0.3) | 3 (0.4) | 18 (2.4) | 0 | 1 (0.2) | 0 | 4 (0.5) | 0 | 5 (0.5) | 4 (0.1) | 31 (0.8) |
|  | Praziquantel | 0 | 2 (0.2) | 0 | 2 (0.3) | 0 | 1 (0.2) | 0 | 1 (0.1) | 0 | 1 (0.1) | 0 | 7 (0.2) |
| Antiretrovirals |  | 0 | 0 | 3 (0.4) | 0 | 2 (0.4) | 0 | 1 (0.2) | 0 | 0 | 1 (0.1) | 6 (0.2) | 1 (0.03) |
|  | ABC and 3TC | 0 | 0 | 0 | 0 | 0 | 0 | 1 (0.2) | 0 | 0 | 0 | 1 (0.03) | 0 |
|  | ABC | 0 | 0 | 0 | 0 | 1 (0.2) | 0 | 0 | 0 | 0 | 1 (0.1) | 1 (0.03) | 1 (0.03) |
|  | DTG | 0 | 0 | 1 (0.1) | 0 | 0 | 0 | 1 (0·2) | 0 | 0 | 0 | 2 (0.1) | 0 |
|  | 3TC and AZT | 0 | 0 | 1 (0.1) | 0 | 0 | 0 | 0 | 0 | 0 | 0 | 1 (0.03) | 0 |
|  | TDF, 3TC and DTG | 0 | 0 | 1 (0.1) | 0 | 1 (0.2) | 0 | 0 | 0 | 0 | 0 | 2 (0.1) | 0 |
| Antivirals |  | 0 | 4 (0.4) | 2 (0.3) | 1 (0.1) | 0 | 4 (0.8) | 1 (0.3) | 2 (0.3) | 3 (0.4) | 6 (0.7) | 6 (0.2) | 17 (0.4) |
|  | Acyclovir | 0 | 4 (0.4) | 2 (0.2) | 1 (0.1) | 0 | 4 (0.8) | 1 (0.2) | 2 (0.3) | 2 (0.5) | 5 (0.5) | 5 (0.1) | 16 (0.4) |
|  | Molnupiravir | 0 | 0 | 0 | 0 | 0 | 0 | 0 | 0 | 1 (0.1) | 0 | 1 (0.03) | 0 |
|  | Ombitasvir, Paritaprevir and Ritonavir | 0 | 0 | 0 | 0 | 0 | 0 | 0 | 0 | 0 | 1 (0.1) | 0 | 1 (0.03) |
| Antituberculosis |  | 5 (0.6) | 0 | 8 (1.0) | 0 | 10 (2.0) | 0 | 17 (2.8) | 0 | 37 (4.6) | 0 | 77 (2.2) | 0 |
|  | Bedaquiline | 0 | 0 | 0 | 0 | 0 | 0 | 2 (0.3) | 0 | 8 (1.0) | 0 | 10 (0.3) | 0 |
|  | Clofazimine | 0 | 0 | 0 | 0 | 0 | 0 | 3 (0.5) | 0 | 8 (1.0) | 0 | 11 (0.3) | 0 |
|  | Ethambutol | 0 | 0 | 0 | 0 | 0 | 0 | 1 (0.2) | 0 | 0 | 0 | 1 (0.03) | 0 |
|  | Linezolid | 0 | 0 | 0 | 0 | 0 | 0 | 2 (0.3) | 0 | 4 (0.5) | 0 | 6 (0.2) | 0 |
|  | Pyrazinamide | 0 | 0 | 0 | 0 | 0 | 0 | 3 (0.5) | 0 | 8 (1.0) | 0 | 11 (0.3) | 0 |
|  | Rifampicin | 0 | 0 | 1 (0.1) | 0 | 2 (0.4) | 0 | 1 (0.2) | 0 | 0 | 0 | 4 (0.1) | 0 |
|  | Rifampicin, isoniazid | 2 (0.2) | 0 | 0 | 0 | 0 | 0 | 0 | 0 | 1 (0.1) | 0 | 3 (0.08) | 0 |
|  | 4FDC | 3 (0.3) | 0 | 7 (0.9) | 0 | 8 (1.6) | 0 | 5 (0.8) | 0 | 8 (1.0) | 0 | 31 (0.9) | 0 |

**Note: BL/BLIs** = Beta-lactam/Beta-lactamase inhibitors, **GC** = Generation Cephalosporin, **ABC** = Abacavir, **DTG** = Dolutegravir, **3TC** = Lamivudine, **AZT** = Zidovudine, **TDF** = Tenofovir Disoproxil fumarate, **4FDC** (fixed-dose combination) = Rifampicin, pyrazinamide, ethambutol, isoniazid.
