# Supplementary material for: Adherence to smartphone-delivered antimicrobial prescribing guidelines in hospitals in Lao PDR: A stepped-wedge cluster randomized controlled trial
Source: PLoS Med. 2026 Aug 27;23(8):e1005205. doi: 10.1371/journal.pmed.1005205 (PMC13521649; doi:10.1371/journal.pmed.1005205)
Supplement: Supplementary file 7 [file pmed.1005205.s016.docx]

# **S6 Table:** Antimicrobial prescriptions categorised by WHO AWaRe (Access, Watch, Reserve) group among hospitalized patients

| **Antibiotics** | **Pre-guideline; n (%)** | **Paper-based group, n (%)** | **App-based group; n (%)** |
| --- | --- | --- | --- |
| Access | 423 (50.2) | 694 (48.0) | 511 (44.6) |
| Metronidazole | 164 (38.8) | 314 (45.2) | 178 (34.8) |
| Gentamicin | 84 (19.9) | 133 (19.2) | 101 (19.8) |
| Amoxicillin | 58 (13.7) | 84 (12.1) | 61 (11.9) |
| Ampicillin | 58 (13.7) | 56 (8.1) | 72 (14.1) |
| Cloxacillin | 19 (4.5) | 33 (4.8) | 26 (5.1) |
| Cefalexin | 15 (3.6) | 28 (4.3) | 28 (5.5) |
| Doxycycline | 9 (2.1) | 14 (2.1) | 16 (3.1) |
| Co-trimoxazole | 7 (1.7) | 11 (1.6) | 10 (2) |
| Penicillin G | 4 (5) | 4 (0.6) | 0 |
| Penicillin V | 2 (0.5) | 1 (0.1) | 1 (0.2) |
| Amikacin | 1 (0.2) | 1 (0.1) | 3 (0.6) |
| Amoxicillin/clavulanic acid | 1 (0.2) | 15 (2.2) | 15 (2.9) |
| Clindamycin | 1 (0.2) | 0 | 0 |
| Watch | 416 (49.4) | 750 (51.9) | 634 (55.4) |
| Ceftriaxone | 351 (84.4) | 610 (81.3) | 465 (73.3) |
| Cefotaxime | 14 (3.4) | 21 (2.8) | 18 (2.8) |
| Azithromycin | 11 (2.6) | 28 (3.7) | 25 (3.9) |
| Meropenem | 10 (2.4) | 33 (4.4) | 30 (4.7) |
| Ceftazidime | 8 (1.9) | 27 (3.6) | 35 (5.5) |
| Ciprofloxacin | 8 (1.9) | 13 (1.7) | 26 (4.1) |
| Ofloxacin | 5 (1.2) | 8 (1.1) | 5 (0.8) |
| Erythromycin | 4 (1.0) | 0 | 3 (0.5) |
| Levofloxacin | 2 (0.5) | 0 | 12 (1.9) |
| Norfloxacin | 2 (0.5) | 3 (0.4) | 6 (0.9) |
| Clarithromycin | 2 (0.5) | 2 (0.3) | 3 (0.5) |
| Cefixime | 0 | 5 (0.7) | 2 (0.3) |
| Cefuroxime | 0 | 0 | 2 (0.3) |
| Vancomycin | 0 | 0 | 2 (0.3) |
| Not recommended | 3 (0.4) | 2 (0.1) | 0 |
| Ceftriaxone/sulbactam | 3 (100) | 2 (100) | 0 |
