# Supplementary material for: Adherence to smartphone-delivered antimicrobial prescribing guidelines in hospitals in Lao PDR: A stepped-wedge cluster randomized controlled trial
Source: PLoS Med. 2026 Aug 27;23(8):e1005205. doi: 10.1371/journal.pmed.1005205 (PMC13521649; doi:10.1371/journal.pmed.1005205)
Supplement: Supplementary file 8 [file pmed.1005205.s017.docx]

**S7 Table**: Comparison of antimicrobial use between app-based and paper-based guidelines groups among hospitalized patients in six hospitals

| **Variables** | **Adjusted-OR [95% CI]** | ***p*-value** |
| --- | --- | --- |
| Guideline delivery method: |  |  |
| Paper-based (PP-G) | 1 |  |
| App-based (App-G) | 0.82 [0.61,1.12] | 0.222 |
| Sequence of intervention: |  |  |
| Sequence 3 (Mahosot+Vientiane province) | 1 |  |
| Sequence 2 (Salavan+Savannakhet) | 1.91 [1.34,2.73] | <0.0001 |
| Sequence 1 (Xiengkhuang+Luang Namtha) | 0.69 [0.47,1.01] | 0.060 |
| Department: |  |  |
| Surgery | 1 |  |
| Obstetrics and Gynecology | 0.28 [0.19,0.43] | <0.0001 |
| Medicine | 0.11 [0.08,0.16] | <0.0001 |
| Pediatrics | 0.22 [0.14,0.33] | <0.0001 |
| Intensive Care Unit | 0.36 [0.22,0.57] | <0.0001 |
| Other | 0.17 [0.11,0.26] | <0.0001 |

**Note**: **PP-G** = group with paper-based version of the antimicrobial prescribing guidelines, **App-G** = group with application-based version of the antimicrobial prescribing guidelines and antimicrobial stewardship training, **Other** = including VIP departments (a ward with enhanced service (e,g., private rooms) for patients who can afford to pay, ENT-EYE (Ear, Nose, Throat and Eyes), **aOR** = adjusted odds ratio, **Sequence 1** (Xiengkhuang+Luang Namtha) = started at month-6 after PP-G implementation, **Sequence 2** (Savannakhet+Salavan) = started at month-11 after PP-G, **Sequence 3** (Mahosot+Vientiane Province) = started at month-17 after PP-G, **P-values** were derived from the Wald Chi-Square test. The model was adjusted for sequence and hospital departments. Indication was the unit of analysis, with random effects for patient and hospital-time.
