# Supplementary material for: Adherence to smartphone-delivered antimicrobial prescribing guidelines in hospitals in Lao PDR: A stepped-wedge cluster randomized controlled trial
Source: PLoS Med. 2026 Aug 27;23(8):e1005205. doi: 10.1371/journal.pmed.1005205 (PMC13521649; doi:10.1371/journal.pmed.1005205)
Supplement: Supplementary file 9 [file pmed.1005205.s018.docx]

# **S8 Table**: Overall antibiotic defined daily doses (DDD) per 100 patient days in adult hospitalized patients in six hospitals before and after antimicrobial prescribing guidelines implementation

| Mahosot | 97.9 | 98.2 | 136.5 | 92.7 | 102.7 | 102.2 |
| --- | --- | --- | --- | --- | --- | --- |
| Vientiane Province | 122.0 | 135.3 | 139.3 | 127.6 | 90.1 | 122.6 |
| Savannakhet | 100.7 | 107.2 | 128.1 | 133.0 | 107.2 | 113.3 |
| Salavan | 114.5 | 95.0 | 131.5 | 101.9 | 80.2 | 101.6 |
| Luang Namtha | 100.6 | 86.8 | 233.4 | 98.0 | 77.8 | 94.9 |
| Xiengkhuang | 93.3 | 111.2 | 74.1 | 75.6 | 59.8 | 84.9 |
| Total | 103.4 | 107.4 | 129.3 | 107.3 | 91.0 | 105.3 |
|  | Period 1 | Period 2 | Period 3 | Period 4 | Period 5 | Total |

**Note: white area** = group with no antimicrobial prescribing guidelines, **green area** = group with paper-based version of prescribing guidelines (PP-G), **blue area** = group with application-based version of prescribing guidelines and antimicrobial stewardship training (App-G). **Period 1** = Pre-guideline, **Period 2** = Month-6 after PP-G implementation (all hospitals), **Period 3** = First survey after App-G implementation in Xiengkhuang and Luang Namtha or month-11 after PP-G introduction, **Period 4** = Second survey post-App-G for Xiengkhuang and Luang Namtha, first survey post-App-G for Salavan and Savannakhet, or month-17 after PP-G introduction, **Period 5** = final survey after all clusters had App-G access or month-22 after PP-G introduction.
