# Supplementary material for: Adherence to smartphone-delivered antimicrobial prescribing guidelines in hospitals in Lao PDR: A stepped-wedge cluster randomized controlled trial
Source: PLoS Med. 2026 Aug 27;23(8):e1005205. doi: 10.1371/journal.pmed.1005205 (PMC13521649; doi:10.1371/journal.pmed.1005205)
Supplement: Supplementary file 10 [file pmed.1005205.s019.docx]

# **S9 Table:** Comparison of the use of intravenous antimicrobials between PP-G and App-G groups among hospitalized patients

| **Characteristics** | **aOR (95% CI)** | ***p*-value** |
| --- | --- | --- |
| **Guideline delivery method:** |  |  |
| PP-G | 1 |  |
| App-G | 0.77 [0.5,1.0] | 0.103 |
| **Sequence of intervention** |  |  |
| Sequence 3 (MS+VTP) | 1 |  |
| Sequence 2 (SV+SVK) | 0.85 [0.6,1.2] | 0.384 |
| Sequence 1 (XK+LNT) | 0.58 [0.4,0.9] | 0.023 |
| **Age:** |  |  |
| ≤1 year | 1 |  |
| >1 to ≤5 years | 0.23 [0.1,0.7] | 0.015 |
| >5 to ≤14 years | 0.26 [0.1,0.9] | 0.034 |
| ≥15 years | 0.25 [0.1,0.8] | 0.016 |
| **Department**: |  |  |
| Surgery | 1 |  |
| Obstetrics and Gynecology | 0.10 [0.05,0.2] | <0.001 |
| Medicine | 0.17 [0.1,0.3] | <0.001 |
| Pediatrics | 0.73 [0.2,2.0] | 0.553 |
| Intensive Care Unit | 0.98 [0.4,2.2] | 0.971 |
| Other | 0.34 [0.2,0.6] | 0.001 |

**Note: PP-G** = group with paper-based version of the antimicrobial prescribing guidelines, **App-G** = group with application-based version of the antimicrobial prescribing guidelines and antimicrobial stewardship training, **Other** (department) = including VIP departments, ENT-EYE (Ear, Nose, Throat and Eyes), **aOR** = adjusted odds ratio, **XK** = Xiengkhuang, **LNT** = Luang Namtha, **SV**=Salavan, **SVK** = Savannakhet, **VTP** = Vientiane Provincial, **MS** = Mahosot**, Sequence 1** (Xiengkhuang+Luang Namtha) = started at month-6 after PP-G implementation, **Sequence 2** (Salavan+Savannakhet) = started at month-11 after PP-G, **Sequence 3** (Mahosot+Vientiane Province) = started at month-17 after PP-G, ***p*-values** were derived from the Wald Chi-Square test.
