# Supplementary material for: Adherence to smartphone-delivered antimicrobial prescribing guidelines in hospitals in Lao PDR: A stepped-wedge cluster randomized controlled trial
Source: PLoS Med. 2026 Aug 27;23(8):e1005205. doi: 10.1371/journal.pmed.1005205 (PMC13521649; doi:10.1371/journal.pmed.1005205)
Supplement: Supplementary file 11 [file pmed.1005205.s020.docx]

**S10 Table**: Antimicrobial prescriptions categorised by WHO AWaRe (Access, Watch, Reserve) group among outpatients

| **Antibiotics** | **Pre-guideline; n (%)** | **PP-G, n (%)** | **App-G, n (%)** |
| --- | --- | --- | --- |
| Access | 561 (67.9) | 743 (62.1) | 1,030 (68.5) |
| Cefalexin | 189 (33.7) | 195 (26.2) | 350 (34) |
| Amoxicillin | 143 (25.5) | 161 (21.7) | 327 (31.8) |
| Metronidazole | 52 (9.3) | 99 (13.3) | 78 (7.6) |
| Penicillin V | 50 (8.9) | 26 (3.5) | 38 (3.7) |
| Cloxacillin | 36 (6.4) | 81 (10.9) | 57 (5.5) |
| Amoxicillin/clavulanic acid | 33 (5.9) | 90 (12.1) | 57 (5.5) |
| Doxycycline | 30 (5.4) | 41 (5.5) | 69 (6.7) |
| Ampicillin | 22 (3.9) | 39 (5.3) | 37 (3.6) |
| Co-trimoxazole | 6 (1.1) | 11 (1.5) | 12 (1.2) |
| Penicillin G | 0 | 0 | 4 (0.4) |
| Gentamicin | 0 | 0 | 1 (0.1) |
| Watch | 265 (32.1) | 454 (37.9) | 473 (31.5) |
| Azithromycin | 97 (36.6) | 171 (37.7) | 181 (38.3) |
| Cefixime | 53 (20.0) | 68 (15) | 64 (13.5) |
| Ofloxacin | 42 (15.9) | 79 (17.4) | 62 (13.1) |
| Norfloxacin | 30 (11.3) | 37 (8.2) | 62 (13.1) |
| Erythromycin | 26 (9.8) | 38 (8.4) | 53 (11.2) |
| Ciprofloxacin | 8 (3.0) | 40 (8.8) | 34 (7.2) |
| Clarithromycin | 5 (1.9) | 13 (2.9) | 14 (3.2) |
| Roxythromycin | 3 (1.1) | 2 (0.4) | 1 (0.2) |
| Lincomycin | 1 (0.4) | 3 (0.7) | 0 |
| Levofloxacin | 0 | 1 (0.2) | 0 |
| Ceftriaxone | 0 | 2 (0.4) | 2 (0.4) |
