# Supplementary material for: Adherence to smartphone-delivered antimicrobial prescribing guidelines in hospitals in Lao PDR: A stepped-wedge cluster randomized controlled trial
Source: PLoS Med. 2026 Aug 27;23(8):e1005205. doi: 10.1371/journal.pmed.1005205 (PMC13521649; doi:10.1371/journal.pmed.1005205)
Supplement: Supplementary file 12 [file pmed.1005205.s021.docx]

**S11 Table**: Overall antibiotic defined daily dose (DDD) among adult outpatients in six hospitals before and after antimicrobial prescribing guidelines implementation

| Mahosot | 268.57 | 144.75 | 140.67 | 155.13 | 127.6 |
| --- | --- | --- | --- | --- | --- |
| Vientiane Province | 86.4 | 80 | 29.33 | 100.46 | 85 |
| Savannakhet | 62.5 | 54.12 | 16.83 | 27.58 | 18.25 |
| Salavan | 86.28 | 118.43 | 139.14 | 142.45 | 150.83 |
| Luang Namtha | 52.5 | 56.23 | 20.17 | 76.83 | 56.33 |
| Xiengkhuang | 100.24 | 181.69 | 145.85 | 74.42 | 95.29 |
|  | Period 1 | Period 2 | Period 3 | Period 4 | Period 5 |

**Note: white area** = group with no antimicrobial prescribing guidelines, **green area** = group with paper-based version of prescribing guidelines (PP-G), **blue area** = group with application-based version of prescribing guidelines and antimicrobial stewardship training (App-G). **Period 1** = Pre-guideline, **Period 2** = Month-6 after PP-G implementation (all hospitals), **Period 3** = First survey after App-G implementation in Xiengkhuang and Luang Namtha or month-11 after PP-G introduction, **Period 4** = Second survey post-App-G for Xiengkhuang and Luang Namtha, first survey post-App-G for Salavan and Savannakhet, or month-17 after PP-G introduction, **Period 5** = final survey after all clusters had App-G access or month-22 after PP-G introduction.
