# Supplementary material for: Adherence to smartphone-delivered antimicrobial prescribing guidelines in hospitals in Lao PDR: A stepped-wedge cluster randomized controlled trial
Source: PLoS Med. 2026 Aug 27;23(8):e1005205. doi: 10.1371/journal.pmed.1005205 (PMC13521649; doi:10.1371/journal.pmed.1005205)
Supplement: Supplementary file 13 [file pmed.1005205.s023.docx]

**S2 Text:** Stata code and output for sample size calculation and statistical analysis

**Study title:** Evaluating the impact of a Lao language mobile phone antimicrobial use guideline application on antimicrobial prescribing in Laos

**Short title:** LAMPA (Lao Anti-Microbial Prescribing App)

This supplementary material provides the Stata code used for the sample size calculation and the primary statistical analyses

**Sample size calculation**

The sample size was calculated based on a stepped-wedge cluster randomised trial design with one baseline measurement and one measurement prior to each intervention step, resulting in three steps, including the baseline period and outcome measurement after all hospitals exposed to the intervention, i.e., five measurement periods in total. The calculation incorporated assumptions on baseline adherence (30%), expected effect size (15%), intra-cluster correlation (0.05), and cluster size (40 doctors), and was designed to achieve >80% power at a 5% significance level.

The Stata (v18.0) syntax and output for sample size calculation:

**Syntax:**

steppedwedge, binomial power complete(1) k(2) steps(3) p1(0.3) p2(0.45) m(40) rho(0.05) alpha(0.05)

**Primary analysis model**

The primary analysis was conducted using a mixed-effects logistic regression model to assess the association between the intervention and antimicrobial adherence. The binary outcome (*adh*) was modelled with *intervention* as the primary exposure. This analysis was restricted to inpatient data. Random effects were used to account for clustering with hospital-time and also by patient.

melogit *adh* *intervention* *i.age_group* *sex* *i.dep_ipd* *proph_treat* *ib2.seq_0_1_2* if *ipd_opd*==1 || *clustime_intervention*: || *id*: , or

**The model included:**

- Outcome variable (*adh*)
- Binary intervention: 1= App-G; 0= PP-G (*intervention*)
- Age group: ≤1 year (reference); >1-≤5 years; >5-<15 years; ≥ 15 years (*age_group*)
- Sex (*sex*)
- Hospital inpatient department: surgery (reference); obstetrics and gynaecology; medicine; paediatrics; intensive care; other (*dep_ipd*)
- Prescribing indications, whether for prophylaxis or treatment of infection (*proph_treat*)
- Intervention sequence: 2= sequence 1(Xiengkhuang and Luang Namtha); 1= sequence 2 (Salavan and Savannakhet); 0= sequence 2 (Mahosot and Vientiane Province) [reference] (*seq_0_1_2*)
- Random intercept at the cluster-time level (*clustime_intervention*)
- Patients, whether inpatients or outpatients: 1= inpatient; 0= outpatient (*ipd_opd*)
- Random intercept for clustering by patient (*id*)
