# Supplementary material for: Adherence to smartphone-delivered antimicrobial prescribing guidelines in hospitals in Lao PDR: A stepped-wedge cluster randomized controlled trial
Source: PLoS Med. 2026 Aug 27;23(8):e1005205. doi: 10.1371/journal.pmed.1005205 (PMC13521649; doi:10.1371/journal.pmed.1005205)
Supplement: Supplementary file 14 [file pmed.1005205.s024.docx]

# **S4 Text: Statistical Analysis Plan**

**Study title:** Evaluating the impact of a Lao language mobile phone antimicrobial use guideline application on antimicrobial prescribing in Laos

**Short title:** LAMPA (Lao Anti-Microbial Prescribing App)

Version 0·13; Date 4th September 2023

**Principal Investigator:** Vilada Chansamouth

**Co-investigators:** Elizabeth Ashley, Paul Newton, Paul Turner, Rogier van Doorn, Mayfong Mayxay, Nicholas Day, Yoel Lubell, Sue J Lee, Mavuto Mukaka, Manivanh Vongsouvath, Andrew Simpson, Dr Sommai Keomany, Dr Khamla Silavanh, Dr Nunthakone Sihapanya, Dr Phouvieng Duangmala, Dr Khamkeo Mivapadith, Dr Khamsai Detleuxay, Dr Valy Keoluangkhot, Dr Sommana Rattana

**Funder**: Wellcome Trust

**Study sponsor**: University of Oxford

Written by:	Sue Lee:	Date	Signature

Mavuto Mukaka:	Date	Signature

Vilada Chansamouth:	Date	Signature

Reviewed by:	Elizabeth Ashley:	Date	Signature

**Introduction**

Antimicrobial resistance (AMR) is a major global public health concern with rising frequencies of AMR in key pathogens to key antimicrobials. The evidence suggests that this increase is accelerated by use, misuse or overuse of antimicrobials (1). Awareness of AMR is relatively recent in Laos but it is a growing concern. Few data on hospital antimicrobial use exist in Laos (2). High proportions of inpatient antimicrobial use in Laos have been reported (3). The Lao National standard treatment guidelines (STG) were first established in 1998 and the latest version was published in 2012. There are sporadic specific local treatment guidelines with updated versions every few years. The first Lao antimicrobial prescribing guidelines were made available for use in 2021 (4, 5). Antimicrobial prescribing guidelines delivered by mobile phone app are now widespread in many countries. Some evidence suggests that antibiotic guidelines on a mobile phone app increased guideline adherence by prescribers, reduced unnecessary antibiotic use in hospital, reduced poor outcomes, improved quality of care and reduced antimicrobial consumption cost (6-8). There is evidence that multi-activity antimicrobial stewardship (AMS) programs could significantly reduce the proportion of therapeutic and prophylactic antibiotic prescriptions in both in- and outpatients from a study of a large patient population from China (9). This document sets out the statistical analysis plan for the LAMPA study, an open cohort stepped-wedge cluster randomized controlled trial (SW-CRT) to compare the proportion of antimicrobial prescriptions adherent with prescribing guidelines delivered by mobile phone application (app) versus paper-based prescribing guidelines in both in- and outpatients in six general hospitals in Laos.

**Study objectives**

**Primary objective**

To compare the proportion of antimicrobial prescriptions adherent with prescribing guidelines delivered by mobile phone application (app) versus paper-based prescribing guidelines in both in- and outpatients in six general hospitals in Laos

**Secondary objectives**

1. To compare the proportion of patients admitted to hospital receiving an antimicrobial prescription before and after introduction of the paper-based prescribing guidelines; and the mobile phone app prescribing guidelines + AMS training
2. To compare the change in antimicrobial defined daily doses (DDDs) in both in- and out patients after introduction of paper-based versus mobile phone app prescribing guidelines plus antimicrobial stewardship (AMS) training to prescribers
3. To compare the change in proportion of inpatients and outpatients receiving antimicrobials via the intravenous (IV) route between paper-based and mobile phone app prescribing guidelines + AMS training
4. To compare the AMS knowledge level of the prescribers before and after prescribing guidelines delivered by mobile phone app and AMS training
5. To compare the prescriber satisfaction with/acceptability of paper-based prescribing guidelines versus mobile phone app prescribing guidelines

**Analysis principles**

**Study design**

The study design is an open cohort cross-sectional stepped wedge cluster randomized controlled trial (SW-CRT) in one central hospital (Mahosot Hospital (MS – 450 beds) and five provincial hospitals across Laos (Vientiane (VTP – 100 beds), Xiengkhuang (XK – 150 beds), Luang Namtha (LNT – 50 beds), Savannakhet (SVK – 200 beds) and Salavan (SV – 70 beds) Provincial Hospitals) for 16 months.

**Sample size calculation**

A point prevalence survey of hospital antimicrobial use in four general hospitals in Laos in 2017 showed 13% compliance to Lao treatment guidelines (LOMWRU unpublished data) but previous guidelines did not contain much information on antibiotic prescribing, therefore reliable baseline data are lacking. The sample size of 6 hospitals was estimated assuming 30% of antimicrobial prescription adherence with the paper-based prescribing guideline (reference), and assuming a 0.05 intra-cluster correlation coefficient, with an average of 40 doctors at each hospital, and a design effect of 0.446. Using the Stata package “steppedwedge” (10), we estimated that this will give us >80% power to detect a 15% minimum difference after introduction of the mobile phone app prescribing guidelines at a 0.05 significance level.

**Randomization procedure**

This is a 3-step trial with one baseline measurement. Six participating hospitals have been divided into 3 pairs based on the geographical location and convenience for conducting the surveys. The first paired hospitals include two provincial hospitals from the north of Laos (LNT and XK), the second pair include hospitals in the center (MS-central referral hospital and VTP which is 80 km north of the capital). The third pair include two provincial hospitals from the south of Laos (SVK and SV). As there are only 3 orders to be assigned, clusters will be randomly assigned to one of three start dates to have access to the study intervention using simple random sampling. Randomization processes will be performed before the start of the project and the order of each paired hospital to intervention introduction will be recorded. Neither hospitals nor investigators will be blinded to the introduction of the intervention.

**Study intervention**

The intervention is antimicrobial prescribing guidelines delivered via a mobile phone application (MicroGuide) + antimicrobial stewardship training. The control is the paper-based version of the antimicrobial prescribing guidelines.

**Study endpoints**

**Primary endpoint**

The proportion of antimicrobial prescriptions fully adherent with prescribing guidelines delivered by mobile phone app versus paper-based prescribing guidelines at month 16.

**Secondary endpoints**

1. Proportion of patients admitted to hospital receiving an antimicrobial prescription before and after introduction of the paper-based prescribing guidelines; and the mobile phone app prescribing guidelines + AMS training at month 16.
2. Total antimicrobial defined daily doses (DDDs) prescribed in mobile phone app versus paper-based groups at month 16
3. Proportion of patients prescribed antimicrobials by the intravenous route in mobile phone app versus paper-based groups at month 16
4. AMS knowledge level of prescribers prescribed in mobile phone app versus paper-based groups at month 16
5. Prescriber satisfaction/acceptability of the prescribing guidelines in mobile phone app versus paper-based groups of 4 months’ exposure in each group

**Outcome measurement**

Outcomes of this study will be measured by:

1. point prevalence survey (PPS) of hospital antimicrobial use
2. prescriber questionnaires (feedback survey on paper-based and app-based version of the guidelines)
3. AMS knowledge surveys of prescribers

The outcomes will be measured every four months. This will give five-outcome measurements in total (excluding baseline measurement at start), until the trial completes at month 16.

**Data cleaning**

All data will be entered by the PI and trained research staff. Only the PI of the project and project data management team will have access to the raw data. Data checking and cleaning will be done by the PI after each survey. Data inconsistency will be discussed with the research team immediately. Stata version 14 or higher will be used for data cleaning. The original version of the trial dataset will be kept separately. A do file (.do) of data cleaning will also be created.

**Statistical software analysis**

Analyses will be done using Stata 14.0 or higher.

**Definitions of adherence to the guidelines:**

1. Full adherence with the guidelines = using correct antimicrobial agent(s) and correct dose based on the provided antimicrobial prescribing guidelines. Except elective surgical prophylaxis which includes antimicrobial use duration (using correct antimicrobial agent(s), correct dose and correct duration based on the provided antimicrobial prescribing guidelines)
2. Partial adherence with the guidelines = using correct antimicrobial agent(s) with incorrect dose based on provided antimicrobial prescribing guidelines
3. Non-adherence with the guidelines = using a different antimicrobial agent(s) compared to that suggested in provided antimicrobial prescribing guidelines

**Categorical variable:**

a. Age category**:** The actual age will be collected and described in both continuous variable format and categorical variable format. Age will be categorized into different groups as below:
- 0 to <28 days (neonatal)
- 1 month to ≤ 1 year (infant)
- >1 year to ≤ 5 years
- >5 years to ≤ 14 years
- ≥ 15 years
  b. Departments: There are 5 main types of departments in the participating hospitals, including medicine, surgery, paediatrics, obstetrics & gynaecology and intensive care unit (either children or adult or combine). Other wards falling outside these categories such as specialty or psychiatry will be categorized as “other”.
  c. Number of antimicrobials: Number of antimicrobials per patient will be grouped into 3 categories including
- 1 antimicrobial
- 2 antimicrobials
- 3 or more and antimicrobials
  d. Prescribing indication
- Treatment of infection = treatment of any suspected or confirmed infection
- Medical prophylaxis =
  - Medical prophylaxis-general: e.g. gastrointestinal bleeding, liver cancer, prophylaxis for opportunistic infections in HIV patients, malnutrition, neonatal infection prophylaxis (including preterm delivery, low birthweight or birth asphyxia without sign or symptoms of infections)
  - Medical prophylaxis-trauma: e.g., head, eye, chest, abdomen, knee, leg, arm trauma (mostly from road traffic crashes)
  - Medical prophylaxis-vaginal delivery: normal delivery via vaginal route with or without episiotomy
- Surgical prophylaxis = Antimicrobial(s) given for prophylaxis purposes, e.g., elective surgery
- Unclear = Not enough information to indicate why antimicrobials were prescribed (e.g. pleural effusion or diarrhoea (only) without further information of possible cause)
- Unknown = Not known why antimicrobial(s) was given

1. **Reporting baseline characteristics**

Six hospitals participate in this study. They are grouped into 3 clusters (2 hospitals per cluster). There are 5 survey rounds in total for each hospital. Characteristics of variables (from OPD and IPD PPS; and prescriber surveys) from each cluster and phases (control vs intervention) are collected in each survey. They will be presented and compared on tables/charts as appropriate.

Normally distributed continuous baseline characteristics will be summarized with means and standard deviation or 95% confidence intervals. Skewed continuous data will be summarized using median and inter-quartile range (± range). Categorical data will be presented as frequency and percentages. Test of trends, Chi-square or Fisher’s exact tests will be used as appropriate. Variables such as number of years on medical career, specialty, and departments where prescribers are based will be summarized by hospital.

2. **Analysis of primary outcome**

The primary outcome is the proportion of antimicrobial prescriptions adherent with prescribing guidelines delivered by mobile phone app versus paper-based prescribing guidelines at month 16.

A logistic mixed effects regression model (to adjust for clustering) will be used to compare the proportion of antimicrobial prescriptions adherence measured cross-sectionally at month 0, 4, 8, 12 until month 16 after introduction of prescribing guidelines by mobile phone application versus paper-based. The model will compare all steps in the intervention from month 4 to month 16 with all steps in the reference arm from month 0 to month 12. Odds ratios and the corresponding confidence intervals will be obtained and reported. Tests of significance will be performed at 5% level.

Because calendar time is also a potential confounder in stepped-wedge trials, the model will be adjusted for the effect of time. The duration of exposure of each hospital to the reference or intervention arm will be included in the model as interaction term with the study arm to assess whether the duration of exposure is the effect modifier. Initially a model with fixed effect for time, implying a common secular trend across steps and clusters, will be analysed with adherence to guidelines as the outcome. The model will consider the correlation among the outcome’s measurements from the same hospital (cluster) by adjusting for clustering in the model.

Sensitivity analysis will explore seasonal variations and variations in secular trends across clusters and allow treatment effect (i.e., effect of paper-based or mobile phone application on adherence) to vary across clusters and time. For example, a fixed effect interaction between time and each pair of hospitals (strata) for examining varying secular trend across clusters could be added to the model or the model may incorporate a random effects component between time and cluster. Heterogeneity in adherence across clusters may be considered as a fixed effect interaction between the intervention and strata or, by incorporating a random effect term for the intervention and for each pair of hospitals (strata). Sensitivity analysis will also consider whether adherence varied over time using a fixed effect term for the interaction between intervention and time. This model will only be estimable at time periods that have both intervention and control phases (weeks 4 to 16).

An *a priori* set of variables will be considered for inclusion in a multivariable model (see section Subgroup analysis). Interactions will be considered, as appropriate. The results will be reported in accordance with the Consolidated Standards of Reporting Trials (CONSORT) extension for the stepped wedge cluster randomized trial (SWCRT).

3. **Analysis of secondary outcomes**

Secondary outcome 1: Proportion of patients admitted to hospital receiving an antimicrobial prescription before and after introduction of the paper-based prescribing guidelines; and the mobile phone app prescribing guidelines + AMS training at month 16. Number of patients admitted to hospitals at the time of survey will be a denominator. The overall proportion of antimicrobial prescribing from 2017 to 2020 (before prescribing guidelines) will be compared with the overall proportion of antimicrobial prescribing after introduction to the prescribing guidelines (control phase) and then the overall proportion and antimicrobial prescribing after introduction to the mobile phone-app version of the guidelines and AMS training (intervention phase). The analysis process of this secondary outcome will also follow the same strategy as secondary outcome analysis number 2.

Secondary outcome 2: Total antimicrobial defined daily doses (DDDs) prescribed in mobile phone app versus paper-based groups at month 16: DDD will be calculated using data (antibiotic agent, start date, dose and DDD of each antibiotic from [www.whocc.no](http://www.whocc.no) ) from PPS in both IPD and OPD.

Secondary outcome 3: Proportion of patients prescribed antimicrobials by the intravenous (IV) route in mobile phone app versus paper-based groups at month 16: A logistic mixed effects regression model will be used to compare the overall proportion of the intravenous route: the proportion of the IV route antimicrobial prescriptions will be measured cross-sectionally at month 0, 4, 8, 12 until month 16 after introduction of prescribing guidelines by mobile phone application versus paper-based. The model will compare all steps in the intervention from month 4 to month 16 with all wedges in the reference arm from month 0 to month 12. The duration of exposure of each hospital to the reference or intervention arm will be included in the model as interaction term with the study arm to assess whether the duration of exposure is the effect modifier. The model will consider the correlation among the outcome’s measurements from the same hospital (cluster) by adjusting for clustering in the model. Odds ratios and the corresponding confidence intervals will be obtained and reported. Tests of significance will be performed at 5% level.

As a single time point survey, we will not be able to capture the duration of antimicrobial use in our participated hospitals, except antimicrobial use for elective surgical prophylaxis (single dose or one day prophylaxis). Therefore, we cannot estimate the proportion of switching from intravenous antimicrobial to oral antimicrobial prescribing in a mobile phone group versus paper-based group in this study.

Secondary outcome 4: AMS knowledge level of prescribers in mobile phone app versus paper-based group at month 16: AMS knowledge level of prescribers will be assessed using questionnaires based on information in the prescribing guidelines. There are 18 questions in total. As each prescriber will participate in the antimicrobial stewardship knowledge survey 3 times (first time at baseline, second time at the beginning of the intervention phase and last time at the end of the study (month 16)), before the survey, eight randomly selected questions will be selected to assess the knowledge of each participating prescribers in each survey round. The questionnaires include both single and multiple answer questions. The overall proportion of correct answers in each time point will be compared using a paired test (baseline, after paper-based guidelines (before AMS training and introduction of mobile phone app guidelines) and after AMS training and introduction of mobile phone app guidelines). The scores are coded as 0/1, where 1 =correct and 0= incorrect. Then the knowledge level will be assessed using sum of a total score. Other factors such as extra AMR training program from hospital/MoH/other organizations, level of education and specialties of prescribers will also be considered using index test scores, adjusted for baseline (or previous) score in a Poisson or linear regression model, depending on distribution

Secondary outcome 5: Prescriber satisfaction/acceptability of the prescribing guidelines in mobile phone app versus paper-based groups of 4 months’ exposure in each group: frequency of the use of paper-based and app-based version of the guidelines and frequency of the use of other antimicrobial prescribing guidelines will be descriptively shown at month 0, 4, 8, 12 until month 16. The frequency of the use of the app-based guidelines can also be collected from MicroGuide app directly, as well as which topics the prescribers commonly visit.

Likert scale analysis will be used to evaluate prescriber satisfaction with/acceptability of paper-based compared to mobile phone app delivered antimicrobial prescribing guidelines. The satisfaction/acceptability scale will be assigned in six categories, including: strongly agree=5, agree=4, neutral=3, disagree=2, strongly disagree=1, no opinion/not use=0.

Mann-Whitney U test will be used to assess the differences between paper-based population and mobile phone app population. The satisfaction/acceptability scale will also be stratified by prescriber specialty, departments where they are based on and years of medical career.

**Plans for sub-group analysis**

Sub-group analysis will be considered to investigate the relationship between the outcomes of interest or exposure of interests and other collected variables for some specific questions such as the relationship between departments (IPD/OPD) and how doctors prescribed; or the relationship between number of antimicrobial prescribed and prescribing indications; or spectrum of antibiotics and prescribing indications; or topic most interest in the guidelines and guidelines compliance of that topics. Each variables listed below is a suspected confounder; our exploratory analysis may determine that some of these are in fact effect modifiers. If this is the case, interactions will be explored in models and these variables will be considered for sub-group analysis.

- Survey rounds
- Departments (IPD (paediatrics, obstetrics and gynaecology, medicine, surgery, ICU and other) and OPD)
- Number of antimicrobial prescribed (one or two or more than 2 antimicrobials)
- Route of administration (oral or IV)
- Prescribing indication (treatment of infection, prophylaxis or unknown/unclear)
- Spectrum of antibiotics (narrow and broad)
- AwaRe antibiotic classification
- Indication documented in patient records (e.g. pneumonia, sepsis, skin and soft tissue infection)
- Years of medical work of prescribers
- Specialty of prescribers
- Frequency of accessing paper-based version of the guidelines and mobile phone-app version of the guidelines (data will be collected from MicroGuide app)
- Topics of most interest of the guidelines (data will be collected from MicroGuide app)

**Discussion and conclusion**

The LAMPA trial could have a significant impact in Laos and in other LMICs, and inform policy globally, by improving prescribing and hence reducing AMR. This open cohort stepped-wedge cluster randomized controlled trial is appropriate in the Lao context because it requires fewer sites than a parallel cluster trial but has similar observational periods for reference and intervention phases. As the interventions are delivered by one team travelling to each cluster in turn, implementation in stages is more practical. In addition, all participating hospitals will have access to the novel interventions at the end of the study.

This is the first open cohort stepped wedge cluster randomized controlled trial on antimicrobial prescribing in Laos. This statistical analysis plan outlines the process to measure primary and secondary outcomes of interests, and we also consider sub-group analyses. Like many other studies, we faced challenges during the covid-19 outbreak in Laos which delayed some of our survey rounds but otherwise, the trial was managed as planned. As many doctors were focused on covid-19, this may have affected the number of prescribers participating the AMS training and the number of patients. There were also some AMR activities (e.g., strengthening microbiology laboratories and diagnostic stewardship under Fleming fund supports, AMS activities within a hospital) in some study sites separate from our trial. All of these factors will be considered during the interpretation of our study results. In this statistical analysis plan, we outline details of the planned analysis in advance of LAMPA trial completion. Prior specification of the statistical methods and outcomes facilitate unbiased analyses of these important data.

# **References**

1.	Holmes AH, Moore LS, Sundsfjord A, et al. Understanding the mechanisms and drivers of antimicrobial resistance. *Lancet* 2016;387(10014):176-87.

2.	Chansamouth V, Mayxay M, Dance D, et al. Antimicrobial use and resistance data in human and animal sectors in the Lao PDR: evidence to inform policy. *BMJ Glob Health* 2021;6(12):e007009.

3.	Chansamouth V, Chommanam D, Roberts T, et al. Evaluation of trends in hospital antimicrobial use in the Lao PDR using repeated point-prevalence surveys-evidence to improve treatment guideline use. *Lancet Reg Health West Pac* 2022;9:100531.

4.	Department of Healthcare and Rehabilitation. Lao adult antimicrobial prescribing guidelines. 1 ed: Department of Healthcare and Rehabilitation (DHR); 2020. 157 p.

5.	Department of Healthcare and Rehabilitation. Lao paediatric antimicrobial prescribing guidelines. 1 ed: Department of Healthcare and Rehabilitation (DHR); 2020. 155 p.

6.	Tuon F, Gasparetto J, Wollmann L, Moraes T. Mobile health application to assist doctors in antibiotic prescription - an approach for antibiotic stewardship. *Braz J Infect Dis* 2017;21(6):660-4.

7.	Yoon C, Ritchie S, Duffy E, et al. Impact of a smartphone app on prescriber adherence to antibiotic guidelines in adult patients with community acquired pneumonia or urinary tract infections. *PLoS One* 2019;14(1):e0211157.

8.	Antonello V, Cunegatto M, Rosin E, et al. The Use of Mobile Educational Tools to Improve Antimicrobial Prescription for the Treatment of Acute Pyelonephritis in Pregnancy: A Retrospective Cross-sectional Study. *Rev Bras Ginecol Obstet* 2019;41(2):97-101.

9.	Wang H, Yu X, Zhou H, et al. Impact of antimicrobial stewardship managed by clinical pharmacists on antibiotic use and drug resistance in a Chinese hospital, 2010-2016: a retrospective observational study. *BMJ Open* 2019;8(9):e026072.

10.	Hemming K, Girling A. A menu-driven facility for power and detectable-difference calculations in stepped-wedge cluster-randomised trials. *Stata J* 2014;14(2):363-80.
