# Supplementary material for: Adherence to smartphone-delivered antimicrobial prescribing guidelines in hospitals in Lao PDR: A stepped-wedge cluster randomized controlled trial
Source: PLoS Med. 2026 Aug 27;23(8):e1005205. doi: 10.1371/journal.pmed.1005205 (PMC13521649; doi:10.1371/journal.pmed.1005205)
Supplement: Supplementary file 17 [file pmed.1005205.s029.docx]

**Response to editors and reviewers**

Dear Editors and Reviewers,

We would like to thank you for your time and effort in reviewing this manuscript. Please find our point-by-point responses to your questions and comments below.

**Comments from the academic editor:**
This study addresses a very relevant global issue of antimicrobial resistance. The authors should be commended in conducting a trial to optimize antimicrobial prescribing in a low-middle-income country where antimicrobial resistance is a major issue.
Despite being a negative study, I think the results are very insightful and highlight the knowledge to practice gap. Although there were available guidelines published, which were provided to the physicians in easily accessible formats, the overall adherence remained quite low. The use of technology (i.e. smartphone app) did not significantly improve adherence, which suggest that improving accessibility to guidelines likely is not enough to improve adherence to guidelines. Therefore, policy makers should consider a multimodal implementation approach. I think the results can still be quite useful to policy makers as a caution that improving access to guidelines through technology likely does not equate to high uptake, usage or adherence to the guidelines.

There are many limitations to the study. In my opinion, the two main limitations are:

1) sample size calculation that is not reproducible and inclusion of only 6 sites that made the study quite underpowered such that there could not be any definitive conclusion.

**Response**: Thank you for your comment. We have amended the sample calculation section to improve clarity (line (lines **230-236**):

“A point prevalence survey of hospital antimicrobial use in four general hospitals in Laos in 2017 showed 13% adherence to national treatment guidelines. However, as previous guidelines contained limited information on antibiotic prescribing, reliable baseline data were lacking. The sample size of six hospitals was calculated assuming 30% of antimicrobial prescription adherence with the paper-based prescribing guideline (reference), an intra-cluster correlation coefficient of 0.05, and an average of 40 doctors per hospital. Using the Stata “steppedwedge” package [22] (S1 Text), it was estimated that this would provide more than 80% power to detect a 15% difference after introduction of the smartphone app prescribing guidelines at a 0.05 significance level.”

We have checked the sample size calculation and confirmed that it is reproducible. Please find the STATA code and output below.

steppedwedge, binomial power complete(1) k(2) steps(3) p1(0.3) p2(0.45) m(40) rho(0.05) alpha(0.05)

We have also provided additional details on the sample size calculation STATA code in S1 Text (also available at <https://doi.org/10.6084/m9.figshare.32051097> ) to ensure that readers can reproduce it.

We have also amended the limitations section to reflect the sample size (lines **516–520**):

“Second, although the study design was adequately powered based on available data at the time, in the end, the inclusion of only six participating hospitals may have limited statistical power for subgroup analyses of guideline adherence in specific infectious diseases or conditions, given the relatively small number of cases. In addition, variation in cluster sizes meant that some clusters contributed fewer observations, which may have reduced the ability to detect true differences."

2) Use of point prevalence survey of antimicrobial use, which is such a small short time window of data collection compared to the entire period that may not be representative of the entire period - authors should acknowledge this as a limitation in the discussion

**Response**: We agreed that Point Prevalence Survey (PPS) cannot capture the full extent of hospital AMU throughout the study period. However, this method is more practical in low resource settings such as Laos, where the medical record systems and human resources are limited. Repeated data collection can provide useful insights into antimicrobial prescribing patterns at each time point, which aligns with the stepped wedge design. I have acknowledged this limitation at the end of the limitation section, please find lines **520-523**:

“Third, the PPS method was used to measure outcomes at each time point. While it provides a useful snapshot of hospital antimicrobial use, it does not capture the full picture of antimicrobial use in the study hospitals over the entire study period”

The authors will absolutely need to address the comments from the rigorous statistical review adequately.

**Response**: Thank you, we have responded to the reviewers’ comments as below.

In addition to the reviewer comments, I have 2 major comments for authors:
- There is nowhere in the manuscript or references available of what the national guidelines actually recommend. The authors should provide the guidelines in the appendix or at a minimum a summary of the antibiotic recommendations so that at least the readers can get a sense of what these recommendations are and how valid they are.

**Response:** We have amended reference 21 by added the link to the guideline so that the readers can access to the guidelines, please note that the guidelines are in Lao language, with minimal translation. Please find lines **616-620**:

“21. Department of Healthcare and Rehabilitation (DHR). Lao Antimicrobial Prescribing Guidelines: Eolas Medical; 2021 [Accessed: 01/04/2026]; Available via Eolas Medical Eolas Medical 13.1.1: <https://app.eolasmedical.com/auto-login/EOLAS%23ORG%23staging-department-of-health-care-and-rehabilitation-ministry-of-health-of-lao-pdr%232275a611-795a-403a-ae03-48a895cdcd4f%23ab70cbb0-6d8c-4af3-a712-a8e45841b3e5>].”

- The authors had acknowledged that most studies defined appropriateness based on antibiotic choice alone. This study used a much more stringent criteria of also considering dose (and duration for prophylaxis). Clinically, there are many valid reasons to use a different dose based on patient factors (e.g. renal function, weight) and the fact that many antibiotics recommended dose is actually a range. I think the authors should consider doing a sensitivity analysis of judging appropriateness without the dose criteria.

**Response**: we have added a section of guidelines adherence based on antimicrobial agent only. Please find lines **337-340**:

“When adherence was assessed based on antimicrobial agent selection alone, overall adherence in the App-G group was 34.6% (385/1,112; 95% CI: 31.8-37.5) and 30.6% (416/1,360; 95% CI: 28.1-33.1) in the PP-G group. There was no evidence of a difference between groups, with an adjusted odds ratio (aOR) of 1.02 (95% CI: 0.7-1.4; *p=* 0.915) for App-G compared with PP-G”

And lines **390-392** for outpatients:

“Using antimicrobial agent selection as the criterion for adherence, 43.5% of prescriptions in the App-G group were adherent (656/1,507; 95% CI: 43.0-46.1), compared with 42.2% in the PP-G group (511/1,212; 95% CI: 39.4-45.0). The aOR for guideline adherence was 0.82 (95% CI: 0.7-1.0; *p=* 0.062) for App-G relative to PP-G.”

**Comments from the reviewers:**

**Reviewer #1:**

Article: Assessment of adherence to antimicrobial prescribing guidelines delivered by a smartphone application in hospitals in Lao PDR: Stepped-wedge cluster randomised controlled trial
**Strengths:**
1. Relevance: The study addresses a critical issue in global health—antimicrobial resistance (AMR)—and proposes an innovative solution for a low- and middle-income country (LMIC), Laos. It explores the potential of mobile health technologies in improving guideline adherence in healthcare systems with limited resources.
2. Study Design: The stepped-wedge cluster randomized controlled trial (SW-CRT) design is robust, allowing for a clear comparison between the app-based intervention and the paper-based guidelines, with randomization across six hospitals, enhancing the generalizability of the findings.
3. Data Analysis: The use of mixed-effects regression models to account for time and clustering effects provides a sophisticated approach to analyzing the data. This method is appropriate for handling the complexities of clustered data in a longitudinal trial.
4. Contribution to AMR Control: The study offers important insights into antimicrobial stewardship in Laos, a country where AMR is becoming an increasing concern. It provides a foundation for future interventions and highlights the challenges of improving adherence to guidelines even with technological interventions.
5. Multi-level Outcomes: The study considers multiple relevant outcomes, including prescribing patterns, adherence to guidelines, and the impact on antimicrobial consumption, which are crucial for assessing the effectiveness of the intervention in real-world settings.

**Response:** Thank you very much for your feedback, it is greatly appreciated.

**Weaknesses**:
1. Limited Evidence of Impact: Despite the innovative approach, the study finds insufficient evidence that the smartphone app improved adherence to guidelines compared to paper-based methods. This suggests that simply providing a mobile app is not enough to change prescribing behavior, which may limit the practical implications of the findings.

2. Low Adherence Rates: The overall adherence rates, even with the intervention, are quite low (17% with paper-based and 25.6% with the app). This suggests that more complex, multifaceted interventions beyond just guideline delivery are needed to address the challenges of AMR.

**Response to weakness 1 and 2:** we have acknowledged these limitations and addressed them in our discussion and conclusion sections, please find lines **530-535**:

“These findings suggest that guidelines alone are insufficient to ensure appropriate antimicrobial use. The urgent priority is to tackle the broader, multifaceted barriers, including improving guidelines awareness, ensuring access to appropriate technology and skills, and strengthening healthcare services in the context of human resource constraints. Regular feedback and sustained support from senior hospital leadership and the Ministry of Health is also essential to promote consistent guideline use and improve patient outcomes.”

3. Potential Confounding Variables: While the study adjusts for some confounders, the lack of deeper exploration into other factors influencing adherence (e.g., prescriber behavior, institutional support, availability of resources, COVID scenario, percentage of prescribers familiar with using mobile tools) may have affected the outcomes.

**Response:** We did not track individual prescribers to assess changes in prescribing behaviour over time. Prescribing data were collected directly from hospital records without information on the identity of the prescriber for each patient. This limitation has been acknowledged and discussed in the limitations section (lines **523-525**):

“Fourth, we did not measure individual prescriber behaviour changes after the intervention implementation. Data were collected from patients' documents, from which it was difficult to ascertain which prescribers were responsible for each prescription.”

In addition, we also conducted in-depth interviews with 16 prescribers from two participating hospitals to better understand their prescribing behaviours and other challenges. Digital literacy was discussed in our study (lines **493-497**):

“In some regions of Laos, healthcare providers may not have full digital literacy. Even among those who do, there may be a reluctance to adopt new technology due to unfamiliarity or complexity. In our study, all participating prescribers had access to smartphones, and the application was designed to function offline; therefore, internet connectivity limitations were unlikely to be a major barrier to its use in this setting. However, differences in confidence and experience with mobile applications may still have influenced uptake.”

However, these factors were not included in our model because they were collected differently.

4. Generalizability: The study's findings are based on a small number of hospitals in Laos, which may limit the generalizability of the results to other LMICs or even within different regions of Laos. More extensive studies across diverse settings could provide a clearer picture of the app's potential.

**Response**: While we only studied six hospitals these represent around one third of provincial hospitals in the country. Our experience suggests challenges are similar across Laos, although with minor differences in the epidemiology of certain infectious diseases such as melioidosis. The six participating hospitals were selected based on practical considerations, including time, study team capacity, and budget constraints. Importantly, these hospitals were purposively chosen to reflect geographical diversity across Laos, with two hospitals in the north, two in the central region, and two in the south. We believe this distribution provides reasonable representation of 17 provinces and a capital across the country.

5. Implementation Challenges: The study does not extensively discuss the barriers to effective implementation of the smartphone app, such as technological literacy, smartphone availability, or infrastructure limitations, which are critical in LMICs like Laos.

**Response**: Digital literacy and familiarity with mobile applications may have influenced uptake and effective use. We have clarified this point in the discussion (lines **493-499**):

“In some regions of Laos, healthcare providers may not have full digital literacy. Even among those who do, there may be a reluctance to adopt new technology due to unfamiliarity or complexity. In our study, all participating prescribers had access to smartphones, and the application was designed to function offline; therefore, internet connectivity limitations were unlikely to be a major barrier to its use in this setting. However, differences in confidence and experience with mobile applications may still have influenced uptake. This digital divide can result in lower utilization of app-based guidelines; therefore, some prescribers might have continued using the traditional paper-based version rather than switching to app-based version.”

6. I missed having more clear and objective information about the app itself, its features, the layout, and the number of people who downloaded it, as well as the app´s promotion to the prescribers.

**Response**: The app-based guidelines is provided in the Intervention section (lines **182-187**):

“The guidelines were in Lao language, separated into paediatric and adult editions. There were 90 infectious diseases topics for treatment of infections and 12 topics addressing surgical and medical prophylaxis in the adult guidelines. The paediatric guideline included 66 infectious diseases topics for treatment of infections and 13 topics for surgical and medical prophylaxis. Both paediatric and adult guidelines included an antibiotic stewardship section. The guidelines were endorsed by the Lao Ministry of Health. The MicroGuide platform (since rebranded as Eolas Medical app [21]) was used to deliver the app-based guidelines”

We have also provided access to the guidelines and layout via reference 21:

“21. Department of Healthcare and Rehabilitation (DHR). Lao Antimicrobial Prescribing Guidelines: Eolas Medical; 2021 [Accessed: 01/04/2026]; Available via Eolas Medical Eolas Medical 13.1.1: <https://app.eolasmedical.com/auto-login/EOLAS%23ORG%23staging-department-of-health-care-and-rehabilitation-ministry-of-health-of-lao-pdr%232275a611-795a-403a-ae03-48a895cdcd4f%23ab70cbb0-6d8c-4af3-a712-a8e45841b3e5>].”

**Reviewer #2:**

This manuscript presents a stepped wedge trial examining the implementation of antimicrobial prescribing guidelines via paper and app in Laos. The study did not find evidence of improved adherence to guidelines in either delivery format. The study also provides an important baseline of antimicrobial use outside of high-income settings.

The manuscript is well written and does an excellent job of contextualising the research and establishing its importance. Unfortunately, I found the methodological and statistical detail insufficiently comprehensive and clear. I also suggest the Results section could do with editing to improve readability.

Detailed feedback on the manuscript is provided below. My primary focus has been methodological, but I have made a few broader points as well. All items are major unless indicated otherwise.

1. I'm afraid I don't understand the authors' use of the term "point prevalence survey" throughout the manuscript. Does this mean all prescribing data over the course of the trial was not used? The authors say point prevalence surveys were conducted four-monthly but the periods in Figure 1 are one, two, or three months, so I don't yet understand how the surveys and the step wedge stages interrelate. It is crucial to communicate these characteristics of the trial as clearly and simply as possible, particularly for a more complex design like a stepped-wedge CRT.

**Response**: Point Prevalence Surveys (PPS) were used to collect outcome (antimicrobial guideline adherence) data in this study as they are practical in low-resource settings such as Laos, where electronic prescribing is typically unavailable, and logistical capacity is limited. This approach has become the standard way to conduct AMU surveillance in LMICs and an example of the methodology is the Global-PPS (https://www.global-pps.com/). A standard PPS approach was applied across all study sites at each time point to ensure comparability. In stepped-wedge design, multiple data collection rounds are required because the intervention is introduced sequentially across clusters. Collecting data at each time point allows estimation of the intervention effect as clusters transition from control to intervention.

In Fig 1 and S2 Fig, period one (Jan-Mar 2021), two (Jun-Jul 2021), three (Nov-Dec 2021), four (Apr 2022) and five (Sep-Oct 2022) represent the study time point. Period 1 corresponds to the pre-guideline (baseline). Period 2 was month 6 after implementation of the paper-based guidelines (PP-G). Period 3 was first survey after implementation of the app-based guidelines (App-G) in Xiengkhuang and Luang Namtha, and month 11 after PP-G implementation. Period 4 was the second post-App-G survey in Xiengkhuang and Luang Namtha, and the first post-App-G survey in Salavan and Savannakhet, corresponding to month 17 after PP-G introduction. Period 5 was the final survey after all clusters had access to App-G, and month 22 after PP-G introduction. We have also amended Figure 1 to improve clarity.

2. I suggest the authors provide a small amount of detail on what a stepped wedge trial is, how it differs from a traditional cluster randomised trial, and why its use is appropriate for this research. I also suggest including a reference for more details on this type of study design for readers who are unfamiliar (e.g., https://www.bmj.com/content/350/bmj.h391 or similar).

**Response**: Thank you for your feedback, the rationale for choosing a stepped-wedge design for this study is clarified in the “Study design and participants” section (lines **148-154**):

“The SW-CRT design, where an intervention is introduced sequentially at different sites, was chosen as the most suitable for the context, as it required fewer sites while allowing for similar observational periods during both control and intervention phases. Given that the interventions were delivered by a single team traveling to each cluster in sequence, logistical constraints such as limited personnel, time, and travel requirements, also influenced the implementation schedule [20], therefore, a staged implementation approach was more practical than a parallel trial. ”

I have also added a reference for the readers to explore further details:

“20. Hemming K, Haines TP, Chilton PJ, Girling AJ, Lilford RJ. The stepped wedge cluster randomised trial: rationale, design, analysis, and reporting. BMJ. 2015;350:h391.”

3. I'd suggest against using the word "pair" in the "Randomisation and masking" section (lines 164-170). Generally, when "pair" is used in the context of randomisation, it means paired samples and this left me very confused. Suggest using "groups of two" or similar instead.

**Response**: Thank you, we have amended this as suggested throughout the manuscript

4. Also in this section, I found the phrase "Hospital pairs were assigned to interventions start dates using simple random sampling…" confusing as it suggests the actual date of implementation was chosen at random. I think the authors mean the implementation sequence of the hospital groups was randomised?

**Response**: We have clarified the randomisation and masking section (lines **171-175**):

“Simple random sampling (lottery method) was used to determine the sequence in which the three pre-defined groups would receive the intervention (i.e., first, second, and third rollout steps). Randomisation was applied only to the sequence of implementation across groups, rather than the actual calendar start dates. The allocation of the implementation sequence was carried out by an individual not involved in study implementation. Neither hospitals nor investigators were masked to the introduction of the intervention.”

5. Some wording in the Outcomes section seemed to contradict itself. The authors say "Full adherence with the guidelines was defined as the prescriptions with correct antimicrobial agent(s) and correct dose." but then go on to say "Non-adherence to the guidelines was defined as using a different antimicrobial agent(s) compared to that suggested in the provided antimicrobial prescribing guidelines." This means it's unclear if using the correct agent but the incorrect dose is adherent or not. Suggest the authors revise this paragraph to ensure the rules governing whether a prescription was within guidelines or not are abundantly clear.

**Response**: We have clarified the outcomes section, please find lines **209-211**:

“Non-adherence to the guidelines was defined as any deviation from the guidelines, including use of an incorrect antimicrobial agent(s), or use of the correct agent(s) with an incorrect dose, and/or incorrect duration for elective surgical prophylaxis.”

6. I am also a bit confused about the descriptions in the Procedures section. The authors say switching happened approximately every four months resulting in a 1:1 ratio of intervention to reference period of observation but this does not seem to be what happened. Perhaps softening the language by saying "The original plan…" or "The intention was…", then the authors can explain what actually happened (COVID delays etc.) in the Results without this conflicting with the Methods.

**Response**: Thank you for this comment. The methods section describes our planned study design, in which intervention steps were scheduled at approximately four-month intervals. However, due to COVID-19-related logistical constraints, the actual intervals between implementation steps were extended to approximately 5 to 6 months. This deviation from the original plan has been clarified in the Results section (lines **261-265**):

“The trial operated over 22 months, from January 2021 to October 2022. The first group of hospitals (XK and LNT) received the intervention in June-July 2021 (sequence-1), the second group (SV and SVK) in November-December 2021 (sequence-2), and the last group (VTP and MS) in April 2022 (sequence-3), with five-to-six-month intervals between each. This was longer than expected due to COVID-19 pandemic restrictions (Fig 1 for hospitalised patients and S2 Fig for outpatients).”

We have also amended the procedure section to improve clarity (lines **222-225**):

“At month-4, the intervention was introduced to the first hospital group. Subsequently, at approximately four-month intervals, each group of hospitals crossed over from the reference phase to the intervention phase until all groups had implemented the intervention. This resulted in an overall 1:1 ratio of intervention to reference observation periods by the end of the trial (month 16)”

7. I was not able to replicate the sample size calculations based on the information provided using the Stata steppedwedge tool referenced. Please provide sufficient detail in a form that maps directly to the inputs required for the Stata steppedwedge tool to allow confirmation of the sample size calculation. The authors could also consider including the relevant Stata code and output as a supplement.

**Response**: Apologies for this oversight. We have now clarified the text in the manuscript (lines **232-236**):

“The sample size of six hospitals was calculated assuming 30% of antimicrobial prescription adherence with the paper-based prescribing guideline (reference), an intra-cluster correlation coefficient of 0.05, and an average of 40 doctors per hospital. Using the Stata “steppedwedge” package [22] (S1 Text), it was estimated that this would provide more than 80% power to detect a 15% difference after introduction of the smartphone app prescribing guidelines at a 0.05 significance level.“

We also provide the command used (with output) and specify the design-pattern matrix used in the supporting information (S1 Text, also available at <https://doi.org/10.6084/m9.figshare.32051097>):

Here is the Stata syntax:

steppedwedge, binomial power complete(1) k(2) steps(3) p1(0.3) p2(0.45) m(40) rho(0.05) alpha(0.05)

8. The descriptions of the statistical modelling approaches in the Statistical Analysis section of the Methods are a good start, but more detail is needed. If modelling results are presented, then enough detail should be included in the Methods so an interested reader can replicate the analyses. Specifically, while the authors have noted the model type they have used ("mixed-effect logistic regression model"), it's important to clearly state the variables in the models (fixed and random effects for hierarchical models, cluster variables for GEE etc.). Of particular importance is how hierarchies are handled e.g., prescription within patient within hospital, as this can cause biased estimates if not modelled correctly. Including abridged modelling code as a supplement can also help when more complex analyses are being undertaken.

**Response**: Thank you for this important comment. We have re-examined the model to account for the hierarchical structure of the data, particularly at the patient level. We therefore refitted the mixed-effects logistic regression model including patient as an additional random effect, alongside hospital and time, to account for clustering. The results from this updated model were very similar to those from the original model and did not change the overall conclusions; specifically, there remained no evidence of a difference in guideline adherence between the App-G and PP-G groups. Tables 2 and 3 have been updated to reflect the final model specification. We have also amended the statistical analysis section to improve clarity (lines **240-252**):

“A mixed-effects logistic regression model was used to account for the hierarchical structure of data and repeated measurements over times within clusters. We followed methodological guidance for stepped wedge trials with binary outcomes [24, 25]. The model compared the proportion of antimicrobial prescription adherence measured in all steps in the App-G from month 4 to month 16 with all steps in the PP-G from month-0 to month-12. The binary outcome (adherence) was modelled with intervention group as the primary exposure. The model included fixed effects for age group, gender (male or female), hospital department (surgery, intensive care, paediatrics, obstetrics and gynaecology, medicine, and other), prescribing indication (prophylaxis or treatment), and sequence (1, 2 or 3). The unit of analysis was indication and random effects for patient and hospital-time were included.

Given the small number of clusters, which may affect the reliability of standard errors in mixed-effects models, results were interpreted with caution, with emphasis on effect estimates and confidence intervals rather than statistical significance alone. Odds ratios (OR) and corresponding 95% confidence interval (CI) are reported. Tests of significance were performed at the 5% level.”

To further support reproducibility, we have added the STATA Syntax for the primary analysis as supplementary material, please see S1 Text (also available at <https://doi.org/10.6084/m9.figshare.32051097> ).

9. Related to the prior point, given only six hospitals were included, I suggest the authors consider the risk of unreliable standard errors and p-values arising from the combination of GLMMs and a small number of clusters (https://www.tandfonline.com/doi/full/10.1080/00273171.2016.1236237). Specific guidance for stepped wedge trials examining binary endpoints is available (https://link.springer.com/article/10.1186/s13063-017-1862-2), and I suggest the authors clearly communicate that they considered this risk and their decision-making around the analytical approach chosen (e.g., choice of test/CI method).

**Response**:

We acknowledge that the use of mixed-effects models with a small number of clusters (six hospitals in this study) may lead to underestimated standard errors and confidence intervals. This limitation was recognised in the context of the stepped-wedge design and binary outcome. A mixed-effects logistic regression model was chosen to appropriately account for the hierarchical structure of the data and repeated measurements over time within clusters. We followed methodological guidance for stepped-wedge trials with binary outcomes (Hayes and Mouton, 2009 [<https://doi.org/10.1201/9781584888178>] and Hemming et al 2017 [ doi: 10.1186/s13063-017-1833-7 ]). Given the limited number of clusters, we interpreted the findings with caution, placing greater emphasis on the magnitude and direction of effect estimates and their confidence intervals rather than statistical significance alone. We have added clarification to the statistical analysis section (lines **249-251**):

“Given the small number of clusters, which may affect the reliability of standard errors in mixed-effects models, results were interpreted with caution, with emphasis on effect estimates and confidence intervals rather than statistical significance alone.”

In addition, the limitations in the discussion text have been modified to (lines **516-519**):

“… although the study was adequately powered based on available data at the time, in the end, the inclusion of only six participating hospitals may have limited statistical power for subgroup analyses of guideline adherence in some specific infectious diseases or conditions, giving the relatively small number of cases.”

10. I also found the use of the word "considered" in this section confusing. I suggest the authors clearly state the models that were actually run (and exclude those that were only considered), ensuring this matches the presented results.

**Response**: We have amended the statistical analysis section as suggested throughout.

11. What is meant by "WHO AWaRe Access group antibiotics"? This is mentioned for the first time in the Results with no background in the Methods. Please ensure that all presented results are based on already introduced methods. Please also provide at least one reference to provide more information on external resources.

**Response**: The WHO AWaRe classification is a tool used to monitor antibiotic consumption. Antibiotics are classified into three groups: Access, Watch, and Reserve. We have added the full name (lines **279-280**) and a reference 26 for readers to obtain further information:

26. WHO. AWaRe classification of antibiotics for evaluation and monitoring of use, 2023: WHO; 2023 [Accessed: 14/04/2026]; Available from: <https://www.who.int/publications/i/item/WHO-MHP-HPS-EML-2023.04>.

12. [MINOR] I think it's worth defining "empirical" vs. "targeted" in the Methods because otherwise a reader has to go to a table note to understand the text in the Results.

**Response**: We have added further clarification after mention it (lines **309-311**):

“(empiric or targeted [prescribing based on confirmed microbiological results [e.g., conventional culture or Gene-Xpert for tuberculosis]),”

13. I'm not clear why the authors present results "… after 4-6 months of exposure…" (line 282) and what exactly is meant by this. Is this when the point prevalence survey was done? I think the data acquisition processes must be explained much more clearly.

**Response**: Thank you for this comment, we have amended the text to improve clarity (lines **296-299**):

“After all hospitals had access to the paper-based guidelines for approximately 4-6 months, surveys were conducted across all hospitals to assess adherence. At this time point, adherence to the prescribing guidelines was 17.0% (127/746; 95% CI: 14.9-19.9). By the end of the trial, when all hospitals had access to both guideline formats, overall, adherence increased to 28.0% (202/722; 95% CI: 24.7-31.4); *p*<0.001 (Fig 1 and Fig 2).”

14. [MINOR] I found the volume of results presented in the Results section to be somewhat excessive. I'd suggest the authors edit down the results presented in the text to the most salient and allowing an interested reader to examine the less salient statistics in the supplementary materials. I appreciate this is subjective, which is why I have said this point is "minor", but I found it negatively affected readability.

**Response**: Thank you for your feedback. We have revised the Results section and reduced the density of Figure 1 by separating it into Figure 1 and Supplementary Figure S2 (S2 Fig). We have also removed some parts of the Results section. For example, we removed “, while the odds of adherence in the Obstetrics and Gynaecology (OBGY) wards was 64% lower than in surgical wards (aOR = 0.36, 95% CI: 0.2–0.7, p = 0.003)” and instead refer readers to Table 2.

15. I don't quite understand the first sentence of the Discussion. I am not clear what there is "limited evidence of" and whether it is true to say the evidence is limited i.e., no reference to review literature etc. Suggest clarifying.

**Response**: We have amended the opening sentences of the discussion section (lines **411-413**):

“Antimicrobial prescribing guidelines are considered to be fundamental to good antimicrobial prescribing practice; however, there is limited evidence comparing the impact of different delivery formats, particularly paper-based and app-based guidelines on adherence.”

16. Otherwise, the Discussion is very well-written and clear. Well done!

**Response**: Thank you.

17. I think the authors are trying to fit too much into Figure 1 - I found it very difficult to read. Suggest simplifying and focussing on the primary purpose of the figure: communicating the structure of the study.

**Response**: Thank you for the suggestion. We recognise that the figure was dense, as it combined data from both hospitalised patients and outpatients. To improve clarify, we have separated them into two figures, presenting hospitalised patients (Fig 1) and outpatients (S2 Fig) separately.

18. [MINOR] For future reference I suggest lodging your SAP in an independent online archive like medRxiv as proof the SAP was completed prior to the study finishing.

**Response**: Thank you for your suggestion. Now the SAP is available as S3 text and under: <https://doi.org/10.6084/m9.figshare.32016120>

**Reviewer #3:**

Thank you for the opportunity to review this well written and important paper. 

1. The need for effective stewardship is stark across several metrics- including the high percentage of inpatients receiving at least one, if not two antibiotics, and the low rate of adherence to guidelines. The paper highlights prophylaxis as an area of particular difficulty. Is there documentation of who attended training? Was the attendance among surgical and obstetric/gynae prescribers as high as on the medical/ICU wards? And was prophylaxis a focus of the training?

**Response**: Thank you for raising this important point. We agree that clarification is needed. Detailed information on training attendance was not included in the original manuscript; this has now been added to the Supplementary Material (see S2 Table and S3 Table), including documentation of participants across departments. Attendance from obstetrics and gynaecology and surgical wards accounted for 45 (13.1%) and 44 (12.8%) participants, respectively, while the highest participation was from medical wards (77; 22.4%), followed by paediatric wards (30; 8.7%). We have also added brief information about prescribers and directed the readers to the supplementary material for further details (lines **259-261**):

“During the study period, 383/507 (75.5%; 95% CI: 71.6-79.2) prescribers had access to paper-based guidelines, while 362/477 (75.9%; 95% CI: 71.8-79.7) had access to the app-based guidelines (S2 Table and S3 Table).”

The training was based on the antimicrobial prescribing guidelines and covered general principles of good antibiotic prescribing and antimicrobial stewardship. Elective surgical prophylaxis was included as part of the training content; however, the focus was on overall prescribing practices rather than on any specific topic.

2. Presumably the paper guidelines were still accessible during the intervention period? Is there any information about how/when these were accessed, or e.g. whether the prescribers still had legible/complete copies when the surveys were carried out?

**Response**: Thank you for this question. Yes, prescribers continued to have access to the paper-based guidelines during the intervention period. We collected information on the use of the paper-based guidelines, but not on their condition. However, these data are not presented in the current manuscript to maintain focus and readability, given the already substantial volume of results

3. I'm not that convinced by the argument in lines 399-400. I know the qualitative outcomes are presented elsewhere, but the data about the roughly monthly accessing of the app per prescriber appears to belie it being used as an everyday clinical decision support tool. For me the main thrust of the paper is the low adherence to guidelines and that alternative/additional strategies need to be pursued in specific contexts. While guidelines may be great for AMS in some settings, it appears that here other approaches will be needed. The qualitative data and potentially some process evaluation metrics could be very helpful- I agree with the further research recommended in lines 429-430.

**Response**: Thank you for this comment. We agree that the relatively infrequent use of the app suggests it was not consistently used as an everyday clinical decision support tool in this setting. We have revised the discussion to better reflect this interpretation and to avoid overstating the impact of the app-based guideline. Please see lines **428-431**:

“Although this increase in adherence was not substantial, it suggests that structured easily accessible antimicrobial use guidelines may support prescribing. However, the relatively infrequent app use indicates that it was not used consistently as a routine clinical support tool in this setting.”

We have also emphasised that guideline implementation alone may be insufficient to substantially change prescribing behaviour, and that additional or alternative antimicrobial stewardship strategies are likely required in this context, please see lines 530-535 in the conclusion section:

“These findings suggest that guidelines alone are insufficient to ensure appropriate antimicrobial use. The urgent priority is to tackle the broader, multifaceted barriers, including improving guidelines awareness, ensuring access to appropriate technology and skills, and strengthening healthcare services in the context of human resource constraints. Regular feedback and sustained support from senior hospital leadership and the Ministry of Health is also essential to promote consistent guideline use and improve patient outcomes.”

4. I'd also want more emphasis on the lack of available diagnostics- almost all treatment is empirical which again impedes effective AMS. 
**Response**: Thank you for this important comment. We agree that access to and appropriate use of microbiological diagnostics are an important component of antimicrobial stewardship, as they support more targeted prescribing. At the same time, most guidelines are commonly used for empiric prescribing since most microbiological diagnostic tests are culture-based and take time to come back. We have referenced this in the discussion (lines **452-456**):

“This suggests that prescribing decisions are driven not only by guideline availability but also by established clinical routines and contextual factors within routine practice. Lack of access to diagnostics for many infections or AMR also impedes antimicrobial stewardship. These findings suggest that, in this setting, prescribing behaviour may be shaped by established clinical habits and border contextual factors, highlighting the potential need for additional strategies to support effective antimicrobial stewardship.”

5. I think the first two sentences of the conclusion are somewhat overstated- I'm not sure the data about app usage, or the slight increase in adherence to guidelines, from a low baseline, indicates successful implementation. For me I'd emphasise the low adherence overall and the relative lack of impact in comparison to other settings, focusing on the need for nuanced, context-specific interventions for effective AMS.

**Response**: Thank you for your comment. We have softened the opening sentences of the Conclusion section (lines **527-530**):

“This study demonstrates that technology-based delivery of antimicrobial prescribing guidelines is feasible in a low-resource setting, such as Laos, with potential to improve healthcare quality in the long term. However overall guideline adherence remained low, and there was insufficient evidence that using a smartphone application to deliver prescribing guidelines had a greater impact on adherence compared to the paper-based guidelines.”

We have also revised the conclusion section to place greater emphasis on the need for nuanced, context-specific interventions for effective antimicrobial stewardship. This includes highlighting the importance of strengthening stewardship programmes and other complementary strategies, as outlined in the latter part of the conclusion

**Minor comments**
6. Lines 165-168- it would be helpful to have more description about the sites- perhaps in an appendix, e.g. bed numbers, catchment areas, rural/urban to be better able to interpret the prescribing figures.

**Response**: we have added an S1 Table providing further information about the study sites, alongside the S1 Fig (Map) of study sites

7. Line 276 I'd rephrase line 276: "Among the 319 prescriptions that could not be assessed" to make it clear that the following sentences refer to those 319.

**Response**: We have amended as suggested (line **291**):

“Among the 319 prescriptions that could not be assessed, 21.9% (70/319) lacked clear prescribing …“

8. Line 282- the 4-6 months of exposure here is to the intervention, not the paper guidelines also? 
**Response**: Thank you for highlighting the lack of clarity in this sentence. We have clarified this part (lines **296-297**):

“After all hospitals had access to the paper-based guidelines for approximately 4-6 months, surveys were conducted across all hospitals to assess adherence.”

9. Line 332-333 "While the use in the PP-G group was 1,267/1,492 (84.9%, 95% CI: 83.0-86.7), and in the App-G group was 943/1,217 (77.5%; 95% CI: 75.0-79.8)." This doesn't appear to be a full sentence
**Response**: We have amended this section to improve clarity (lines **352-354**):

“In the pre-guidelines group, 733/852 (86.0%; 95% CI: 83.5-88.3) received IV antimicrobials. IV antimicrobial use was 1,267/1,492 (84.9%, 95% CI: 83.0-86.7) in the PP-G group, and 943/1,217 (77.5%; 95% CI: 75.0-79.8) in the App-G group.”

10. Lines 487-489 about paper guidelines- this would fit better in the discussion than the conclusion.
**Response**: Thank you for your recommendation, we have moved this part in the conclusion to the discussion section (lines **497-501**):

“This digital divide can result in lower utilization of app-based guidelines; therefore, the prescribers might have continued using the traditional paper-based version rather than switching to app-based version. However, paper-based guidelines present significant challenges, such as difficulties in nationwide dissemination, delays in updating, and the costs associated with printing and dissemination, especially when compared to app-based guidelines.”

11. The conclusion is quite lengthy- I'd move some of the discussion about resource constraints to the discussion also.

**Response**: Thank you very much for your suggestion. We have revised it by removing some repetition while retaining the original key messages. Please see the revised conclusion section (lines **527-535**):

“This study demonstrates that technology-based delivery of antimicrobial prescribing guidelines is feasible in a low-resource setting, such as Laos, with potential to improve healthcare quality in the long term. However overall guideline adherence remained low, and there was insufficient evidence that using a smartphone application to deliver prescribing guidelines had a greater impact on adherence compared to the paper-based guidelines. These findings suggest that guidelines alone are insufficient to ensure appropriate antimicrobial use. The urgent priority is to tackle the broader, multifaceted barriers, including improving guidelines awareness, ensuring access to appropriate technology and skills, and strengthening healthcare services in the context of human resource constraints. Regular feedback and sustained support from senior hospital leadership and the Ministry of Health is also essential to promote consistent guideline use and improve patient outcomes.”

12. Figure 1 is too small- I only find it legible when I've magnified it by ~300%. It might fit better in portrait? I'd prefer to have the absolute figures and % rather than 95% CIs in this figure.
**Response**: We recognise that the figure 1 was dense and difficult to read because it combined data from both hospitalised patients and outpatients. We have now separated these into two figures, presenting each group individually (Fig 1 for hospitalised patients and S2 Fig for outpatients) to improve readability and clarity.

13. Supplementary figure 9: are the figures in brackets 95% CIs? 
**Response**: Thank you for pointing this out. That’s correct. We have added “% (95% CI)” at the end of the title for better clarification: S6 Fig (this has now been changed from S9 Fig to S6 Fig): Percentage of antimicrobial prescriptions fully adherent to prescribing guidelines among hospitalised patients, % (95% CI)

14. Supplementary figure 11: I don't understand this table- is this a comparison between app- and paper-based guideline groups or more general comparisons? It looks and reads like it is an interdepartmental comparison for example, rather than comparing between departments and guideline groups? And is it about antimicrobial usage altogether or adherence to guidelines? So, is it saying that fewer antibiotics were used in medicine, or that there was poorer adherence? And similarly, was there better adherence, or more antimicrobial use in Sequence 2? 
What are VIP departments? 
**Response**: Thank you for this helpful comment. We apologise for the lack of clarity. This table presents a multivariable (mixed-effects logistic regression) analysis examining **antimicrobial use,** not adherence to prescribing guidelines. The primary comparison is between the app-based (App-G) and paper-based (PP-G) guideline groups, with adjustment for sequence of intervention, department, time, and clustering. The table therefore presents both the comparison between guideline delivery methods and other covariates within the same model.

Lower adjusted odds ratios in Medicine indicate lower odds of antimicrobial use compared to Surgery, while the higher odds in Sequence 2 indicates higher odds of antimicrobial use compared to Sequence 3. These do not reflect adherence.

VIP ward in Laos refers to a hospital ward providing upgraded or special services, typically including private rooms and additional amenities for patients who can afford to pay. It is not a clinical specialty ward; rather, it may include patients from different specialties such as Obstetrics and Gynaecology, Surgery, or Medicine. We have added an explanation in the footnote of the table: “a ward with enhanced service (e,g., private rooms) for patients who can afford to pay”

15. Supplementary figure 12: There is a lot of variability here- e.g. in the Luang Namtha site. Can you explain this? Is DDD perhaps not the best measure here?

**Response**: Thank you for this important comment. We agree that there is variability in DDD values across sites and periods, particularly in Luang Namtha. This likely reflects a combination of differences in antibiotic use patterns, small numbers of patient-days in some hospitals, variability in prescribing practices across hospitals and time periods, and case-mix.

We acknowledge that DDD per 100 patient-days has limitations in this setting. While it is a widely used WHO-standardised metric that enables comparison across hospitals and time, it may be less stable in smaller hospitals and more sensitive to fluctuations in patient volume and case-mix. However, we retained DDD as it is a well known and standardised measure available for comparing antibiotic consumption across multiple hospitals in this study.

16. Supplementary figure 15: It might be easier to see the comparison between the two groups if 15.1 and 15.2 were combined with the bars on the same x axis
**Response**: We have combined as suggested for both inpatients and outpatients, please see S4 Fig (inpatients) and S7 Fig (outpatients)

17. Supplementary figure 19: looking at the timing of access- did this correspond with tea and lunch breaks? Do people find it hard to look at their phones at the time of patient review and check afterwards/before? 
**Response**: Thank you for raising this interesting point. In Laos, usual doctor consultation times are between 8–10am, and some departments also operate later in the day, usually from 1–3pm. This has been mentioned in lines **401–402**:

“Cumulative app data showed peak access between 8-10am and around 1-2pm, highest at 9am, aligning with typical consultation hours”

18. Supplementary figure 20: It doesn't look like the numbers here add up to the total number of searches/accessing of the app (6,239 times). What is the reason for the discrepancy? 
**Response**: The cumulative app access was 6,239 times, and the cumulative number of searches was 527 during the 22-month study period. The app includes a function that captures how often users type into the search menu to find information, and this is what is presented in the graph. Although there were 527 searches, many users navigated directly to the topics they needed, as they were familiar with the guideline structure. These were counted as “access” rather than “search.” However, when users performed a search, this was also counted as an access. We have clarified this in lines **403-406**:

“The cumulative app searches during the study period were 527 times, and the most frequently searched topic was melioidosis, followed by pneumonia and antimicrobial agents (S10 Fig and S11 Fig). Searches reflect use of the search function, while direct navigation was counted as access.”

19. Supplementary- typos in the CONSORT table- Methods not methos

**Response**: Thank you, we have corrected all three typos.

**General editorial requests:**

(Note: not all will apply to your paper, but please check each item carefully)

* We ask every co-author listed on the manuscript to fill in a contributing author statement, making sure to declare all competing interests. If any of the co-authors have not filled in the statement, we will remind them to do so when the paper is revised. If all statements are not completed in a timely fashion this could hold up the re-review process. If new competing interests are declared later in the revision process, this may also hold up the submission. Should there be a problem getting one of your co-authors to fill in a statement we will be in contact. Please do not add or remove authors without first discussing this with the handling editor. You can see our competing interests policy here: http://journals.plos.org/plosmedicine/s/competing-interests.

* Please upload any figures associated with your paper as individual TIF or EPS files with 300dpi resolution at resubmission; please read our figure guidelines for more information on our requirements: http://journals.plos.org/plosmedicine/s/figures. While revising your submission, we strongly recommend that you use PLOS's NAAS tool (https://ngplosjournals.pagemajik.ai/artanalysis) to test your figure files. NAAS can convert your figure files to the TIFF file type and meet basic requirements (such as print size, resolution), or provide you with a report on issues that do not meet our requirements and that NAAS cannot fix.
**Response**: Thank you for your recommendation. We have used PLOS’s NAAS to revise our figures as suggested

After uploading your figures to PLOS's NAAS tool - https://ngplosjournals.pagemajik.ai/artanalysis, NAAS will process the files provided and display the results in the "Uploaded Files" section of the page as the processing is complete.

If the uploaded figures meet our requirements (or NAAS is able to fix the files to meet our requirements), the figure will be marked as "fixed" above. If NAAS is unable to fix the files, a red "failed" label will appear above.

When NAAS has confirmed that the figure files meet our requirements, please download the file via the download option, and include these NAAS processed figure files when submitting your revised manuscript.

* Please ensure that the paper adheres to the PLOS Data Availability Policy (see http://journals.plos.org/plosmedicine/s/data-availability), which requires that all data underlying the study's findings be provided in a repository or as Supporting Information. For data residing with a third party, authors are required to provide instructions with contact information (web or email address) for obtaining the data. Please note that a study author cannot be the contact person for the data. PLOS journals do not allow statements supported by "data not shown" or "unpublished results." For such statements, authors must provide supporting data or cite public sources that include it.

Response: I have included a data sharing and open access section in the manuscript (lines **544-548**):

“The data cannot be shared publicly because they are owned by a third party (the Ministry of Health). Access to the data may be requested through the Mahidol-Oxford Tropical Medicine Research Unit Data Access Committee. All data access requests and related enquiries should be submitted to datasharing@tropmedres.ac. For open access, the author has applied a CC BY public copyright licence to any Author Accepted manuscript version arising from this submission.”

* We expect all researchers with submissions to PLOS in which author-generated code underpins the findings in the manuscript to make all author-generated code available without restrictions upon publication of the work. In cases where code is central to the manuscript, we may require the code to be made available as a condition of publication. Authors are responsible for ensuring that the code is reusable and well documented. Please make any custom code available, either as part of your data deposition or as a supplementary file. Please add a sentence to your data availability statement regarding any code used in the study, e.g. "The code used in the analysis is available from Github [URL] and archived in Zenodo [DOI link]" Please review our guidelines at https://journals.plos.org/plosmedicine/s/materials-software-and-code-sharing and ensure that your code is shared in a way that follows best practice and facilitates reproducibility and reuse. Because Github depositions can be readily changed or deleted, we encourage you to make a permanent DOI'd copy (e.g. in Zenodo) and provide the URL. 
**Response**: STATA syntax for sample size calculation and primary analysis model was provided via S1 Text and at <https://doi.org/10.6084/m9.figshare.32051097>

* Please ensure that the study is reported according to the CONSORT guideline and include the completed CONSORT checklist as Supporting Information. When completing the checklist, please use section and paragraph numbers, rather than page numbers. Please add the following statement, or similar, to the Methods: "This study is reported as per [XXXX] guideline (S1 Checklist)."

**Response**: The CONSORT Checklist is available as S2 Checklist

**FORMATTING - GENERAL**
* Abstract: Please structure your abstract using the PLOS Medicine headings (Background, Methods and Findings, Conclusions). Please combine the Methods and Findings sections into one section.
**Response**: This has already been addressed in the manuscript

* At this stage, we ask that you include a short, non-technical Author Summary of your research to make findings accessible to a wide audience that includes both scientists and non-scientists. The Author Summary should immediately follow the Abstract in your revised manuscript. This text is subject to editorial change and should be distinct from the scientific abstract. Ideally each sub-heading should contain 2-3 single sentence, concise bullet points containing the most salient points from your study. In the final bullet point of 'What Do These Findings Mean?', please include the main limitations of the study in non-technical language. Please see our author guidelines for more information: https://journals.plos.org/plosmedicine/s/revising-your-manuscript#loc-author-summary.
**Response**: This has already been addressed in the manuscript

* Please express the main results with 95% CIs as well as p values. When reporting p values please report as p<0.001 and where higher as the exact p value p=0.002, for example. Throughout, suggest reporting statistical information as follows to improve clarity for the reader "22% (95% CI [13%,28%]; p</=)". Please be sure to define all numerical values at first use.
**Response**: This has already been addressed in the manuscript

* Please include page numbers and line numbers in the manuscript file. Use continuous line numbers (do not restart the numbering on each page). 
**Response**: This has already been addressed in the manuscript

* Please cite the reference numbers in square brackets. Citations should precede punctuation.
**Response**: This has already been addressed in the manuscript

**FIGURES AND TABLES**
* Please provide titles and legends for all figures and tables (including those in Supporting Information files).
**Response**: We have provided all titles and legends after reference section

* Please define all abbreviations used in each figure/table (including those in Supporting Information files).
**Response**: we have revised and provided the full name of abbreviations where applicable

* Please consider avoiding the use of red and green in order to make your figure more accessible to those with color blindness.
**Response**: We have amended some figures. In some figures, labels and ordering from highest to lowest values are used to improve clarity

**SUPPLEMENTARY MATERIAL**
* Please note that supplementary material will be posted as supplied by the authors. Therefore, please amend it according to the relevant comments outlined here.

* Please cite your Supporting Information as outlined here: https://journals.plos.org/plosmedicine/s/supporting-information
**Response**: we have amended based on the supporting information guidelines

**REFERENCES**

* PLOS uses the numbered citation (citation-sequence) method and first six authors, et al.
**Response**: This has already been addressed in the manuscript

* Please ensure that journal name abbreviations match those found in the National Center for Biotechnology Information (NCBI) databases (http://www.ncbi.nlm.nih.gov/nlmcatalog/journals), and are appropriately formatted and capitalised.
**Response**: This has already been addressed in the manuscript

* Where website addresses are cited, please include the complete URL and specify the date of access (e.g. [accessed: 12/06/2023]).
**Response**: This has already been addressed in the manuscript

* Please also see https://journals.plos.org/plosmedicine/s/submission-guidelines#loc-references for further details on reference formatting.

**STUDY TYPE-SPECIFIC REQUESTS:**

RCTs 

* PLOS Medicine requires that all trials be prospectively registered in one of registries recognized by WHO. Please ensure that study registration details are included in the Methods section.
**Response**: We have included detail of trial registration in the methods (line **157**): This trial was registered at clinicaltrials.gov (NCT04914793)

* Please provide explanation for the retrospective registration in the Methods section
Response: Please see lines **157-161**:

“The trial was registered a few months after commencement due to an initial interpretation of registration requirements. As this was a cluster randomised controlled trial without individual participant randomisation and evaluated the impact of antimicrobial prescribing guidelines rather than direct health outcomes, registration was not initially undertaken. Following further consultation, a conservative approach was adopted and the trial was subsequently registered.”

* Please structure the Methods section using the following sub-headings: Study design and participants, Randomization and masking, Procedures, Outcomes, Statistical analysis.
**Response**: This has already been addressed in the manuscript

* The following outcomes measures - analysis of the hospitalized and outpatients as a single group or separately - it is not very clear and appear to differ between the submitted manuscript and the protocol [and/or trial registry]. Please clarify or explain all discrepancies between the paper and protocol. If the outcomes were not prespecified in the protocol, please define them in the Methods (Outcomes section) as post hoc and explain why they were added. Post-hoc comparisons should be presented as hypothesis generating rather than conclusive.

* Please ensure that all prespecified outcomes (primary, secondary, and exploratory) are listed in the Methods/Outcomes section and indicate whether there are outcomes that are not presented in the current report.
**Response**: All outcomes were prespecified

* Please specify the dates (Month Day, Year) during which study enrollment and follow up occurred.
**Response**: This has already been addressed in the manuscript

* Please include absolute numbers wherever you report percentages; eg, n/N (%)
**Response**: This has already been addressed in the manuscript

* Please present the safety data for the study including numbers of specific events and whether or not adverse events are thought to be related to treatment. AEs should be reported in the abstract, per CONSORT and CONSORT-Harms.

**Response**: This is not applicable to this trial. We have also mentioned this in the CONSORT Checklist

* Please complete the CONSORT checklist (https://www.equator-network.org/reporting-guidelines/consort/) and ensure that all components of CONSORT are present in the manuscript, including how randomization was performed, allocation concealment, blinding of intervention, definition of lost to follow-up, power statement. When completing the checklist, please use section and paragraph numbers, rather than page numbers.

**Response**: We have completed the CONSORT checklist ( S2 Checklist)

* Please report your abstract according to CONSORT for abstracts, following the PLOS Medicine abstract structure (Background, Methods and Findings, Conclusions) https://www.equator-network.org/reporting-guidelines/consort-abstracts/
**Response**: This has already been addressed in the manuscript

* If your trial had to undergo important modifications in response to extenuating circumstances, please complete the CONSERVE-CONSORT checklist and provide in your Supporting Information; (https://www.equator-network.org/reporting-guidelines/guidelines-for-reporting-trial-protocols-and-completed-trials-modified-due-to-the-covid-19-pandemic-and-other-extenuating-circumstances-the-conserve-2021-statement/). When completing the checklist, please use section and paragraph numbers, rather than page numbers.
**Response**: This is not applicable to this trial.

* In keeping with our commitment to Open Science, please include the study protocol document and analysis plan (including any amendments) as Supporting Information to be published with the manuscript if accepted.

**Response**: The Statistical Analysis Plan is available as S3 Text (Statistical Analysis Plan, SAP) and is also publicly accessible at <https://doi.org/10.6084/m9.figshare.32016120>. The study protocol is currently publicly available at <https://doi.org/10.6084/m9.figshare.32051121>.

* Please note that PLOS Medicine requires prospective, public registration of a data sharing plan (as part of mandatory clinical trials registration) for all clinical trials that began enrollment on or after January 1, 2019, in accordance with ICMJE requirements.
