# Supplementary material for: Adherence to smartphone-delivered antimicrobial prescribing guidelines in hospitals in Lao PDR: A stepped-wedge cluster randomized controlled trial
Source: PLoS Med. 2026 Aug 27;23(8):e1005205. doi: 10.1371/journal.pmed.1005205 (PMC13521649; doi:10.1371/journal.pmed.1005205)
Supplement: Supplementary file 18 [file pmed.1005205.s030.docx]

**Response to reviewers and editors (date: 29 June 2026)**

Dear Reviewers and Editors,

Re: Assessment of adherence to antimicrobial prescribing guidelines delivered by a smartphone application in hospitals in Lao PDR: Stepped-wedge cluster randomised controlled trial (PMEDICINE-D-25-03563R3)

We would like to thank you for your time and effort in reviewing this manuscript. Please find our point-by-point responses to your questions and comments below.

**Comments from the reviewers:**

**Reviewer #2:** I thank the authors for their considered and comprehensive responses to my comments and note the manuscript is reading very well. In all but two cases, I am happy to accept the authors proposed updates and consider the items finalised. However, I do suggest the authors revisit the two items below (numbers reference my original review items).

1. I think the logic in the authors response to my review item is sound, but it does not appear the manuscript was updated accordingly. I suggest incorporating the logic in the response into the manuscript. In particular, making clear that analysis of all prescribing during the study was not possible due to the absence of electronic records, so PPSs were used, with appropriate citation(s) to demonstrate acceptability of this approach.

**Response**: Thank you for your suggestion. We have added more details on the rationale for using Point Prevalence Surveys (PPSs) in the manuscript. We have revised the Methods section to clarify that continuous collection of prescribing data throughout the study was not feasible because electronic prescribing records were not routinely available in Lao hospitals. PPSs were used as a practical and standardised approach to measure antimicrobial prescribing and guideline adherence in this low-resource setting. We have also added a reference supporting the use of PPS methodology for antimicrobial use surveillance, including in low- and middle-income countries.

These revisions have been incorporated into the Methods section, lines **159-162** with a citation:

“Outcome data were collected using point prevalence surveys. PPSs are widely used to monitor antimicrobial use and prescribing practices and provide a standardized and feasible method for antimicrobial surveillance and stewardship activities, particularly in LMICs, where electronic prescribing records are not routinely available, and continuous data collection is otherwise costly and time-consuming [21].”

Reference: 21. Global Point Prevalence Survey. *Local actions around the world - Countries participating or enrolled in the Global-PPS*. 2026 [cited 12 June 2026]. Available from: <https://www.global-pps.com/>.

6. The authors response to my comment and their adjustment to the manuscript are all good apart from one omission. Given the authors are relying on PPSs rather than using all prescribing data, I think it's important to reinforce that while the timeframes shifted away from the planned 4-month intervals, even where delays occurred the PPS was always conducted before each hospital transitioned to the intervention.

**Response**: Thank you for this suggestion. We have revised the methos section to clarify this point (lines **288-294**):

“The trial operated over 22 months, from January 2021 to October 2022. The first group of hospitals (XK and LNT) received the intervention in June-July 2021 (sequence-1), the second group (SV and SVK) in November-December 2021 (sequence-2), and the last group (VTP and MS) in April 2022 (sequence-3), with five-to-six-month intervals between each. This was longer than expected due to COVID-19 pandemic restrictions. However, each PPS was completed before hospitals transitioned to the next phase, ensuring that outcome data were collected before exposure to the intervention (Fig 1 for hospitalised patients and S2 Fig for outpatients)”

**Reviewer #3**: Thank you for your comprehensive response and rebuttal to reviewer comments. I have no further comments except: I'm supportive of the point prevalence survey methodology as a pragmatic option in low-resource settings where daily prescribing data is likely to be sparse and challenging to access if available at all.

Thank you for redoing Figure 1- it's much clearer now.

**Response**: Thank you for your positive comments and support for the methodology used in this study.

**EDITORIAL COMMENTS**

* Thank you for providing the Author Summary. The Author Summary should immediately follow the Abstract in your revised manuscript. This text is subject to editorial change and should be distinct from the scientific abstract. Ideally each sub-heading should contain 2-3 single sentence, concise bullet points containing the most salient points from your study. In the final bullet point of ‘What Do These Findings Mean?’ Please include the main limitations of the study in non-technical language.
Please see our author guidelines for more information: [https://journals.plos.org/plosmedicine/s/revising-your-manuscript#loc-author-summary](https://url.uk.m.mimecastprotect.com/s/1CIKCqVRJC8EE8WCWUkcEvbBL?domain=journals.plos.org).

**Response**: Thank you for your suggestion. Our Author Summary has been placed immediately after the Abstract, and we have added a statement describing the main study limitation in the “What do these findings mean?” section (lines **113-115**):

“The study assessed prescribing using patients' medical records. Prescriptions could not be reliably linked to individual prescribers. As a result, we could not assess changes in prescribing behaviour at the individual prescriber level after the guidelines were introduced.”

* Please confirm that your title complies with PLOS Medicine's style. Your title must be nondeclarative and not a question. It should begin with main concept if possible. "Effect of" should be used only if causality can be inferred, i.e., for an RCT. Please place the study design ("A randomized controlled trial," "A retrospective study," "A modelling study," etc.) in the subtitle (ie, after a colon).
**Response**: We have amended the title to make it more concise and clearer, the updated title can be found on page 1:

“Adherence to smartphone-delivered antimicrobial prescribing guidelines in hospitals in Lao PDR: A stepped-wedge cluster randomized controlled trial”

* Please confirm that your abstract complies with our requirements, including format (three sections: Background, Methods and Findings, and Conclusions) and providing all the information relevant to this study type [https://journals.plos.org/plosmedicine/s/submission-guidelines#loc-abstract](https://url.uk.m.mimecastprotect.com/s/lE0aCrEZKF8kk89C0cXc4jnwC?domain=journals.plos.org)
**Response**: Thank you for your suggestion. We follow the link you shared and we have added the main limitations in our “Methods and Findings” section (lines **75-77**):

“Prescribing in this study was assessed using patients' medical records; as prescriptions could not be reliably linked to individual prescribers, changes in individual prescribing behaviour could not be evaluated.”

* Please ensure that the Introduction ends with a clear description of the study question or hypothesis.

**Response**: Thank you, we have amended the final paragraph of the introduction to improve the clarity (lines **142-146**):

“The national antimicrobial prescribing guideline for Laos were developed between 2019 and 2020, in both paper-based and smartphone application-based formats. Dissemination of these guidelines began in 2021. This trial compared the proportion of antimicrobial prescriptions adhering to the guidelines delivered via smartphone app versus the paper-based version among inpatient and outpatient settings in six hospitals in Laos.”

* Please ensure that all abbreviations are defined at first use throughout the text.
**Response**: We have checked the manuscript and confirmed that all abbreviations are defined at first use throughout the text

* Please confirm that all numbers presented in the abstract are present and identical to numbers presented in the main manuscript text.

**Response**: We confirmed that the numbers presented in the abstract are identical to those numbers reported in the main text of the manuscript.

**GENERAL**
* Please disclose any use of AI tools and technologies to the study or article content at the end of the Methods. Please specify the name(s) and version(s) of any tool(s) used and a description of their use, including how the AI-assisted or generated content was evaluated by authors. Please also include a statement specifying who retains responsibility for the final manuscript. Please see our guidelines for more information: [https://journals.plos.org/plosmedicine/s/ethical-publishing-practice#loc-artificial-intelligence-tools-and-technologies](https://url.uk.m.mimecastprotect.com/s/EUQ1CvYqOC7YY7XHrfWcQXUWA?domain=journals.plos.org).
**Response**: Thank you for raising this important point. We confirm that AI was used only to improve grammar and language editing. We have added a short statement to clarify this at the end of the Methods section (lines **277-278**):

“ChatGPT (OpenAI; GPT-5.5) was used only to support grammar and language editing. All content was checked and revised by the authors, who are fully responsible for the final manuscript.”

* Please review your text for claims of novelty or primacy (e.g. 'for the first time') and remove this language. In addition, please check that any use of statistical terms (such as trend or significant) are supported by the data, and if not please remove them.
**Response**: We have reviewed the entire text and removed claims of novelty or primacy. For example, we revised (lines **58-59**) from:

“Following the release of the first Lao national antimicrobial prescribing guidelines in 2021”

to

“Following the release of the Lao national antimicrobial prescribing guidelines in 2021”

We have reviewed the manuscript and confirmed that we did not use statistical terms which were not supported by the data.

* Please remove the 'conclusions' subheading from the discussion. Please also remove any other subheadings from the discussion.

**Response**: We have removed it.

* Statistical reporting: Please revise throughout the manuscript, including tables and figures.
- Please report statistical information as follows to improve clarity for the reader ""22% (95% CI [13,28]; p</=)"".
- Please separate upper and lower bounds with commas instead of hyphens as the latter can be confused with reporting of negative values.
- Please repeat statistical definitions (HR, CI etc.) for each set of parentheses.
**Response**: Thank you for your suggestions. We have amended the confidence interval format throughout the manuscript and supplementary materials, and revised the manuscript to ensure that statistical abbreviations are defined when first used in each relevant section.

* In the last sentence of the Abstract Methods and Findings section, please describe the main limitation(s) of the study's methodology.
**Response**: Thank you, we have added as suggested (lines **75-77**):

“Prescribing in this study was assessed using patients' medical records; as prescriptions could not be reliably linked to individual prescribers, changes in individual prescribing behaviour could not be evaluated.”

* In the abstract, please include the important dependent variables that are adjusted for in the analyses.

**Response**: Thank you, we have added as suggested (lines **69-71**):

“After accounting for time and cluster effects and adjusting for confounders (sequence, age, gender, prescribing indication and hospital department), adherence did not differ by guideline delivery mode … “

**FUNDING STATEMENT**
* The funding statement should include: specific grant numbers, initials of authors who received each award, URLs to sponsors’ websites. Also, please state whether any sponsors or funders (other than the named authors) played any role in study design, data collection and analysis, the decision to publish, or preparation of the manuscript. If they had no role in the research, include this sentence: “The funders had no role in study design, data collection and analysis, decision to publish, or preparation of the manuscript.”
**Response**: Thank you for your suggestion. We have amended this section as suggested (lines **274-276**):

“This study was funded by the Wellcome Trust of Great Britain [Grant number: 214207/Z/18/Z] awarded to VC. The funder’s website is https://wellcome.org/. The funder had no role in study design, data collection and analysis, decision to publish, or preparation of the manuscript.”

**COMPETING INTERESTS STATEMENT**
* All authors must declare their relevant competing interests per the PLOS policy, which can be seen here: [https://journals.plos.org/plosmedicine/s/competing-interests](https://url.uk.m.mimecastprotect.com/s/vcSECwE9PFGqqGpULhycJH8Jd?domain=journals.plos.org) For authors with ties to industry, please indicate whether any of the interests has a financial stake in the results of the current study.

**Response**: We have revised the competing interests statement to improve the clarity (lines **570-571**):

“EAA is a member of the Editorial Board of PLOS Medicine. The remaining authors declare that they have no competing interests.”

*Please amend your competing interests statement. Funding information should go to the relevant section and include URLs.

**Response**: We have revised the manuscript and confirmed that funding information is presented in the relevant section and includes URL, as suggested (lines **274-276**):

“This study was funded by the Wellcome Trust of Great Britain [Grant number: 214207/Z/18/Z] awarded to VC. The funder’s website is https://wellcome.org/. The funder had no role in study design, data collection and analysis, decision to publish, or preparation of the manuscript.”

**DATA AVAILABILITY**
* PLOS Medicine requires that the de-identified data underlying the specific results in a published article be made available, without restrictions on access, in a public repository or as Supporting Information at the time of article publication, provided it is legal and ethical to do so. Please see the policy at [http://journals.plos.org/plosmedicine/s/data-availability](https://url.uk.m.mimecastprotect.com/s/RL5rCxVqQC1771OCBi9cyqPCf?domain=journals.plos.org) and FAQs at
[http://journals.plos.org/plosmedicine/s/data-availability#loc-faqs-for-data-policy](https://url.uk.m.mimecastprotect.com/s/g5nFCy6lRHr88rnC6s9cxSvp1?domain=journals.plos.org)

**Response**: Thank you for highlighting the Data Availability Policy. We have deposited a de-identified analysis dataset containing the variables underlying the results reported in this manuscript in Figshare. This dataset includes all variables required to reproduce the analyses presented in the manuscript while excluding variables that were not used in the reported analyses or cannot be shared for confidentiality reasons. We have updated the Data Availability Statement accordingly and included the Figshare DOI in the revised manuscript (lines **574-582**):

“A de-identified dataset containing the variables used in the analyses presented in this manuscript is publicly available on Figshare (<https://doi.org/10.6084/m9.figshare.32812736>). The full original study dataset is owned by a third party (the Ministry of Health). Access to the data may be requested through the Mahidol-Oxford Tropical Medicine Research Unit Data Access Committee. All data access requests and related enquiries should be submitted to [datasharing@tropmedres.ac](mailto:datasharing@tropmedres.ac).

The code used for sample size calculations and statical analyses is available at S1 Text and https://doi.org/10.6084/m9.figshare.32051097.

For the purpose of open access, the author has applied a CC BY public copyright licence to any Author Accepted manuscript version arising from this submission.”

* PLOS defines the “minimal data set” to consist of the data set used to reach the conclusions drawn in the manuscript with related metadata and methods, and any additional data required to replicate the reported study findings in their entirety. Authors do not need to submit their entire data set, or the raw data collected during an investigation. Please submit the following data:
The values behind the means, standard deviations and other measures reported;

The values used to build graphs;

The points extracted from images for analysis.
**Response**: Thank you for the comment. Following the response above, the listed variables are now available in the dataset with data dictionary on Figshare: (<https://doi.org/10.6084/m9.figshare.32812736>).

* Please include the statement on code availability in the data availability statement. Please see guidance here:[https://journals.plos.org/plosmedicine/s/code-sharing-guidance#loc-data-availability-statement-examples](https://url.uk.m.mimecastprotect.com/s/WOYMCzBP0UMkkMLCrtVc9HMFw?domain=journals.plos.org)
**Response**: We have revised the data availability and open access section to include information on code availability, as requested (lines **579-580**):

“ ….. The code used for sample size calculations and statical analyses is available at S1 Text and <https://doi.org/10.6084/m9.figshare.32051097>. ….”

**ETHICS AND CONSENT**
* Please revise for use of patient-centered language. Please note that patient-centered language is constructed with the use of post-modified nouns (e.g. 'patients with psoriasis’ (or similar) instead of ‘psoriasis patients’) putting the person first in the sentence structure.

**Response**: Thank you for this suggestion. We have reviewed the manuscript and revised the wording where appropriate to use patient-centered language (lines **498-501**):

“For example, among patients with UTI in the USA (2013-2018), only 47% of prescriptions were appropriate (2,762/5,870; 95% CI [45.8,48.3]) [42]. In British Columbia, 44% of 42,452 prescriptions for paediatric outpatients with CAP (2014-2018) were inappropriate [43]. A cluster RCT by Rambaud-Althaus et al. …”

**FIGURES**
* Please ensure that where relevant figures include 95% CIs.
**Response**: We have checked and confirmed that all relevant figures include confidence interval (CI) presented in the appropriate format.

* Please show graph axes beginning at zero. If this is not possible, please show a break in the axis.

**Response**: Thank you, we have reviewed all figures and confirmed that the axes begin at zero.

* Where data points are discrete, please ensure that they are depicted in the figures as discrete data and not as a continuous line.
**Response**: Thank you for this comment. Guideline adherence was measured at discrete survey periods. In the Figure 2 and 3, adherence values are represented as individual data points for each period. The connecting lines are included only to support visualisation of the stepped-wedge rollout and help readers to follow each cluster sequence across study periods. They do not imply continuous measurement between observations. We have also removed the overall connecting line from both figures to avoid misleading readers. In addition, we increased the size of the observed data points and reduce the thickness of the connecting lines to make the discrete observations more clearly distinguishable. To clarify this, we have added the following statement to the figure legend (lines **736-738** for Figure 2 and lines **745-747** for Figure 3):

“Points represent observed adherence at each survey period. Connecting lines are included only to facilitate visualisation of the stepped-wedge rollout and adherence patterns across sequences. They do not represent continuous measurements between observations.”

* Please confirm that the appropriate usage rights apply to the use of this map. Please see our guidelines for map images: [https://journals.plos.org/plosmedicine/s/figures#loc-maps](https://url.uk.m.mimecastprotect.com/s/znNUCAQqyFNwwNYf5uOcGysDK?domain=journals.plos.org)
**Response**: Thank you for this suggestion. We have added a statement in the S1 legend for clarity (lines **804-806**):

“The base layer data for the country and provincial boundaries were obtained from the GADM website: [https://gadm.org/](https://url.uk.m.mimecastprotect.com/s/rnrFC2g46hpBDNjT0FJc5A7hy?domain=gadm.org). The license information for academic use is available at: [https://gadm.org/license.html](https://url.uk.m.mimecastprotect.com/s/L9u3C3jR7Spyr5BTOHRcQ_MbU?domain=gadm.org). The map was created by the research team using QGIS”

* Please provide the unadjusted comparisons as well as the adjusted comparisons in all relevant Tables

**Response**: Thank you for this suggestion. We have added unadjusted comparisons to Table 2 (lines **775**) & Table 3 (lines **787**). To avoid repetition, we have also removed **S7 Table (**Univariate analysis of full guideline adherence and potential confounders among hospitalised patients post-guideline implementation) included in the previous submission, as these results are now presented in the main table.

* Please specify the variables controlled for in all relevant Tables

**Response**: Thank you for your suggestion. We have added details of what the model controlled for in the relevant tables (Table 2, lines [**783-785**]):

“The model was adjusted for sequence, age group, sex, prescribing indication, and hospital department. Indication was the unit of analysis, with random effects for patient and hospital-time.”

Table 3, lines (**796-797**):

“The model was adjusted for sequence and age group. Indication was the unit of analysis, with random effects for patient and hospital-time.”

**INCLUSIVITY IN GLOBAL RESEARCH**
PLOS has a 'Inclusivity in Global Research' policy which aims to promote collaboration and inclusivity in global health research. You are required to complete PLOS’ questionnaire on inclusivity in global research and submit it with your revised paper. The policy and questionnaire can be found at [https://journals.plos.org/plosone/s/best-practices-in-research-reporting](https://url.uk.m.mimecastprotect.com/s/F6sfCB8ozC7ww7oH0Cwc2ubRR?domain=journals.plos.org).

**Response**: Thank you for your suggestion. We have completed the form and included the “Inclusivity in global research” statement in the Methods section (lines **279-281**):

“**Inclusive in global health research**

Additional information regarding the ethical, cultural, and scientific considerations specific to inclusivity in global research is included in the Supporting Information (S2 Checklist).”

**ACKNOWLEDGMENTS**
* Please include an acknowledgment of study participants.
**Response**: Thank you for this reminder, we have added an acknowledgement of the study participants to the Acknowledgements section (lines **585-587**):

“We would like to acknowledge the directors, doctors, and nurses from all participating hospitals for their interest, participation, and continued support. We thank the patients whose data were included in this study. We extend our gratitude to … ”

**CLINICAL TRIALS specific**
* Some of the outcome measures or methods appear to differ between the submitted manuscript and the trial registry and/or protocol. Please clarify and explain the discrepancy.
**Response**: Thank you for this comment. We have checked throughout the method section and added some information to improve clarity (lines **221-231**):

“The outcomes of hospitalised patients were measured using PPS on hospital antimicrobial use. Hospital records of all hospitalised patients of all wards at 8:00 am on the survey day were reviewed. Records documenting the use of antimicrobial agent were included, and total number of inpatients in each ward was recorded to estimate antimicrobial use. Patients admitted after 8:00 am on the survey day were excluded. Patients receiving ongoing antimicrobial regimens (e.g., every 24 hours, every 48 hours or once weekly) were also included. Additional details of PPS methodology for hospitalised patients have been described previously [19]. All outpatient (OPD) records and prescriptions from patients attending the paediatric, medicine, and surgical OPDs of all participating hospitals between 8:00 am and 4:00 pm on the survey days were screened for antimicrobial prescriptions. If the same outpatient turned for consultation during the same survey period, this patient was included in the survey and each visit was defined as a new consultation. Topical antimicrobials were not included in the antimicrobial use calculation for both OPD and hospitalised patients.”

* The following outcomes measures - analysis of the hospitalized and outpatients as a single group or separately - it is not very clear and appear to differ between the submitted manuscript and the protocol [and/or trial registry]. Please clarify or explain all discrepancies between the paper and protocol.

**Response**: Thank you for highlighting this point. We have overlooked this important aspect in the project proposal and the original manuscript. We have now added the relevant information to the main manuscript and revised the text accordingly (lines **271-272**):

“Hospitalised patient data and OPD data were analysed separately. Analyses were performed using STATA 18 …”

* Thank you for attaching SAP to your submission. Although, whole plan is attached could you please provide more detailed information in the methods section of the mansuscript.
Response: Thank you for your suggestion. We have provided more details on Statistical analyses section (lines **255-259**):

“All analyses were done according to intention-to-treat principles. Normally distributed continuous baseline characteristics were summarized with means and standard deviation or 95% confidence intervals. Skewed continuous data were summarized using median and inter-quartile range (± range). Categorical data were presented as frequency and percentages. Test of trends, Chi-square or Fisher’s exact tests were used as appropriate. A mixed-effects logistic regression model …”,

and lines **271-272**:

“Hospitalised patient data and OPD data were analysed separately. Analyses were performed using STATA 18 (StataCorp, College Station, Texas, USA).”

* Please, provide inclusion/exclusion criteria in a manuscript to match the protocol
**Response**: We have provided more details of inclusion/exclusion criteria as suggested (lines **221-232**):

“Hospital records of all hospitalised patients of all wards at 8:00 am on the survey day were reviewed. Records documenting the use of antimicrobial agent were included, and total number of inpatients in each ward was recorded to estimate antimicrobial use. Patients admitted after 8:00 am on the survey day were excluded. Patients receiving ongoing antimicrobial regimens (e.g., every 24 hours, every 48 hours or once weekly) were also included. Additional details of PPS methodology for hospitalised patients have been described previously [19]. All outpatient (OPD) records and prescriptions from patients attending the paediatric, medicine, and surgical OPDs of all participating hospitals between 8:00 am and 4:00 pm on the survey days were screened for antimicrobial prescriptions. If the same outpatient turned for consultation during the same survey period, this patient was included in the survey and each visit was defined as a new consultation. Topical antimicrobials were not included in the antimicrobial use calculation for both OPD and hospitalised patients. Data were retrospectively collected for five OPD consultation days (excluding weekends) to ensure that the number of prescriptions was representative of routine OPD activity.”

* Causal language - In trials, there is usually a distinction in the language in terms of causal vs associational for primary and secondary trial outcomes. It would be beneficial to use associational language in the discussion and other sections for secondary outcomes.
**Response**: We have reviewed the manuscript and revised the language to use associational wording for secondary outcomes and in the Discussion. For example, lines **484-492**:

“Low adherence to guidelines in the surgical and obstetrics and gynaecology departments contributed to the overall low adherence observed in this study. A possible explanation for this could be concerns about surgical site infections related to perceptions about the cleanliness of the hospital environment, which may be associated with extended durations of antimicrobial prophylaxis or the unnecessary combination of antibiotics for broader coverage, even for routine procedures, such as uncomplicated vaginal deliveries, where prophylaxis is not required. Possible strategies to address this issue include improving clinical knowledge and training, monitoring prescribing practices and giving feedback, and implementing decision support systems for prescribers. Further research is needed to better understand the factors associated with this prescribing behaviour, to inform context-appropriate interventions these groups.”

Also lines **510-519**:

“The COVID-19 pandemic may have been associated with low adherence in this study. The intervention was implemented during the peak of COVID-19 in Laos. This period was characterised by changes in hospital operations, including the relocation of many doctors to COVID-19 centres, which may have limited their exposure to the App-G and left them unavailable for training sessions. Hospital patient numbers also decreased, with as few as eight patients recorded in a single survey in Luang Namtha. Additionally, some wards were designated for COVID-19 case management. This unexpected situation may have influenced prescribing patterns and guidelines uptake. However, data from routine PPS conducted after the trial has showed similar patterns, suggesting that COVID-19 was unlikely to be the main determinant of the observed findings. Additionally, the app and AMS training were delivered once per hospital, which may have limited familiarity and confidence in using the app. Repeated trainings on an annual basis could be considered to support sustained use.”

* Thank you for providing the CONSORT checklist. The CONSORT flowchart should be figure 1, please revise.
**Response**: Thank you for this comment. The CONSORT flowchart is currently presented as Figure 1 in the manuscript. We have reviewed the manuscript and confirmed that the figure numbering is consistent throughout.

* I am not able to find protocol attached in current version of the manuscript. Please check carefully all supporting documents and reorganize if necessary.

**Response**: Thank you. We have included the study protocol in the Supplementary Material (S1 Text) and added information in the Method section (line **165-166**): “This trial was registered at clinicaltrials.gov (NCT04914793), and the study proposal is provided in S1 Text. …”
