# Supplementary material for: Adherence to smartphone-delivered antimicrobial prescribing guidelines in hospitals in Lao PDR: A stepped-wedge cluster randomized controlled trial
Source: PLoS Med. 2026 Aug 27;23(8):e1005205. doi: 10.1371/journal.pmed.1005205 (PMC13521649; doi:10.1371/journal.pmed.1005205)
Supplement: Supplementary file 19 [file pmed.1005205.s031.docx]

**Response to Editor and Reviewer:**
Dear Editor and Reviewer,

Re: "Adherence to smartphone-delivered antimicrobial prescribing guidelines in hospitals in Lao PDR: A stepped-wedge cluster randomized controlled trial" (PMEDICINE-D-25-03563R3)

We would like to thank you for your time and effort in reviewing this manuscript. Please find our point-by-point responses to your comments below.

**ABSTRACT**

* The Abstract must be CONSORTed please indicate if the trial met pre-specified endpoints

**Response**: Thank you for highlighting this point. The Abstract conclusion now clarifies the smartphone application intervention did not demonstrate an improvement in the proportion of antimicrobial prescriptions adherent to prescribing guidelines compared with paper-based guidelines (lines **79-82**):

“This study demonstrated that smartphone technology can deliver antimicrobial prescribing guidelines in an LMIC like Laos. However, the trial did not demonstrate the pre-specified improvement in guidelines adherence with the smartphone app and AMS training compared with paper-based guidelines. While treatment guidelines underpin antimicrobial stewardship efforts, adherence depends on multiple factors beyond guideline availability …”

**AUTHOR SUMMARY**

* In the subsection "What Did the Researchers Do and Find?" Authors should briefly describe the study design that was used and the study’s major findings. Do include the headline numbers from the study, such as the sample size and key findings. [https://journals.plos.org/plosmedicine/s/revising-your-manuscript#loc-author-summary](https://url.uk.m.mimecastprotect.com/s/LrS9CGYnGCJp9vwhrHPfBfFez?domain=journals.plos.org).

**Response**: Thank you for your suggestion. We have revised “What did the research do and find?” section of the Author summary for clarity (lines 92-102):

“**What did the researchers do and find?**

- We conducted a stepped-wedge cluster randomized controlled trial in six hospitals across Laos to compare adherence to newly introduced antimicrobial prescribing guidelines delivered via a smartphone application, with training on antimicrobial use principles and app use, versus a paper-based version, with training on the use. Regular point prevalence surveys were conducted to monitor antimicrobial prescribing.
- A total of 3,561 inpatient and 3,905 outpatient antimicrobial prescriptions were assessed between 2021 and 2022. Guideline adherence remained low and did not differ between hospitals using the paper-based guidelines and those using the app-based version alongside training on antimicrobial use principles. Low guideline adherence was partly explained by the persistently high proportion of inappropriate antimicrobial use for surgical and obstetric prophylaxis.”

* In the limitation bullet point (lines 113-115), please rewrite so it clearly states for potential readers that this is a stud limitation.

**Response**: Thank you for your suggestion. We have revised the limitation bullet point in the Author summary section to clearly state that this is a study limitation, based on your feedback (lines **114-116**):

“An important limitation of this study is that prescribing was assessed using patients' medical records, and prescriptions could not be reliably linked to individual prescribers. Therefore, changes in prescribing behaviour at the individual prescriber level after the guideline implementation could not be evaluated. “

**METHODS**

* I do understand that there is not patient registration in your trial but could you provide timeframes for patient data you used (could be in MMM-YYYY format). I think this will add to the reporting.
**Response:** Thank you for this helpful suggestion. We have clarified the timeframe of the patient data use in this study. We have revised the Result section to describe the timing of each round of point prevalence surveys during the trial period, with reference of Figure 1 and Figure 3, to improve the clarity of the study timeline (Lines **290-299**):

“The trial operated over 22 months, from January 2021 to October 2022 (Figure 1 and Figure 3). Patients prescribing data were collected during five rounds of PPSs conducted throughout the trial period. The baseline survey was conducted between January and March 2021 before the implementation of PP-G. The second survey was conducted in June-July 2021, immediately before the implementation of the App-G in the first group of hospitals (XK and LNT; sequence-1). The third survey was conducted in November-December 2021, before App-G implementation in the second group (SV and SVK; sequence-2). The fourth survey was conducted in April 2022, before App-G implementation in the final group of hospitals (VTP and MS; sequence-3). The final survey was conducted between September and October 2022, by which time all hospitals had had access to App-G for at least five-to-six-months. The interval between surveys was approximately five to six months, which was longer than expected due to COVID-19 pandemic restrictions.”

* Thank you for providing CONSORT Checklist. Could you revise it so it does not include page numbers (use section and paragraph numbers as for some points already)
**Response**: Thank you. We have revised the CONSORT Checklist according to your suggestions.

* Figure S2 "Lao AntiMicrobial Prescribing Application (LAMPA) trial profile for outpatients" should be move to main text of manuscript as this is also trial profile but for outpatients. Figure 1 flowchart is correctly placed but I think that flowchart for outpatients should also be in the main body of the manuscript. If this will be amended, please revise manuscript for Figure references.
**Response**: Thank you for your suggestion. We agree that it is more appropriate to place the trial profile for outpatients in the main text of the manuscript. In the original version, we were concerned about the readability and therefore included it in the Supplementary Material. In the revised manuscript, the outpatient trial profile has been moved to the main text as Figure 3. We have also updated figure references and revised the manuscript accordingly.

**Comments from Reviewers:**
Reviewer #2: The authors have addressed all my comments, and I am happy to recommend acceptance. Well done and congratulations!

**Response**: Thank you very much for your comments and encouragement. We appreciate your positive feedback and your support throughout the review process.
