# Supplementary material for: Adherence to smartphone-delivered antimicrobial prescribing guidelines in hospitals in Lao PDR: A stepped-wedge cluster randomized controlled trial
Source: PLoS Med. 2026 Aug 27;23(8):e1005205. doi: 10.1371/journal.pmed.1005205 (PMC13521649; doi:10.1371/journal.pmed.1005205)
Supplement: Peer review history

# Editor report: Decision Letter 0

**DOI:** 10.1371/journal.pmed.1005205.r001

**Authors:** Heather Van Epps

11 Dec 2025

Dear Dr Chansamouth,

Thank you for submitting your manuscript entitled "Assessment of adherence to antimicrobial prescribing guidelines delivered by a smartphone application in hospitals in Lao PDR: Stepped-wedge cluster randomised controlled trial" for consideration by PLOS Medicine.

Your manuscript has now been evaluated by the PLOS Medicine editorial staff, and I am writing to let you know that we would like to send your submission out for external peer review.

However, before we can send your manuscript to reviewers, we ask that you add the details explaining the late registration to the paper (Methods) for full transparency. We also need you to complete your submission by providing the metadata that is required for full assessment. To this end, please login to Editorial Manager where you will find the paper in the 'Submissions Needing Revisions' folder on your homepage. Please click 'Revise Submission' from the Action Links and complete all additional questions in the submission questionnaire.

For clinical studies, please upload a copy of your trial study protocol as a supporting information file. The study protocol should be the version submitted for approval to the institutional review board or ethics committee, should include any amendments to the study protocol, as well as the date of their approval by the institutional review or ethics committee. Please also detail any deviations from the study protocol in the Methods section of your manuscript. The editors will consider the protocol and study conduct prior to a final decision for external review.

Please re-submit your manuscript within two working days, i.e. by Dec 15 2025 11:59PM.

Login to Editorial Manager here: https://www.editorialmanager.com/pmedicine

Once your full submission is complete, your paper will undergo a series of checks in preparation for peer review. Once your manuscript has passed all checks it will be sent out for review.

Feel free to email us at plosmedicine@plos.org if you have any queries relating to your submission.

Kind regards,

Heather Van Epps, PhD

Consulting Editor

PLOS Medicine

# Peer review: Decision Letter 1

**DOI:** 10.1371/journal.pmed.1005205.r002

**Authors:** Katarzyna Starowicz

31 Mar 2026

Dear Dr Chansamouth,

Many thanks for submitting your manuscript "Assessment of adherence to antimicrobial prescribing guidelines delivered by a smartphone application in hospitals in Lao PDR: Stepped-wedge cluster randomised controlled trial" (PMEDICINE-D-25-03563R1) to PLOS Medicine. The paper has been reviewed by subject experts and a statistician; their comments are included below and can also be accessed here: [LINK]

As you will see, the reviewers and the academic editor finds your study important for global health issue of antimicrobial resistance. After discussing the paper with the editorial team and an academic editor with relevant expertise, I'm pleased to invite you to revise the paper in response to the reviewers' comments. We plan to send the revised paper to some or all of the original reviewers, and we cannot provide any guarantees at this stage regarding publication.

In addition to these revisions, you may need to complete some formatting changes, which you will receive in a follow up email. A member of our team will be in touch with a set of requests shortly. If you do not receive a separate email within a few days, please assume that checks have been completed, and no additional changes are required.

When you upload your revision, please include a point-by-point response that addresses all of the academic editor, reviewer and editorial points, indicating the changes made in the manuscript and either an excerpt of the revised text or the location (eg: page and line number) where each change can be found. Please also be sure to check the general editorial comments at the end of this letter and include these in your point-by-point response. When you resubmit your paper, please include a clean version of the paper as the main article file and a version with changes tracked as a marked-up manuscript. It may also be helpful to check the guidelines for revised papers at http://journals.plos.org/plosmedicine/s/revising-your-manuscript for any that apply to your paper.

We ask that you submit your revision by Apr 21 2026 11:59PM. However, if this deadline is not feasible, please contact me by email, and we can discuss a suitable alternative.

Don't hesitate to contact me directly with any questions (kstarowicz@plos.org).

Best regards,

Katarzyna

Katarzyna Starowicz, PhD

Associate Editor

PLOS Medicine

kstarowicz@plos.org

-----------------------------------------------------------

Comments from the academic editor:

This study addresses a very relevant global issue of antimicrobial resistance. The authors should be commended in conducting a trial to optimize antimicrobial prescribing in a low-middle-income country where antimicrobial resistance is a major issue.

Despite being a negative study, I think the results are very insightful and highlight the knowledge to practice gap. Although there were available guidelines published, which were provided to the physicians in easily accessible formats, the overall adherence remained quite low. The use of technology (i.e. smartphone app) did not significantly improve adherence, which suggest that improving accessibility to guidelines likely is not enough to improve adherence to guidelines. Therefore, policy makers should consider a multimodal implementation approach. I think the results can still be quite useful to policy makers as a caution that improving access to guidelines through technology likely does not equate to high uptake, usage or adherence to the guidelines.

There are many limitations to the study. In my opinion, the two main limitations are

1) sample size calculation that is not reproducible and inclusion of only 6 sites that made the study quite underpowered such that there could not be any definitive conclusion.

2) Use of point prevalence survey of antimicrobial use, which is such a small short time window of data collection compared to the entire period that may not be representative of the entire period - authors should acknowledge this as a limitation in the discussion

The authors will absolutely need to address the comments from the rigorous statistical review adequately.

In addition to the reviewer comments, I have 2 major comments for authors:

- There is nowhere in the manuscript or references available of what the national guidelines actually recommend. The authors should provide the guidelines in the appendix or at a minimum a summary of the antibiotic recommendations so that at least the readers can get a sense of what these recommendations are and how valid they are.

- The authors had acknowledged that most studies defined appropriateness based on antibiotic choice alone. This study used a much more stringent criteria of also considering dose (and duration for prophylaxis). Clinically, there are many valid reasons to use a different dose based on patient factors (e.g. renal function, weight) and the fact that many antibiotics recommended dose is actually a range. I think the authors should consider doing a sensitivity analysis of judging appropriateness without the dose criteria.

-----------------------------------------------------------

Comments from the reviewers:

Reviewer #1: Article: Assessment of adherence to antimicrobial prescribing guidelines delivered by a smartphone application in hospitals in Lao PDR: Stepped-wedge cluster randomised controlled trial

Strengths:

1.Relevance: The study addresses a critical issue in global health—antimicrobial resistance (AMR)—and proposes an innovative solution for a low- and middle-income country (LMIC), Laos. It explores the potential of mobile health technologies in improving guideline adherence in healthcare systems with limited resources.

2.Study Design: The stepped-wedge cluster randomized controlled trial (SW-CRT) design is robust, allowing for a clear comparison between the app-based intervention and the paper-based guidelines, with randomization across six hospitals, enhancing the generalizability of the findings.

3.Data Analysis: The use of mixed-effects regression models to account for time and clustering effects provides a sophisticated approach to analyzing the data. This method is appropriate for handling the complexities of clustered data in a longitudinal trial.

4.Contribution to AMR Control: The study offers important insights into antimicrobial stewardship in Laos, a country where AMR is becoming an increasing concern. It provides a foundation for future interventions and highlights the challenges of improving adherence to guidelines even with technological interventions.

5.Multi-level Outcomes: The study considers multiple relevant outcomes, including prescribing patterns, adherence to guidelines, and the impact on antimicrobial consumption, which are crucial for assessing the effectiveness of the intervention in real-world settings.

Weaknesses:

1.Limited Evidence of Impact: Despite the innovative approach, the study finds insufficient evidence that the smartphone app improved adherence to guidelines compared to paper-based methods. This suggests that simply providing a mobile app is not enough to change prescribing behavior, which may limit the practical implications of the findings.

2.Low Adherence Rates: The overall adherence rates, even with the intervention, are quite low (17% with paper-based and 25.6% with the app). This suggests that more complex, multifaceted interventions beyond just guideline delivery are needed to address the challenges of AMR.

3.Potential Confounding Variables: While the study adjusts for some confounders, the lack of deeper exploration into other factors influencing adherence (e.g., prescriber behavior, institutional support, availability of resources, COVID scenario, percentage of prescribers familiar with using mobile tools) may have affected the outcomes.

4.Generalizability: The study's findings are based on a small number of hospitals in Laos, which may limit the generalizability of the results to other LMICs or even within different regions of Laos. More extensive studies across diverse settings could provide a clearer picture of the app's potential.

5.Implementation Challenges: The study does not extensively discuss the barriers to effective implementation of the smartphone app, such as technological literacy, smartphone availability, or infrastructure limitations, which are critical in LMICs like Laos.

6.I missed having more clear and objective information about the app itself, its features, the layout, and the number of people who downloaded it, as well as the app´s promotion to the prescribers.

Reviewer #2: This manuscript presents a stepped wedge trial examining the implementation of antimicrobial prescribing guidelines via paper and app in Laos. The study did not find evidence of improved adherence to guidelines in either delivery format. The study also provides an important baseline of antimicrobial use outside of high-income settings.

The manuscript is well written and does an excellent job of contextualising the research and establishing its importance. Unfortunately, I found the methodological and statistical detail insufficiently comprehensive and clear. I also suggest the Results section could do with editing to improve readability.

Detailed feedback on the manuscript is provided below. My primary focus has been methodological, but I have made a few broader points as well. All items are major unless indicated otherwise.

1.I'm afraid I don't understand the authors' use of the term "point prevalence survey" throughout the manuscript. Does this mean all prescribing data over the course of the trial was not used? The authors say point prevalence surveys were conducted four-monthly but the periods in Figure 1 are one, two, or three months, so I don't yet understand how the surveys and the step wedge stages interrelate. It is crucial to communicate these characteristics of the trial as clearly and simply as possible, particularly for a more complex design like a stepped-wedge CRT.

2.I suggest the authors provide a small amount of detail on what a stepped wedge trial is, how it differs from a traditional cluster randomised trial, and why its use is appropriate for this research. I also suggest including a reference for more details on this type of study design for readers who are unfamiliar (e.g., https://www.bmj.com/content/350/bmj.h391 or similar).

3.I'd suggest against using the word "pair" in the "Randomisation and masking" section (lines 164-170). Generally when "pair" is used in the context of randomisation, it means paired samples and this left me very confused. Suggest using "groups of two" or similar instead.

4.Also in this section, I found the phrase "Hospital pairs were assigned to interventions start dates using simple random sampling…" confusing as it suggests the actual date of implementation was chosen at random. I think the authors mean the implementation sequence of the hospital groups was randomised?

5.Some wording in the Outcomes section seemed to contradict itself. The authors say "Full adherence with the guidelines was defined as the prescriptions with correct antimicrobial agent(s) and correct dose." but then go on to say "Non-adherence to the guidelines was defined as using a different antimicrobial agent(s) compared to that suggested in the provided antimicrobial prescribing guidelines." This means it's unclear if using the correct agent but the incorrect dose is adherent or not. Suggest the authors revise this paragraph to ensure the rules governing whether a prescription was within guidelines or not are abundantly clear.

6.I am also a bit confused about the descriptions in the Procedures section. The authors say switching happened approximately every four months resulting in a 1:1 ratio of intervention to reference period of observation but this does not seem to be what happened. Perhaps softening the language by saying "The original plan…" or "The intention was…", then the authors can explain what actually happened (COVID delays etc.) in the Results without this conflicting with the Methods.

7.I was not able to replicate the sample size calculations based on the information provided using the Stata steppedwedge tool referenced. Please provide sufficient detail in a form that maps directly to the inputs required for the Stata steppedwedge tool to allow confirmation of the sample size calculation. The authors could also consider including the relevant Stata code and output as a supplement.

8.The descriptions of the statistical modelling approaches in the Statistical Analysis section of the Methods are a good start, but more detail is needed. If modelling results are presented, then enough detail should be included in the Methods so an interested reader can replicate the analyses. Specifically, while the authors have noted the model type they have used ( "mixed-effect logistic regression model"), it's important to clearly state the variables in the models (fixed and random effects for hierarchical models, cluster variables for GEE etc.). Of particular importance is how hierarchies are handled e.g., prescription within patient within hospital, as this can cause biased estimates if not modelled correctly. Including abridged modelling code as a supplement can also help when more complex analyses are being undertaken.

9.Related to the prior point, given only six hospitals were included, I suggest the authors consider the risk of unreliable standard errors and p-values arising from the combination of GLMMs and a small number of clusters ( https://www.tandfonline.com/doi/full/10.1080/00273171.2016.1236237 ). Specific guidance for stepped wedge trials examining binary endpoints is available ( https://link.springer.com/article/10.1186/s13063-017-1862-2 ), and I suggest the authors clearly communicate that they considered this risk and their decision-making around the analytical approach chosen (e.g., choice of test/CI method).

10.I also found the use of the word "considered" in this section confusing. I suggest the authors clearly state the models that were actually run (and exclude those that were only considered), ensuring this matches the presented results.

11.What is meant by "WHO AWaRe Access group antibiotics"? This is mentioned for the first time in the Results with no background in the Methods. Please ensure that all presented results are based on already introduced methods. Please also provide at least one reference to provide more information on external resources.

12.[MINOR] I think it's worth defining "empirical" vs. "targeted" in the Methods because otherwise a reader has to go to a table note to understand the text in the Results.

13.I'm not clear why the authors present results "… after 4-6 months of exposure…" (line 282) and what exactly is meant by this. Is this when the point prevalence survey was done? I think the data acquisition processes must be explained much more clearly.

14.[MINOR] I found the volume of results presented in the Results section to be somewhat excessive. I'd suggest the authors edit down the results presented in the text to the most salient and allowing an interested reader to examine the less salient statistics in the supplementary materials. I appreciate this is subjective, which is why I have said this point is "minor", but I found it negatively affected readability.

15.I don't quite understand the first sentence of the Discussion. I am not clear what there is "limited evidence of" and whether it is true to say the evidence is limited i.e., no reference to review literature etc. Suggest clarifying.

16.Otherwise, the Discussion is very well-written and clear. Well done!

17.I think the authors are trying to fit too much into Figure 1 - I found it very difficult to read. Suggest simplifying and focussing on the primary purpose of the figure: communicating the structure of the study.

18.[MINOR] For future reference I suggest lodging your SAP in an independent online archive like medRxiv as proof the SAP was completed prior to the study finishing.

Reviewer #3: Thank you for the opportunity to review this well written and important paper.

The need for effective stewardship is stark across several metrics- including the high percentage of inpatients receiving at least one, if not two antibiotics, and the low rate of adherence to guidelines. The paper highlights prophylaxis as an area of particular difficulty. Is there documentation of who attended training? Was the attendance among surgical and obstetric/gynae prescribers as high as on the medical/ICU wards? And was prophylaxis a focus of the training?

Presumably the paper guidelines were still accessible during the intervention period? Is there any information about how/when these were accessed, or e.g. whether the prescribers still had legible/complete copies when the surveys were carried out?

I'm not that convinced by the argument in lines 399-400. I know the qualitative outcomes are presented elsewhere, but the data about the roughly monthly accessing of the app per prescriber appears to belie it being used as an everyday clinical decision support tool. For me the main thrust of the paper is the low adherence to guidelines and that alternative/additional strategies need to be pursued in specific contexts. While guidelines may be great for AMS in some settings, it appears that here other approaches will be needed. The qualitative data and potentially some process evaluation metrics could be very helpful- I agree with the further research recommended in lines 429-430.

I'd also want more emphasis on the lack of available diagnostics- almost all treatment is empirical which again impedes effective AMS.

I think the first two sentences of the conclusion are somewhat overstated- I'm not sure the data about app usage, or the slight increase in adherence to guidelines, from a low baseline, indicates successful implementation. For me I'd emphasise the low adherence overall and the relative lack of impact in comparison to other settings, focusing on the need for nuanced, context-specific interventions for effective AMS.

Minor comments

Lines 165-168- it would be helpful to have more description about the sites- perhaps in an appendix, e.g. bed numbers, catchment areas, rural/urban to be better able to interpret the prescribing figures.

Line 276 I'd rephrase line 276: "Among the 319 prescriptions that could not be assessed" to make it clear that the following sentences refer to those 319.

Line 282- the 4-6 months of exposure here is to the intervention, not the paper guidelines also?

Line 332-333 "While the use in the PP-G group was 1,267/1,492 (84.9%, 95% CI: 83.0-86.7), and in the App-G group was 943/1,217 (77.5%; 95% CI: 75.0-79.8)." This doesn't appear to be a full sentence

Lines 487-489 about paper guidelines- this would fit better in the discussion than the conclusion.

The conclusion is quite lengthy- I'd move some of the discussion about resource constraints to the discussion also.

Figure 1 is too small- I only find it legible when I've magnified it by ~300%. It might fit better in portrait? I'd prefer to have the absolute figures and % rather than 95% CIs in this figure.

Supplementary figure 9: are the figures in brackets 95% CIs?

Supplementary figure 11: I don't understand this table- is this a comparison between app- and paper-based guideline groups or more general comparisons? It looks and reads like it is an interdepartmental comparison for example, rather than comparing between departments and guideline groups? And is it about antimicrobial usage altogether or adherence to guidelines? So, is it saying that fewer antibiotics were used in medicine, or that there was poorer adherence? And similarly, was there better adherence, or more antimicrobial use in Sequence 2?

What are VIP departments?

Supplementary figure 12: There is a lot of variability here- e.g. in the Luang Namtha site. Can you explain this? Is DDD perhaps not the best measure here?

Supplementary figure 15: It might be easier to see the comparison between the two groups if 15.1 and 15.2 were combined with the bars on the same x axis

Supplementary figure 19: looking at the timing of access- did this correspond with tea and lunch breaks? Do people find it hard to look at their phones at the time of patient review and check afterwards/before?

Supplementary figure 20: It doesn't look like the numbers here add up to the total number of searches/accessing of the app (6,239 times). What is the reason for the discrepancy?

Supplementary- typos in the CONSORT table- Methods not methos

Any attachments provided with reviews can be seen via the following link: [LINK]

--------------------------------------------------------- ---

General editorial requests:

(Note: not all will apply to your paper, but please check each item carefully)

*We ask every co-author listed on the manuscript to fill in a contributing author statement, making sure to declare all competing interests. If any of the co-authors have not filled in the statement, we will remind them to do so when the paper is revised. If all statements are not completed in a timely fashion this could hold up the re-review process. If new competing interests are declared later in the revision process, this may also hold up the submission. Should there be a problem getting one of your co-authors to fill in a statement we will be in contact. Please do not add or remove authors without first discussing this with the handling editor. You can see our competing interests policy here: http://journals.plos.org/plosmedicine/s/competing-interests.

*Please upload any figures associated with your paper as individual TIF or EPS files with 300dpi resolution at resubmission; please read our figure guidelines for more information on our requirements: http://journals.plos.org/plosmedicine/s/figures. While revising your submission, we strongly recommend that you use PLOS's NAAS tool (https://ngplosjournals.pagemajik.ai/artanalysis) to test your figure files. NAAS can convert your figure files to the TIFF file type and meet basic requirements (such as print size, resolution), or provide you with a report on issues that do not meet our requirements and that NAAS cannot fix.

After uploading your figures to PLOS's NAAS tool - https://ngplosjournals.pagemajik.ai/artanalysis, NAAS will process the files provided and display the results in the "Uploaded Files" section of the page as the processing is complete.

If the uploaded figures meet our requirements (or NAAS is able to fix the files to meet our requirements), the figure will be marked as "fixed" above. If NAAS is unable to fix the files, a red "failed" label will appear above.

When NAAS has confirmed that the figure files meet our requirements, please download the file via the download option, and include these NAAS processed figure files when submitting your revised manuscript.

*Please ensure that the paper adheres to the PLOS Data Availability Policy (see http://journals.plos.org/plosmedicine/s/data-availability), which requires that all data underlying the study's findings be provided in a repository or as Supporting Information. For data residing with a third party, authors are required to provide instructions with contact information (web or email address) for obtaining the data. Please note that a study author cannot be the contact person for the data. PLOS journals do not allow statements supported by "data not shown" or "unpublished results." For such statements, authors must provide supporting data or cite public sources that include it.

*We expect all researchers with submissions to PLOS in which author-generated code underpins the findings in the manuscript to make all author-generated code available without restrictions upon publication of the work. In cases where code is central to the manuscript, we may require the code to be made available as a condition of publication. Authors are responsible for ensuring that the code is reusable and well documented. Please make any custom code available, either as part of your data deposition or as a supplementary file. Please add a sentence to your data availability statement regarding any code used in the study, e.g. "The code used in the analysis is available from Github [URL] and archived in Zenodo [DOI link]" Please review our guidelines at https://journals.plos.org/plosmedicine/s/materials-software-and-code-sharing and ensure that your code is shared in a way that follows best practice and facilitates reproducibility and reuse. Because Github depositions can be readily changed or deleted, we encourage you to make a permanent DOI'd copy (e.g. in Zenodo) and provide the URL.

*Please ensure that the study is reported according to the CONSORT guideline and include the completed CONSORT checklist as Supporting Information. When completing the checklist, please use section and paragraph numbers, rather than page numbers. Please add the following statement, or similar, to the Methods: "This study is reported as per [XXXX] guideline (S1 Checklist)."

FORMATTING - GENERAL

*Abstract: Please structure your abstract using the PLOS Medicine headings (Background, Methods and Findings, Conclusions). Please combine the Methods and Findings sections into one section.

*At this stage, we ask that you include a short, non-technical Author Summary of your research to make findings accessible to a wide audience that includes both scientists and non-scientists. The Author Summary should immediately follow the Abstract in your revised manuscript. This text is subject to editorial change and should be distinct from the scientific abstract. Ideally each sub-heading should contain 2-3 single sentence, concise bullet points containing the most salient points from your study. In the final bullet point of 'What Do These Findings Mean?', please include the main limitations of the study in non-technical language. Please see our author guidelines for more information: https://journals.plos.org/plosmedicine/s/revising-your-manuscript#loc-author-summary.

*Please express the main results with 95% CIs as well as p values. When reporting p values please report as p<0.001 and where higher as the exact p value p=0.002, for example. Throughout, suggest reporting statistical information as follows to improve clarity for the reader "22% (95% CI [13%,28%]; p</=)". Please be sure to define all numerical values at first use.

*Please include page numbers and line numbers in the manuscript file. Use continuous line numbers (do not restart the numbering on each page).

*Please cite the reference numbers in square brackets. Citations should precede punctuation.

FIGURES AND TABLES

*Please provide titles and legends for all figures and tables (including those in Supporting Information files).

*Please define all abbreviations used in each figure/table (including those in Supporting Information files).

*Please consider avoiding the use of red and green in order to make your figure more accessible to those with color blindness.

SUPPLEMENTARY MATERIAL

*Please note that supplementary material will be posted as supplied by the authors. Therefore, please amend it according to the relevant comments outlined here.

*Please cite your Supporting Information as outlined here: https://journals.plos.org/plosmedicine/s/supporting-information

REFERENCES

*PLOS uses the numbered citation (citation-sequence) method and first six authors, et al.

*Please ensure that journal name abbreviations match those found in the National Center for Biotechnology Information (NCBI) databases (http://www.ncbi.nlm.nih.gov/nlmcatalog/journals), and are appropriately formatted and capitalised.

*Where website addresses are cited, please include the complete URL and specify the date of access (e.g. [accessed: 12/06/2023]).

*Please also see https://journals.plos.org/plosmedicine/s/submission-guidelines#loc-references for further details on reference formatting.

STUDY TYPE-SPECIFIC REQUESTS:

RCTs

*PLOS Medicine requires that all trials be prospectively registered in one of registries recognized by WHO. Please ensure that study registration details are included in the Methods section.

*Please provide explanation for the retrospective registration in the Methods section

*Please structure the Methods section using the following sub-headings: Study design and participants, Randomization and masking, Procedures, Outcomes, Statistical analysis.

*The following outcomes measures - analysis of the hospitalized and outpatients as a single group or separately - it is not very clear and appear to differ between the submitted manuscript and the protocol [and/or trial registry]. Please clarify or explain all discrepancies between the paper and protocol. If the outcomes were not prespecified in the protocol, please define them in the Methods (Outcomes section) as post hoc and explain why they were added. Post-hoc comparisons should be presented as hypothesis generating rather than conclusive.

*Please ensure that all prespecified outcomes (primary, secondary, and exploratory) are listed in the Methods/Outcomes section and indicate whether there are outcomes that are not presented in the current report.

*Please specify the dates (Month Day, Year) during which study enrollment and follow up occurred.

*Please include absolute numbers wherever you report percentages; eg, n/N (%)

*Please present the safety data for the study including numbers of specific events and whether or not adverse events are thought to be related to treatment. AEs should be reported in the abstract, per CONSORT and CONSORT-Harms.

*Please complete the CONSORT checklist (https://www.equator-network.org/reporting-guidelines/consort/) and ensure that all components of CONSORT are present in the manuscript, including how randomization was performed, allocation concealment, blinding of intervention, definition of lost to follow-up, power statement. When completing the checklist, please use section and paragraph numbers, rather than page numbers.

*Please report your abstract according to CONSORT for abstracts, following the PLOS Medicine abstract structure (Background, Methods and Findings, Conclusions) https://www.equator-network.org/reporting-guidelines/consort-abstracts/

*If your trial had to undergo important modifications in response to extenuating circumstances, please complete the CONSERVE-CONSORT checklist and provide in your Supporting Information; (https://www.equator-network.org/reporting-guidelines/guidelines-for-reporting-trial-protocols-and-completed-trials-modified-due-to-the-covid-19-pandemic-and-other-extenuating-circumstances-the-conserve-2021-statement/). When completing the checklist, please use section and paragraph numbers, rather than page numbers.

*In keeping with our commitment to Open Science, please include the study protocol document and analysis plan (including any amendments) as Supporting Information to be published with the manuscript if accepted.

*Please note that PLOS Medicine requires prospective, public registration of a data sharing plan (as part of mandatory clinical trials registration) for all clinical trials that began enrollment on or after January 1, 2019, in accordance with ICMJE requirements.

# Author comment: Author response to Decision Letter 2

**DOI:** 10.1371/journal.pmed.1005205.r003

5 May 2026

Attachment Submitted filename: Response to editors and reviewers_05May2026.docx

# Peer review: Decision Letter 2

**DOI:** 10.1371/journal.pmed.1005205.r004

**Authors:** Katarzyna Starowicz

11 Jun 2026

Dear Dr Chansamouth,

Many thanks for submitting your manuscript "Assessment of adherence to antimicrobial prescribing guidelines delivered by a smartphone application in hospitals in Lao PDR: Stepped-wedge cluster randomised controlled trial" (PMEDICINE-D-25-03563R2) to PLOS Medicine. The paper has been reviewed by subject experts and a statistician; their comments are included below and can also be accessed here: [LINK]

Your manuscript has been seen by two original reviewers and academic editor. As you will see, the reviewers raised some additional points for clarification. After discussing the paper with the editorial team and an academic editor with relevant expertise, I'm pleased to invite you to revise the paper in response to the reviewers' comments.

Please note, after reviewers comments there are listed editorial requests and study-type specific requests at the end of this letter. You must provide point-by-point response to each of them. Not providing this in form of rebuttal will significantly delay your manuscript.

In addition to these revisions, you may need to complete some formatting changes, which you will receive in a follow up email. A member of our team will be in touch with a set of requests shortly. If you do not receive a separate email within a few days, please assume that checks have been completed, and no additional changes are required.

When you upload your revision, please include a point-by-point response that addresses all of the reviewer and editorial points, indicating the changes made in the manuscript and either an excerpt of the revised text or the location (eg: page and line number) where each change can be found. Please also be sure to check the general editorial comments at the end of this letter and include these in your point-by-point response. When you resubmit your paper, please include a clean version of the paper as the main article file and a version with changes tracked as a marked-up manuscript. It may also be helpful to check the guidelines for revised papers at http://journals.plos.org/plosmedicine/s/revising-your-manuscript for any that apply to your paper.

We ask that you submit your revision by Jun 29 2026 11:59PM. However, if this deadline is not feasible, please contact me by email, and we can discuss a suitable alternative.

Don't hesitate to contact me directly with any questions (kstarowicz@plos.org).

Best regards,

Katarzyna

Katarzyna Starowicz, PhD

Associate Editor

PLOS Medicine

kstarowicz@plos.org

-----------------------------------------------------------

Comments from the reviewers:

Reviewer #2: I thank the authors for their considered and comprehensive responses to my comments and note the manuscript is reading very well. In all but two cases, I am happy to accept the authors proposed updates and consider the items finalised. However, I do suggest the authors revisit the two items below (numbers reference my original review items).

1. I think the logic in the authors response to my review item is sound, but it does not appear the manuscript was updated accordingly. I suggest incorporating the logic in the response into the manuscript. In particular, making clear that analysis of all prescribing during the study was not possible due to the absence of electronic records, so PPSs were used, with appropriate citation(s) to demonstrate acceptability of this approach.

6. The authors response to my comment and their adjustment to the manuscript are all good apart from one omission. Given the authors are relying on PPSs rather than using all prescribing data, I think it's important to reinforce that while the timeframes shifted away from the planned 4 month intervals, even where delays occurred the PPS was always conducted before each hospital transitioned to the intervention.

Reviewer #3: Thank you for your comprehensive response and rebuttal to reviewer comments. I have no further comments except:

I'm supportive of the point prevalence survey methodology as a pragmatic option in low-resource settings where daily prescribing data is likely to be sparse and challenging to access if available at all.

Thank you for redoing Figure 1- it's much clearer now.

Any attachments provided with reviews can be seen via the following link: [LINK]

--------------------------------------------------------- ---

EDITORIAL COMMENTS

* Thank you for providing the Author Summary. The Author Summary should immediately follow the Abstract in your revised manuscript. This text is subject to editorial change and should be distinct from the scientific abstract. Ideally each sub-heading should contain 2-3 single sentence, concise bullet points containing the most salient points from your study. In the final bullet point of ‘What Do These Findings Mean?’ Please include the main limitations of the study in non-technical language.

Please see our author guidelines for more information: https://journals.plos.org/plosmedicine/s/revising-your-manuscript#loc-author-summary.

* Please confirm that your title complies with PLOS Medicine's style. Your title must be nondeclarative and not a question. It should begin with main concept if possible. "Effect of" should be used only if causality can be inferred, i.e., for an RCT. Please place the study design ("A randomized controlled trial," "A retrospective study," "A modelling study," etc.) in the subtitle (ie, after a colon).

* Please confirm that your abstract complies with our requirements, including format (three sections: Background, Methods and Findings, and Conclusions) and providing all the information relevant to this study type https://journals.plos.org/plosmedicine/s/submission-guidelines#loc-abstract

* Please ensure that the Introduction ends with a clear description of the study question or hypothesis.

* Please ensure that all abbreviations are defined at first use throughout the text.

* Please confirm that all numbers presented in the abstract are present and identical to numbers presented in the main manuscript text.

GENERAL

* Please disclose any use of AI tools and technologies to the study or article content at the end of the Methods. Please specify the name(s) and version(s) of any tool(s) used and a description of their use, including how the AI-assisted or generated content was evaluated by authors. Please also include a statement specifying who retains responsibility for the final manuscript. Please see our guidelines for more information: https://journals.plos.org/plosmedicine/s/ethical-publishing-practice#loc-artificial-intelligence-tools-and-technologies.

* Please review your text for claims of novelty or primacy (e.g. 'for the first time') and remove this language. In addition, please check that any use of statistical terms (such as trend or significant) are supported by the data, and if not please remove them.

* Please remove the 'conclusions' subheading from the discussion. Please also remove any other subheadings from the discussion.

* Statistical reporting: Please revise throughout the manuscript, including tables and figures.

- Please report statistical information as follows to improve clarity for the reader ""22% (95% CI [13,28]; p</=)"".

- Please separate upper and lower bounds with commas instead of hyphens as the latter can be confused with reporting of negative values.

- Please repeat statistical definitions (HR, CI etc.) for each set of parentheses.

* In the last sentence of the Abstract Methods and Findings section, please describe the main limitation(s) of the study's methodology.

* In the abstract, please include the important dependent variables that are adjusted for in the analyses.

FUNDING STATEMENT

* The funding statement should include: specific grant numbers, initials of authors who received each award, URLs to sponsors’ websites. Also, please state whether any sponsors or funders (other than the named authors) played any role in study design, data collection and analysis, the decision to publish, or preparation of the manuscript. If they had no role in the research, include this sentence: “The funders had no role in study design, data collection and analysis, decision to publish, or preparation of the manuscript.”

COMPETING INTERESTS STATEMENT

* All authors must declare their relevant competing interests per the PLOS policy, which can be seen here: https://journals.plos.org/plosmedicine/s/competing-interests For authors with ties to industry, please indicate whether any of the interests has a financial stake in the results of the current study.

*Please amend your competing interests statement. Funding information should go to the relevant section and include URLs.

DATA AVAILABILITY

* PLOS Medicine requires that the de-identified data underlying the specific results in a published article be made available, without restrictions on access, in a public repository or as Supporting Information at the time of article publication, provided it is legal and ethical to do so. Please see the policy at http://journals.plos.org/plosmedicine/s/data-availability and FAQs at

http://journals.plos.org/plosmedicine/s/data-availability#loc-faqs-for-data-policy

* PLOS defines the “minimal data set” to consist of the data set used to reach the conclusions drawn in the manuscript with related metadata and methods, and any additional data required to replicate the reported study findings in their entirety. Authors do not need to submit their entire data set, or the raw data collected during an investigation. Please submit the following data:

The values behind the means, standard deviations and other measures reported;

The values used to build graphs;

The points extracted from images for analysis.

* Please include the statement on code availability in the data availability statement. Please see guidance here:https://journals.plos.org/plosmedicine/s/code-sharing-guidance#loc-data-availability-statement-examples

ETHICS AND CONSENT

* Please revise for use of patient-centered language. Please note that patient-centered language is constructed with the use of post-modified nouns (e.g. 'patients with psoriasis’ (or similar) instead of ‘psoriasis patients’) putting the person first in the sentence structure.

FIGURES

* Please ensure that where relevant figures include 95% CIs.

* Please show graph axes beginning at zero. If this is not possible, please show a break in the axis.

* Where data points are discrete, please ensure that they are depicted in the figures as discrete data and not as a continuous line.

* Please confirm that the appropriate usage rights apply to the use of this map. Please see our guidelines for map images: https://journals.plos.org/plosmedicine/s/figures#loc-maps

* Please provide the unadjusted comparisons as well as the adjusted comparisons in all relevant Tables

* Please specify the variables controlled for in all relevant Tables

INCLUSIVITY IN GLOBAL RESEARCH

PLOS has a 'Inclusivity in Global Research' policy which aims to promote collaboration and inclusivity in global health research. You are required to complete PLOS’ questionnaire on inclusivity in global research and submit it with your revised paper. The policy and questionnaire can be found at https://journals.plos.org/plosone/s/best-practices-in-research-reporting.

ACKNOWLEDGMENTS

* Please include an acknowledgment of study participants.

CLINICAL TRIALS specific

* Some of the outcome measures or methods appear to differ between the submitted manuscript and the trial registry and/or protocol. Please clarify and explain the discrepancy.

* The following outcomes measures - analysis of the hospitalized and outpatients as a single group or separately - it is not very clear and appear to differ between the submitted manuscript and the protocol [and/or trial registry]. Please clarify or explain all discrepancies between the paper and protocol.

* Thank you for attaching SAP to your submission. Although, whole plan is attached could you please provide more detailed information in the methods section of the mansuscript.

* Please, provide inclusion/exclusion criteria in a manuscript to match the protocol

* Causal language - In trials, there is usually a distinction in the language in terms of causal vs associational for primary and secondary trial outcomes. It would be beneficial to use associational language in the discussion and other sections for secondary outcomes.

* Thank you for providing the CONSORT checklist. The CONSORT flowchart should be figure 1, please revise.

* I am not able to find protocol attached in current version of the manuscript. Please check carefully all supporting documents and reorganize if necessary.

# Author comment: Author response to Decision Letter 3

**DOI:** 10.1371/journal.pmed.1005205.r005

29 Jun 2026

Attachment Submitted filename: Response to editors and reviewers_V2.0_29June2026.docx

# Peer review: Decision Letter 3

**DOI:** 10.1371/journal.pmed.1005205.r006

**Authors:** Katarzyna Starowicz

8 Jul 2026

Dear Dr. Chansamouth,

Thank you very much for re-submitting your manuscript "Adherence to smartphone-delivered antimicrobial prescribing guidelines in hospitals in Lao PDR: A stepped-wedge cluster randomized controlled trial" (PMEDICINE-D-25-03563R3) for review by PLOS Medicine.

I have discussed the paper with my colleagues and the academic editor and it was also seen again by one reviewer. I am pleased to say that provided the remaining editorial and production issues are dealt with we are planning to accept the paper for publication in the journal.

The remaining issues that need to be addressed are listed at the end of this email. Please address all of them and provide rebuttal to avoid any delays. Any accompanying reviewer attachments can be seen via the link below. Please take these into account before resubmitting your manuscript:

[LINK]

***Please note while forming your response, if your article is accepted, you may have the opportunity to make the peer review history publicly available. The record will include editor decision letters (with reviews) and your responses to reviewer comments. If eligible, we will contact you to opt in or out.***

In revising the manuscript for further consideration here, please ensure you address the specific points made by each reviewer and the editors. In your rebuttal letter you should indicate your response to the reviewers' and editors' comments and the changes you have made in the manuscript. Please submit a clean version of the paper as the main article file. A version with changes marked must also be uploaded as a marked up manuscript file.

Please also check the guidelines for revised papers at http://journals.plos.org/plosmedicine/s/revising-your-manuscript for any that apply to your paper. If you haven't already, we ask that you provide a short, non-technical Author Summary of your research to make findings accessible to a wide audience that includes both scientists and non-scientists. The Author Summary should immediately follow the Abstract in your revised manuscript. This text is subject to editorial change and should be distinct from the scientific abstract.

In addition to these revisions, you may need to complete some formatting changes, which you will receive in a follow up email. A member of our team will be in touch with a set of requests shortly. If you do not receive a separate email within a few days, please assume that checks have been completed, and no additional changes are required.

We expect to receive your revised manuscript within 1 week. Please email us (plosmedicine@plos.org) if you have any questions or concerns.

We ask every co-author listed on the manuscript to fill in a contributing author statement. If any of the co-authors have not filled in the statement, we will remind them to do so when the paper is revised. If all statements are not completed in a timely fashion this could hold up the re-review process. Should there be a problem getting one of your co-authors to fill in a statement we will be in contact. YOU MUST NOT ADD OR REMOVE AUTHORS UNLESS YOU HAVE ALERTED THE EDITOR HANDLING THE MANUSCRIPT TO THE CHANGE AND THEY SPECIFICALLY HAVE AGREED TO IT.

Please ensure that the paper adheres to the PLOS Data Availability Policy (see http://journals.plos.org/plosmedicine/s/data-availability), which requires that all data underlying the study's findings be provided in a repository or as Supporting Information. For data residing with a third party, authors are required to provide instructions with contact information for obtaining the data. PLOS journals do not allow statements supported by "data not shown" or "unpublished results." For such statements, authors must provide supporting data or cite public sources that include it.

To enhance the reproducibility of your results, we recommend that you deposit your laboratory protocols in protocols.io, where a protocol can be assigned its own identifier (DOI) such that it can be cited independently in the future. Additionally, PLOS ONE offers an option to publish peer-reviewed clinical study protocols. Read more information on sharing protocols at https://plos.org/protocols?utm_medium=editorial-email&utm_source=authorletters&utm_campaign=protocols

Please review your reference list to ensure that it is complete and correct. If you have cited papers that have been retracted, please include the rationale for doing so in the manuscript text, or remove these references and replace them with relevant current references. Any changes to the reference list should be mentioned in the rebuttal letter that accompanies your revised manuscript.

Please note, when your manuscript is accepted, an uncorrected proof of your manuscript will be published online ahead of the final version, unless you've already opted out via the online submission form. If, for any reason, you do not want an earlier version of your manuscript published online or are unsure if you have already indicated as such, please let the journal staff know immediately at plosmedicine@plos.org.

If you have any questions in the meantime, please contact me or the journal staff on plosmedicine@plos.org.

We look forward to receiving the revised manuscript by Jul 15 2026 11:59PM.

Sincerely,

Katarzyna Starowicz, PhD

Associate Editor

PLOS Medicine

plosmedicine.org

------------------------------------------------------------

Requests from Editors:

ABSTRACT

* The Abstract must be CONSORTed please indicate if the trial met pre-specified endpoints

AUTHOR SUMMARY

* In the subsection "What Did the Researchers Do and Find?" Authors should briefly describe the study design that was used and the study’s major findings. Do include the headline numbers from the study, such as the sample size and key findings. https://journals.plos.org/plosmedicine/s/revising-your-manuscript#loc-author-summary.

* In the limitation bullet point (lines 113-115), please rewrite so it clearly states for potential readers that this is a stud limitation.

METHODS

* I do understand that there is not patient registration in your trial but could you provide timeframes for patient data you used (could be in MMM-YYYY format). I think this will add to the reporting.

* Thank you for providing CONSORT Checklist. Could you revise it so it does not include page numbers (use section and paragraph numbers as for some points already)

* Figure S2 "Lao AntiMicrobial Prescribing Application (LAMPA) trial profile for outpatients" should be move to main text of manuscript as this is also trial profile but for outpatients. Figure 1 flowchart is correctly placed but I think that flowchart for outpatients should also be in the main body of the manuscript. If this will be amended, please revise manuscript for Figure references.

-------------------------------------

Comments from Reviewers:

Reviewer #2: The authors have addressed all my comments, and I am happy to recommend acceptance. Well done and congratulations!

Any attachments provided with reviews can be seen via the following link:

[LINK]

# Author comment: Author response to Decision Letter 4

**DOI:** 10.1371/journal.pmed.1005205.r007

13 Jul 2026

Attachment Submitted filename: Response to Editor_V3.0_13July2026.docx

# Editor report: Decision Letter 4

**DOI:** 10.1371/journal.pmed.1005205.r008

**Authors:** Katarzyna Starowicz

22 Jul 2026

Dear Dr Chansamouth,

On behalf of my colleagues and the Academic Editor, Anthony D. Bai, I am pleased to inform you that we have agreed to publish your manuscript "Adherence to smartphone-delivered antimicrobial prescribing guidelines in hospitals in Lao PDR: A stepped-wedge cluster randomized controlled trial" (PMEDICINE-D-25-03563R4) in PLOS Medicine.

Before your manuscript can be formally accepted you will need to complete some formatting changes, which you will receive in a follow up email. Please be aware that it may take several days for you to receive this email; during this time no action is required by you. Once you have received these formatting requests, please note that your manuscript will not be scheduled for publication until you have made the required changes.

In the meantime, please log into Editorial Manager at http://www.editorialmanager.com/pmedicine/, click the "Update My Information" link at the top of the page, and update your user information to ensure an efficient production process.

PRESS

We frequently collaborate with press offices. If your institution or institutions have a press office, please notify them about your upcoming paper at this point, to enable them to help maximise its impact. If the press office is planning to promote your findings, we would be grateful if they could coordinate with medicinepress@plos.org. If you have not yet opted out of the early version process, we ask that you notify us immediately of any press plans so that we may do so on your behalf.

We also ask that you take this opportunity to read our Embargo Policy regarding the discussion, promotion and media coverage of work that is yet to be published by PLOS. As your manuscript is not yet published, it is bound by the conditions of our Embargo Policy. Please be aware that this policy is in place both to ensure that any press coverage of your article is fully substantiated and to provide a direct link between such coverage and the published work. For full details of our Embargo Policy, please visit http://www.plos.org/about/media-inquiries/embargo-policy/.

To enhance the reproducibility of your results, we recommend that you deposit your laboratory protocols in protocols.io, where a protocol can be assigned its own identifier (DOI) such that it can be cited independently in the future. Additionally, PLOS ONE offers an option to publish peer-reviewed clinical study protocols. Read more information on sharing protocols at https://plos.org/protocols?utm_medium=editorial-email&utm_source=authorletters&utm_campaign=protocols

Thank you again for submitting to PLOS Medicine. We look forward to publishing your paper.

Sincerely,

Katarzyna Starowicz, PhD

Associate Editor

PLOS Medicine
